# Diversity pattern of insects from Macao based on an updated species checklist after 25 years

**DOI:** 10.3897/BDJ.12.e118110

**Published:** 2024-04-05

**Authors:** Chunlan Xian, Chi Man Leong, Jiuyang Luo, Fenglong Jia, Hongxiang Han, Qiang Xie

**Affiliations:** 1 School of Life Sciences, State Key Laboratory of Biocontrol, Sun Yat-sen University, Guangzhou, China School of Life Sciences, State Key Laboratory of Biocontrol, Sun Yat-sen University Guangzhou China; 2 Department of Life Sciences, Faculty of Science and Technology, Beijing normal university – Hong Kong Baptist University United International College, Zhuhai, China Department of Life Sciences, Faculty of Science and Technology, Beijing normal university – Hong Kong Baptist University United International College Zhuhai China; 3 Macao Entomological Society, Estrada Coronel Nicolau de Mesquita, Macao SAR, China Macao Entomological Society, Estrada Coronel Nicolau de Mesquita Macao SAR China; 4 Key Laboratory of Zoological Systematics and Evolution, Institute of Zoology, Chinese Academy of Sciences, Beijing, China Key Laboratory of Zoological Systematics and Evolution, Institute of Zoology, Chinese Academy of Sciences Beijing China

**Keywords:** biodiversity, insect, checklist, fauna Macao

## Abstract

**Background:**

Insects represent one of the most diverse groups in the organism world with extremely rich species and morphological diversity, playing important roles in natural and city ecosystems. Regional compilation of insect species lists helps to clarify the richness of insect species in a region, enhances our understanding the structure and function of a local ecosystem and promotes the protection and development of insect resources. Moreover, it also serves as a valuable reference for cities with small area, large population and high urbanisation like Macao. Macao (Macau) Special Administrative Region (SAR) is situated at the Pearl River Delta on the southeast coast of mainland China. With urban development accelerating at great rate in a quite restricted area, Macao still has rich fauna, within which the insect diversity is surprisingly high.

**New information:**

In this study, we systematically sorted out major references items of manuals or handbooks, monographs, articles, dissertations, official websites and other publicly available information sources about the insects recorded in Macao and, thus, generated a checklist of 15 orders, 166 families, 868 genera, 1,339 species and 118 subspecies. During this process, the preliminarily summarised list was re-examined to eliminate synonyms and invalid species, based on many more extensive literature reviews. Besides, spelling errors of scientific names, authors and years were corrected. Meanwhile, the catalogue revealed a different composition pattern of species diversity between orders from those of the world and China. Even based on the most conservative estimates, the number of insect species in Macao should not be lower than 3,340 species, which hints at the necessity of deeper investigations with adequate collecting in the future to achieve more comprehensive recognition and understanding of Macao’s insect biodiversity.

## Introduction

Insects are the most diverse organism groups with dominant number of species on Earth, involving vast ecological niches. They play crucial roles in various ecosystems, including pollination, seed dispersal, decomposition etc. Currently, there are roughly one million known insect species worldwide (Grimaldi and Engel 2005), accounting for around half of all known cellular organisms and about two–thirds of all animal species. Their common ancestor originated in the Middle Ordovician (Wang et al. 2023). Based on recent phylogenetic studies, extant insect orders are currently classified into 27–28 orders (McKenna and Farrell 2010, Ishiwata et al. 2011, Misof et al. 2014, Peters et al. 2014, Wang et al. 2016, Johnson et al. 2018, Wipfler et al. 2019,Wang et al. 2023). The six largest orders are Coleoptera (386,755 species), Diptera (157,971 species), Lepidoptera (157,761 species), Hymenoptera (154,067 species), Hemiptera (107,401 species) and Orthoptera (26,107 species) (Foottit and Adler 2017). Based on the existing catalogues, monographs, articles etc., China had close to 100,000 insect species until 2006 and the six largest orders are Coleoptera (23,643 species), Lepidoptera (17,786 species), Diptera (15,404 species), Hymenoptera (12,517 species), Hemiptera (11,973 species) and Orthoptera (2,716 species), respectively (Shen 2015).

Macao Special Administrative Region (SAR) is located in the subtropical region of the southern Pearl River Delta, at the intersection of the Chinese mainland and the South China Sea. It falls within the South China entomogeographic region (Shen 2015).It has an area of 33.3 km^2^ and one of the most densely populated places on the planet. The climate of Macao is classified as subtropical maritime monsoon with an average annual temperature of 22.7℃, a relative humidity of around 82% and a mean annual precipitation of approximately 2030.8 mm (DSEC 2023). The hot and humid climate, combined with its geographical location where water and land meet, provide favourable natural conditions for plant growth and animal foraging in Macao. Despite its small area and highly urbanisation, Macao has a rich insect diversity due to its diverse habitats, including wetlands, shrublands, forests and urban areas (Li et al. 2018, Li 2023). The *Manual de insectsos de Macao* authored by Pun and Batalha (1997) is the earliest integrative monograph on the insect fauna of Macao. The authors spent over a decade conducting surveys and compiling a checklist of the insect species in Macao. According to the taxonomy system at that time, it included a total of nine orders, 99 families and 450 species. Since 1996, different scholars have conducted different degrees of research on insect fauna of Macao based on morphology (Table 1), which totally contains 1,269 species names then (not 1,269 species) belonging to eight orders. Additionally, although such research provided a general understanding of the insect fauna in Macao, there still existed numerous fragmented species records, which consisted of considerably valuable information as a total and could not be neglected. Therefore, it is necessary to update the species catalogue of Macao’s insect fauna. Apart from that, we hope to raise awareness amongst more experts about diversity patterns of insect in cities like Macao.

## Materials and methods

The geographical scope of this work is Macao, which has an area of 33.3 km^2^ and population density as 20,300 inhabitants per km^2^ (DSEC 2023). This catalogue is primarily based on the publicly available literature and online resources. It does not include any information of museums and private collections other than those already present in literature, websites and other publicly available information sources.The specific steps for the compilation of this information are as follows:

1. Gathering all manuals or handbooks, monographs, articles, dissertations, official websites and other publicly available information sources about insects recorded in Macao, preliminarily summarising a list of species mentioned in these sources;
2. As usual, eliminated synonyms and unverifiable species names and supplemented missing information of the author and years in scientific names;
3. Discussing the pattern of insect diversity in Macao, based on the newly-compiled catalogue of insect species. The order names were arranged according to the account of species and the families, genera and species were arranged alphabetically.

## Checklists

### Checklist of species from Macao

#### Lepidoptera

AB6C113C-00EB-5421-BD69-CEBCD9AFA2B0

#### Arctiidae

9A24C769-CDB9-59FF-8826-01FA3BAC83C3

#### Amata

Fabricius, 1807

004F1529-8BC3-5EA1-B916-BAA273C3F20D

#### Amata atkinsoni

(Moore, 1878)

02953787-4016-59F2-872A-2B9468BD2DEF

##### Notes

Li (2023)

#### Amata germana

(Felder & Felder, 1862)

358F157A-4715-5729-B28C-5128DD1B484E

#### Amata germana germana

(Felder & Felder, 1862)

8249E70F-1A22-5F6C-9874-CA82FFC8D885

##### Notes

Easton and Pun (1996)

#### Amata grotei

(Moore, 1871)

1504AF92-471E-5CF1-8FCA-86796B50681B

##### Notes

Easton and Pun (1996)

#### Amata polymita

(Sparrman, 1769)

33093098-1B89-503A-AF0C-9102ADEB5B76

##### Notes

MBD (2022)

#### Amata sperbius

(Fabricius, 1787)

798B67CF-7ADC-51D2-98A6-E20280AEA5D1

##### Notes

Easton (1992)

#### Amerila

Walker, 1855

2B3BAB45-F74B-56EB-A76E-12DAD2A96F48

#### Amerila astreus

(Drury, 1773)

CF22EEBA-E85E-5B35-8027-4C075D2BC3CD

##### Notes

Easton and Pun (1996)

#### Argina

Hübner, 1819

076E4CF7-F637-51BF-829A-ABBE24E9F425

#### Argina astrea

(Drury, 1773)

8ECAC5E9-A96B-53F7-84F2-B205465F7899

##### Notes

Easton and Pun (1996)

#### Baroa

Moore, 1878

FF252F96-DF1C-51F7-821B-060D268C58EE

#### Baroa vatala

Swinhoe, 1894

5574D12F-137E-5FD6-96D4-1AEEAAB24CBF

##### Notes

Li (2023)

#### Creatonotos

Hübner, 1819

96823F80-781E-51EA-BD74-1FEB60E8E692

#### Creatonotos gangis

(Linnaeus, 1763)

7501C702-998D-54CE-B6E7-4BC8DCB639A0

##### Notes

Easton and Pun (1996)

#### Creatonotos transiens

(Walker, 1855)

A8D1A235-BDA5-5682-ACB9-B87D267DF45E

##### Notes

Easton and Pun (1996)

#### Cyana

Walker, 1854

69C52EBB-7721-5689-A5C4-02576110724B

#### Cyana alborosea

(Walker, 1864)

77CCDAE2-8D54-55BC-A23A-ED68327785D3

##### Notes

Pun and Batalha (1997)

#### Eilema

Hübner, 1819

DA3DE92D-7BD1-5B77-A66B-7A335A4584E4

#### Eilema hunanica

(Daniel, 1954)

4B17C01D-226E-52BA-BE8E-0BF8EF3ACF4C

##### Notes

Pun and Batalha (1997)

#### Eilema vicaria

(Walker, 1854)

78808B61-5CE0-5A9A-8720-62DDB9BA9E64

##### Notes

Pun and Batalha (1997)

#### Macrobrochis

Herrich-Schäffer, 1855

959E8C18-C573-5BB9-B7C5-7DF34C1B92CC

#### Macrobrochis gigas

(Walker, 1854)

145B7301-EBC4-5BA5-B681-D2C341B36961

##### Notes

Pun and Batalha (1997)

#### Miltochrista

Hübner, 1819

17D50064-9EB7-5EE7-B408-E3AB067DF17C

#### Miltochrista striata

(Bremer & Grey, 1853)

D35DF50A-DF37-5191-BF34-F3DF47A08409

##### Notes

Pun and Batalha (1997)

#### Nyctemera

Hübner, 1820

76EBBD71-5A06-5FBD-B271-F65D53E3140D

#### Nyctemera adversata

(Schaller, 1788)

FD339CE6-B80D-5483-BF71-A051A776858A

##### Notes

Pun and Batalha (1997)

#### Nyctemera lacticinia

(Cramer, 1777)

8F33312A-B650-5084-AD09-BD57BA153937

##### Notes

Li (2023)

#### Nyctemera tripunctaria

(Linnaeus, 1758)

31CDF5D4-1CEE-5BD5-BAC7-EA935CE6EED6

##### Notes

Pun and Batalha (1997)

#### Spilarctia

Butler, 1875

6EA9F7EE-8AE2-5492-9404-53E8D64FDE74

#### Spilarctia bisecta

(Leech, 1888)

C2EAD4E4-8047-5FBE-BEF7-2E2F777A6AFC

##### Notes

Pun and Batalha (1997)

#### Spilarctia robusta

(Leech, 1899)

53A3AAD5-AD11-5926-B9D6-6FA54646F955

##### Notes

Pun and Batalha (1997)

#### Syntomoides

Hampson, 1893

991DDAFF-523C-5165-A355-C29F4ADDACD3

#### Syntomoides imaon

(Cramer, 1780)

4E6E5F8B-3D9A-5CA5-AD8B-FDB4A1CBE48F

##### Notes

Pun and Batalha (1997)

#### Utetheisa

Hübner, 1819

559ED2D8-74EB-5E4C-9243-B08C956A54EA

#### Utetheisa lotrix

(Cramer, 1779)

6EDA38F8-CF8A-5933-A119-EB8BB564D0A8

##### Notes

Li (2023)

#### Attevidae

1AB897D8-5204-5F5F-AAC1-64D86B26A7C2

#### Atteva

Walker, 1854

4A7B7477-53B7-5351-9768-72DBC1CB970A

#### Atteva fabriciella

(Swederus, 1787)

5C163C45-F807-5F26-AC8B-9F1EE623D62A

##### Notes

Pun and Batalha (1997)

#### Bombycidae

25401CFD-2065-56E0-AA5D-515E8CC42E15

#### Ernolatia

Walker, 1862

A60C76A0-73EE-5233-83B4-31C742F36786

#### Ernolatia moorei

(Hutton, 1865)

4408F106-8F8E-5251-AC35-43F6AE1D53DF

##### Notes

Pun and Batalha (1997)

#### Ocinara

Walker, 1856

24AE7E54-5E9F-51F2-A2AF-94FBCB5941F2

#### Ocinara albicollis

(Walker, 1862)

58E0355B-FFC6-5DAC-80B2-55ADA2ACDA5F

##### Notes

Pun and Batalha (1997)

#### Ocinara nitida

Chu & Wang, 1993

9DC7E5EA-FC99-54B9-BA78-35B97E6B8008

##### Notes

DSPA (2022)

#### Cossidae

15C160AA-D1C9-5940-A2AF-5CA3307D7316

#### Zeuzera

Latreille, 1804

DD03BF3A-887F-5705-A450-6E3B21D41020

#### Zeuzera coffeae

Nietner, 1861

CD3FC915-2D99-5E84-88EB-3C12C3632C5E

##### Notes

Pun and Batalha (1997)

#### Crambidae

CF033895-739B-5F9C-86B5-5050D397744F

#### Aethaloessa

Lederer, 1863

D23B678D-4C67-5064-BC5E-562935A7450D

#### Aethaloessa calidalis

(Guenee, 1854)

D8564CA5-7500-5B2A-B1F4-5E5AEFFBA416

##### Notes

Li (2023)

#### Aethaloessa floridalis

(Zeller, 1852)

84CBF9C1-0EAE-5DE8-BADF-2785899A1F36

##### Notes

DSPA (2022)

#### Agathodes

Guenée, 1854

1D1931D7-0802-5697-B606-90F6A731818C

#### Agathodes ostentalis

(Geyer, 1837)

BD92680A-3B32-5A5B-B2E3-5161FE612839

##### Notes

Pun and Batalha (1997)

#### Anania

Hübner, 1823

56520C08-0DBB-5AD6-95A3-780BEC934AF8

#### Anania coclesalis

(Walker, 1859)

5E53510B-3E9A-5FD4-AC58-0CD21D197C09

##### Notes

Pun and Batalha (1997)

#### Ategumia

Amsel, 1956

32907AA2-754D-5BF7-8EDE-14CBBE181E50

#### Ategumia adipalis

(Lederer, 1863)

FEF56B82-82E8-536C-AFC3-3C6C3EBD256C

##### Notes

Li (2023)

#### Ancylolomia

Hübner, 1825

A3064440-7E60-5DBF-8A9B-132EC71FD834

#### Ancylolomia japonica

Zeller, 1877

3E3EF6A5-9A5A-55EB-AF19-C5BE6D5D2F85

##### Notes

Pun and Batalha (1997)

#### Bocchoris

Hübner, 1818

65DC238D-5CEA-525D-AE8F-5EE3791D03C4

#### Bocchoris inspersalis

(Zeller, 1852)

906ACB65-698E-5880-8E43-E7E3A578043A

##### Notes

Pun and Batalha (1997)

#### Botyodes

Guenée, 1854

06158F15-3021-5475-B02F-F03123FB7E22

#### Botyodes asialis

Guenée, 1854

BA407DAB-6521-5BCE-A7A9-414B31267EB2

##### Notes

DSPA (2022)

#### Botyodes caldusalis

Walker, 1859

5D37E94F-D2B0-517E-9D5D-8E7C6E70E21E

##### Notes

DSPA (2022)

#### Botyodes diniasalis

(Walker, 1859)

FDE90F4E-F922-562F-9D4B-2D6700C0EE1F

##### Notes

Pun and Batalha (1997)

#### Botyodes principalis

Leech, 1889

4F7BB122-DF2D-55C1-8C22-D7F06AA588D6

##### Notes

Li (2023)

#### Bradina

Lederer, 1863

EA57DE7D-16F1-59CD-B54D-F0B859F5E018

#### Bradina atopalis

(Walker, 1858)

5C04976A-6ABF-5907-B74B-3041BC03F6FB

##### Notes

Li (2023)

#### Camptomastix

Warren, 1892

4A7161A3-AD7B-5AEF-8EC2-2396A33C4138

#### Camptomastix hisbonalis

(Walker, 1859)

E2478ACC-6519-5A5F-882E-EFB743EB8A28

##### Notes

Li (2023)

#### Chilo

Zincken, 1817

D5A6B0BB-1DBF-5187-AA52-E1527C60C4D5

#### Chilo suppressalis

(Walker, 1863)

C18788BC-86BD-5ED7-9B6F-C52C157CD2F5

##### Notes

Li (2023)

#### Cirrhochrista

Lederer, 1863

669CC1F5-78AB-545F-B44D-1AF4AD1C5C9E

#### Cirrhochrista brizoalis

(Walker, 1859)

0380C0CF-3D41-564C-8BFE-D0E89B36BF89

##### Notes

Pun and Batalha (1997)

#### Cirrhochrista kosemponialis

Strand, 1919

5B4D1D47-14D9-51AF-9435-9D54A97FE1DE

##### Notes

Pun and Batalha (1997)

#### Cnaphalocrocis

Lederer, 1863

1B55B19F-453D-5923-8B26-F89A1DC438C2

#### Cnaphalocrocis limbalis

(Wileman, 1911)

FF7AEDD3-00C1-578A-9FBB-E25343006FE9

##### Notes

Li (2023)

#### Cnaphalocrocis medinalis

(Guenée, 1854)

3437C294-436A-5A60-BAAC-721E65CCE312

##### Notes

Pun and Batalha (1997)

#### Cotachena

Moore, 1885

EAE5358D-1C2D-5BED-B2BA-C204314CD494

#### Cotachena histricalis

(Walker, 1859)

904E942F-81B4-5C87-B9B4-1852B1D43C30

##### Notes

Pun and Batalha (1997)

#### Conogethes

Meyrick, 1884

D43CCB79-DEC3-54F1-B3CC-D5EA41EFF742

#### Conogethes punctiferalis

(Guenée, 1854)

26FF0C7B-7019-51E7-9CC3-2CDCEA0D588F

##### Notes

Li (2023)

#### Crypsiptya

Meyrick, 1934

69B9DE5F-6B1E-5745-93F0-6C566EC5B081

#### Crypsiptya coclesalis

(Walker, 1859)

2BB9487D-0E64-536B-8391-68F0F9678E7D

##### Notes

Pun and Batalha (1997)

#### Diaphania

Hübner, 1818

7021115A-76D3-596F-A150-BCD3B3CA4FBE

#### Diaphania annulata

(Fabricius, 1794)

1704EA5E-0697-5333-89AC-A7D64E9ADD7F

##### Notes

Pun and Batalha (1997)

#### Diaphania bicolor

(Swainson, 1821)

336BC297-A6C6-5EC5-BEE6-BAAABB563034

##### Notes

Pun and Batalha (1997)

#### Diaphania bivitralis

Guenée, 1854

AE645FD3-C0F4-5CB7-8481-F5D3DCA73DC3

##### Notes

Pun and Batalha (1997)

#### Diaphania indica

(Saunders, 1851)

35E502A2-0F95-55E0-887A-CB2D31DE8BD5

##### Notes

Pun and Batalha (1997)

#### Diasemia

Hübner, 1825

85D9A6DA-6F03-5FA9-807D-8A3396BC8954

#### Diasemia accalis

(Walker, 1859)

C5E410A1-A88C-5B3F-9EC5-10CF4AAD8CF5

##### Notes

Li (2023)

#### Epipagis

Hübner, 1825

DB6D4C91-DD9B-5A72-BB92-20C12388FEB1

#### Epipagis cancellalis

(Zeller, 1852)

2E93793C-7D51-54C1-B2E6-8FDC1E872F38

##### Notes

Easton and Pun (1996)

#### Euclasta

Lederer, 1855

08E7A287-0E09-5FDF-91C9-3B1354A8977B

#### Euclasta defamatalis

Walker, 1859

3E8BAB7C-3394-5328-8881-2279D7A29DE5

##### Notes

Pun and Batalha (1997)

#### Euclasta vitralis

Maes, 1997

6AC0CF45-607D-563B-A47E-55EC81BA7FCF

##### Notes

Li (2023)

#### Eurrhyparodes

Snellen, 1880

B25D3944-4AB8-5C8A-A463-04EC7B7F06E3

#### Eurrhyparodes bracteolalis

(Zeller, 1852)

655E5BF4-6ED4-55C1-8738-725BD9760D32

##### Notes

Li (2023)

#### Glyphodes

Guenée, 1854

FF6EB4CF-297D-566E-B8AC-66CF42E8A676

#### Glyphodes bicolor

(Swainson, 1821)

83C89493-F0AD-5DCF-80EC-919B706269F4

##### Notes

Li (2023)

#### Glyphodes biνitralis

(Guenée, 1854)

0ACD8300-3B36-5833-AA1B-7D73C2E8487C

##### Notes

Li (2023)

#### Glyphodes caesalis

Walker, 1859

6C159325-9EA6-55BE-BFA1-F740F03963ED

##### Notes

Li (2023)

#### Glyphodes canthusalis

(Walker, 1859)

41D8324A-5B31-5EB1-9819-C942DE1FA230

##### Notes

Pun and Batalha (1997)

#### Glyphodes onychinalis

(Guenée, 1854)

3F7701DD-5E3A-56B9-9913-D7BA483C6EFA

##### Notes

Li (2023)

#### Glyphodes strialis

(Wang, 1963)

92AB1C42-7A2F-5C01-804A-E63930A81AE2

##### Notes

Li (2023)

#### Haritalodes

Warren, 1890

906FB57F-CE42-5F68-A646-D41EFE2532A5

#### Haritalodes derogate

(Fabricius, 1775)

570EED75-D45C-519D-BBC5-FBB72E93DBE8

##### Notes

Pun and Batalha (1997)

#### Hemiscopis

Warren, 1890

B2F841D6-4E06-5CFE-B96F-A8AAC5405DA7

#### Hemiscopis sanguinea

Bänziger, 1987

E2ACC426-6373-5378-A34F-FE2484B811A9

##### Notes

Li (2023)

#### Heortia

Lederer, 1863

3227BC44-A4E5-5DF9-AC20-057A476260BC

#### Heortia vitessoides

(Moore, 1885)

027ADA90-3DAB-5B41-9BA8-7DF29EB5CCCA

##### Notes

Li (2023)

#### Herpetogramma

Lederer, 1863

2A9CDF6F-9392-54BE-9C11-F502B97A2356

#### Herpetogramma basalis

(Walker, 1866)

9F819093-1F33-5388-BF02-68E4A1E94592

##### Notes

Li (2023)

#### Herpetogramma cynaralis

(Walker, 1859)

FF47C908-A9D3-5AF1-B262-FA90AA85903B

##### Notes

Li (2023)

#### Herpetogramma licarsisalis

(Walker, 1859)

3CEB80AD-E6A8-5A40-BFE7-0761C5C2E3FD

##### Notes

Li (2023)

#### Herpetogramma luctuosalis

(Guenée, 1854)

0F9C8C51-236F-5BD1-8ACF-ECD4833348ED

##### Notes

Pun and Batalha (1997)

#### Herpetogramma rudis

Warren, 1892

02C9C0C3-0D63-549A-BB42-BA458B14EB66

##### Notes

DSPA (2022)

#### Herpetogramma submarginalis

(Swinhoe, 1901)

602CDCE6-432F-55B1-80E8-5BD3CADE50E8

##### Notes

Li (2023)

#### Hyalobathra

Meyrick, 1885

27AD82DA-8BB1-52B5-9831-0BDE6D93AA75

#### Hyalobathra coenostolalis

(Snellen, 1890)

0DE4CC48-E178-5D37-A090-D7EB47F09CC1

##### Notes

Li (2023)

#### Hyalobathra opheltesalis

(Walker, 1859)

CBFED2C5-C12B-566E-9969-08153CBF9C11

##### Notes

Li (2023)

#### Hydriris

Meyrick, 1885

6FA5C0E5-C81A-58FF-BF54-7ECDB502ADD4

#### Hydriris ornatalis

(Duponchel, 1832)

6236139D-5B1A-5B21-9458-FA41E9494A11

##### Notes

Li (2023)

#### Hymenia

Hübner, 1825

CD9CB741-24A1-5C70-B8BC-0660EFF5DA15

#### Hymenia perspectalis

(Hübner, 1796)

93AED64F-31EE-5DC9-B631-96FD906DE4F1

##### Notes

Pun and Batalha (1997)

#### Hymenia recurvalis

(Fabricius, 1775)

503C3072-363E-5A65-8DDF-50C00BB898B4

##### Notes

Pun and Batalha (1997)

#### Ischnurges

Lederer, 1863

C4ADFD10-E42E-5550-9403-E82DD25C4F8A

#### Ischnurges gratiosalis

(Walker, 1859)

E6FAB863-29AA-5439-8F64-C1D33796E361

##### Notes

Li (2023)

#### Isocentris

Meyrick, 1887

21BD0E20-9458-5417-B58B-608B5D35E0C5

#### Isocentris aequalis

(Lederer, 1863)

D71B186D-F93A-5E60-A4FD-159AFF2F9148

##### Notes

Li (2023)

#### Lamprosema

Hübner, 1823

8FE4E263-8092-5398-BE45-7FF3980B5E17

#### Lamprosema tampiusalis

Walker, 1859

2BBA8978-54E7-5CBF-82F6-09026993B7C4

##### Notes

DSPA (2022)

#### Mabra

Moore, 1885

6847F450-1CE1-5AF1-8AD5-D2CBDA5ECBA7

#### Mabra charonialis

(Walker, 1859)

302F1169-A8D8-518C-AD1E-0BE75764A55E

##### Notes

Li (2023)

#### Mabra eryxalis

(Walker, 1859)

D25398CE-0D34-5BB6-9FBC-408C49FC9046

##### Notes

Li (2023)

#### Maruca

Walker, 1859

219FDC53-14F8-51E2-B870-7C1F2139354D

#### Maruca testulalis

(Geyer, 1832)

7838FD0E-72F2-552B-8CD2-31CEBF23C890

##### Notes

Pun and Batalha (1997)

#### Maruca vitrata

(Fabricius, 1787)

E1EAE30A-C90A-59AD-9C70-5E744D3A6EAD

##### Notes

Easton and Pun (1996)

#### Nacoleia

Walker, 1859

2285B413-7A1C-54C2-ACFF-4371C850DF45

#### Nacoleia charesalis

(Walker, 1859)

D7496174-90F0-5040-89B8-2E846A9C9819

##### Notes

Li (2023)

#### Nacoleia commixta

(Butler, 1879)

86A8EBE9-F744-53E0-A7BC-D7EAF00C0F70

##### Notes

Li (2023)

#### Nacoleia tampiusalis

(Walker, 1859)

59B83B02-AC44-5347-AB77-66FC883FD943

##### Notes

Li (2023)

#### Nausinoe

Hübner, 1825

E97FC351-E720-5B82-81A4-D2660E7486D8

#### Nausinoe geometralis

(Guenee, 1854)

716D1222-1350-5790-BC1E-C35B62725B5A

##### Notes

Easton and Pun (1996)

#### Nausinoe perspectata

(Fabricius, 1775)

3637E27D-4911-5CD5-BF57-0AE85013F81F

##### Notes

Li (2023)

#### Nephelobotys

Munroe & Mutuura, 1970

F733400C-E369-5FAC-A37A-32A72AA83FA5

#### Nephelobotys habisalis

(Walker, 1859)

1FC37FC8-7AE9-5288-960C-5F2FFD57893E

##### Notes

Li (2023)

#### Nomophila

Hübner, 1825

7BAAD3E8-B61C-5328-8F9C-8FE212D57FFA

#### Nomophila nocteulla

(Denis & Schiffermlüller, 1775)

DD8984C4-6207-532E-883E-CA9A13AC912F

##### Notes

Li (2023)

#### Nosophora

Lederer, 1863

8FF47E7C-0452-5A25-B127-3BFFF2AA715B

#### Nosophora semitritalis

(Lederer, 1863)

EA6534D8-D4A4-5200-841D-9223542B82C0

##### Notes

Li (2023)

#### Notarcha

Meyrick, 1884

41A0502E-6A35-58E0-A1F4-590D1B74AA58

#### Notarcha quatemalis

(Zeller, 1852)

B1C24B97-E69C-5672-A6E2-4A303973C140

##### Notes

Li (2023)

#### Nymphula

Schrank, 1802

EAF569C8-7E47-53C4-B97A-BD97B6CF540D

#### Nymphula fluctuosalis

Zeller, 1852

F5EAFD6C-9BF4-500E-9DE2-6345C3513DCC

##### Notes

Pun and Batalha (1997)

#### Omiodes

Guenée, 1854

632209D8-C76C-5602-B387-7075439FE37F

#### Omiodes indicata

(Fabricius, 1775)

BDF73B27-C676-5472-92DD-E6990103D5D7

##### Notes

DSPA (2022)

#### Omphisa

Moore, 1886

1BFA3383-E4AD-50AD-8F70-9F1FF200799D

#### Omphisa anastomosalis

Guenée, 1854

4DEB7EA0-5B12-50B8-89BD-4CD9BD7E5A2E

##### Notes

DSPA (2022)

#### Parapoynx

Hübner, 1825

28FCA5D4-3338-5F5D-863A-3FBB70DB49C2

#### Parapoynx diminutalis

Snellen, 1880

FB007C47-A63D-57C2-8169-EE5677E5E4AD

##### Notes

Li (2023)

#### Parapoynx fluctuosalis

(Zeller, 1852)

2B98D047-8C1E-5D89-A279-F0538A013300

##### Notes

Li (2023)

#### Palpita

Hübner, 1808

C3067307-9604-58CA-8C73-68E1B98C4141

#### Palpita munroei

Inoue, 1996

474E428B-E491-5FFD-AB91-17B83477304C

##### Notes

Li (2023)

#### Palpita nigropunctalis

(Bremer, 1864)

E4E5CC4F-FC21-592E-A7A6-1CEB8A92F4D0

##### Notes

Li (2023)

#### Parotis

Hübner, 1831

E3365EE8-6CA1-5D47-9DF8-331771BD11AA

#### Parotis angustalis

(Snellen, 1875)

23044BEB-D1E7-597F-B984-987A9C9B360A

##### Notes

Li (2023)

#### Parotis suralis

(Lederer, 1863)

8F8BF01D-BC32-5E57-8653-40595E9F4E85

##### Notes

Li (2023)

#### Patania

Moore, 1888

D18B1DE6-6A60-54D1-9C36-008B0958882F

#### Patania chlorophanta

(Butler, 1878)

F37C06FF-ACDE-5986-BA0D-0FB9D664CB6F

##### Notes

Li (2023)

#### Pleuroptya

Meyrick, 1890

6F770C31-0AF5-5254-921F-FDC1762FEA85

#### Pleuroptya balteata

(Fabricius, 1798)

A7168D92-9232-5EED-8F0D-4CC9055015E8

##### Notes

Li (2023)

#### Poliobotys

Shaffer & Munroe, 2007

A4CA34C7-B0B2-5BBB-868A-752C0F595B9E

#### Poliobotys ablactalis

(Walker, 1859)

36B781CD-FA33-575D-94F0-E162611752B3

##### Notes

Li (2023)

#### Prophantis

Warren, 1896

9D9A58C0-705E-5932-B6FE-FF5FE1CB8B1C

#### Prophantis adusta

Inoue, 1986

B44C026C-FFFF-5A6C-BE56-8D795A30212C

##### Notes

Li (2023)

#### Pseudocatharylla

Bleszynski, 1961

4F6CEAB9-A98C-56CC-BA1B-A91FD4E09896

#### Pseudocatharylla duplicella

(Hampson, 1895)

3F15DB50-C886-54E5-B28A-74334B22310B

##### Notes

Mo et al. (2006)

#### Pseudocatharylla simplex

(Zeller, 1877)

249257DE-4A98-5D80-A45F-CA78DAB5D268

##### Notes

DSPA (2022)

#### Pycnarmon

Lederer, 1863

23EEBB23-0BC0-52B2-8860-70CD2464E1C0

#### Pycnarmon cribrata

(Fabricius, 1794)

8C60BE99-AF19-5B01-B913-6EC01FA69A06

##### Notes

Li (2023)

#### Rehimena

Lederer, 1863

8D0FB917-40F0-5759-8C2A-602C928B73A7

#### Rehimena phrynealis

(Walker, 1859)

3CAF2174-319E-579D-A0B8-B02288008F53

##### Notes

Li (2023)

#### Rehimena surusalis

(Walker, 1859)

31D5899D-5303-566A-8570-90959CADE6E4

##### Notes

Li (2023)

#### Sameodes

Snellen, 1880

164D4182-23DC-5C9D-AEB0-4B00254279D3

#### Sameodes cancellalis

(Zeller, 1852)

BBA6B6B5-A129-5C14-8AC5-02D79DD43FEA

##### Notes

Pun and Batalha (1997)

#### Schoenobius

Duponchel, 1836

2873371E-477D-55E0-9A52-8E38CE5185A1

#### Schoenobius gigantellum

(Denis & Schiffermüller, 1775)

F6EDE4B8-D273-5DF6-8FA2-4906F200BB5A

##### Notes

DSPA (2022)

#### Scirpophaga

Treitschke, 1832

7CB30513-1655-53C9-96CC-EC33CAC192C7

#### Scirpophaga excerptalis

(Walker, 1863)

7AE4B731-5830-5CA4-A79A-8891CBD50B31

##### Notes

Li (2023)

#### Scirpophaga incertulas

(Walker, 1863)

E0CCEB99-0DA8-5730-9AC7-69D8C2095B59

##### Notes

Li (2023)

#### Sclerocona

Meyrick, 1890

C8BACA6B-3EB6-5FF1-BE40-EF90161371CA

#### Sclerocona acutellus

(Eversmann, 1842)

B4B1E2E0-0F3C-56E7-AB1A-06D2842C4EE9

##### Notes

Li (2023)

#### Spoladea

Guenée, 1854

BD57CF4B-B5D8-566F-999F-7B5B873F515D

#### Spoladea recurvalis

(Fabricius, 1775)

1C9CA632-896C-5096-9D91-993A7F4893EA

##### Notes

Li (2023)

#### Syllepte

Hübner, 1823

245EE73F-249A-5923-B78B-97D5D87E3EFF

#### Syllepte derogata

(Fabricius, 1775)

83A17F81-FF37-5D8B-9ECC-E42C5F2069D5

##### Notes

Pun and Batalha (1997)

#### Talanga

Moore, 1885

79C2B7B8-285E-5EEB-8BC6-FC220B26465D

#### Talanga sexpunctalis

(Moore, 1877)

A933BA26-F916-5A63-9E54-F6E5EE655691

##### Notes

Li (2023)

#### Tatobotys

Butler, 1880

956953CB-7529-526A-AB4D-19A14408D0D4

#### Tatobotys biannulalis

(Walker, 1866)

BDA8BC7C-CDEA-59DE-9034-E08DA52FAFBB

##### Notes

Li (2023)

#### Thliptoceras

Warren, 1890

19AD8DB8-B291-576D-87EB-4FAF3CC2DF68

#### Thliptoceras formosanum

Munroe & Mutuura, 1968

D8295D70-3361-5793-8C41-5DEBAEADF7F9

##### Notes

Li (2023)

#### Trichophrsetis

Pagenstecher, 1909

DB60FFA8-4558-5F30-8B04-B89F2C576506

#### Trichophrsetis cretacea

(Butler, 1879)

146B61CE-33BF-58D4-B526-E1E95428567B

##### Notes

Li (2023)

#### Tryporyza

Common, 1960

44D3B589-5382-5FA9-8B69-90D1B5CE31B2

#### Tryporyza nivella

(Fabricius, 1794)

09E3F524-611C-5938-8ED2-DE323CBBEA81

##### Notes

Pun and Batalha (1997)

#### Tyspanodes

Warren, 1891

553E5E6B-CE92-5B49-ABE7-E34E8EB51641

#### Tyspanodes linealis

(Moore, 1867)

ABAE6261-2673-56F8-80BA-E2ADA65E816B

##### Notes

Li (2023)

#### Udea

Guenée, 1845

C582A9E3-C2A7-5535-91CC-D398274A1AD6

#### Udea ferrugalis

(Hübner, 1796)

5FB08B5F-5821-5006-A4CC-1604D853284F

##### Notes

DSPA (2022)

#### Drepanidae

1E4DC2A4-2D97-530C-BBA4-68FCAC65B3ED

#### Drapetodes

Guenée, 1858

94A1E41A-CCA1-5F7F-917F-9BE18FC62A61

#### Drapetodes mitaria

Guenee, 1857

7D95FC6F-155F-51DC-BDCE-BA6C50BC51A8

##### Notes

Li (2023)

#### Oreta

Walker, 1855

941C003D-ED0B-580A-9281-0C36D948BDC8

#### Oreta hoenei

Watson, 1967

6467E81F-6F65-55BD-9E16-1835E4077DB3

##### Notes

Li (2023)

#### Elachistidae

F388F28D-84A7-5916-BC1B-93E58CEB577F

#### Ethmia

Hübner, 1819

26066C75-EAC3-5663-B584-8D9B899DC53F

#### Ethmia lineatonotella

(Moore, 1867)

D679422B-0E6F-5A1F-B49F-F610E46D9DEE

##### Notes

Li (2023)

#### Geometridae

FC344C14-BB1D-5760-9D6F-7F55D6E3EFB1

#### Abraxas

Leach, 1815

F6F2382B-FC8B-5E7B-85B1-88058EF3D50F

#### Abraxas nanlingensis

Inoue, 2005

6D22C6FC-57C7-5DFB-BEA3-02D15F0B66C5

##### Notes

Li (2023)

#### Abraxas neomartania

Inoue, 1970

62B5CB3C-1083-541A-A8C1-4DB436357167

##### Notes

Li (2023)

#### Abraxas plumbeata

Cockerell, 1906

9F40DAFE-CC26-5F4D-B55D-5E50E79747F3

##### Notes

Pun and Batalha (1997)

#### Agathia

Guenée, 1858

5557EFDF-6DC0-5644-8E26-772A01316DFF

#### Agathia lycaenaria

(Kollar, 1844)

18CCE650-7B59-536E-B5D4-3D83AAD603BB

##### Notes

Pun and Batalha (1997)

#### Antitrygodes

Warren, 1895

F623CAA6-1FF1-5FE2-81C3-B88873D49FAF

#### Antitrygodes divisaria

(Walker, 1861)

D020D3CD-B7E9-5B0F-B372-95C84834C11F

#### Antitrygodes divisaria perturbatus

Prout, 1914

1A9F7B91-BC98-54D0-B3E1-1E370D413F4E

##### Notes

Li (2023)

#### Ascotis

Hübner, 1825

51B924D2-5949-58D0-B6C6-3F5F7B7E8BB8

#### Ascotis selenaria

(Denis & Schiffermüller, 1775)

AF1C8804-8071-528F-9B55-73D522A997A1

##### Notes

Pun and Batalha (1997)

#### Biston

Leach, 1815

129CE11B-4A19-5184-859F-99AF4A56ADDB

#### Biston marginata

Shiraki, 1913

176E3732-6C47-5908-841A-B324262E750A

##### Notes

DSPA (2022)

#### Biston suppressaria

(Guenée, 1858)

A2874490-38AD-58F5-A212-5BD56598AA01

##### Notes

Pun and Batalha (1997)

#### Chiasmia

Hübner, 1823

99304274-4390-5916-9E47-E8557FB318FA

#### Chiasmia emersaria

(Walker, 1861)

7AEA981A-C9F9-5DA0-969F-8854B39D32EF

##### Notes

Pun and Batalha (1997)

#### Chiasmia pluviata

(Fabricius, 1798)

E2399CFC-33DB-55C1-96C9-037BBC87A3D2

##### Notes

Li (2023)

#### Cleora

Curtis, 1825

3AA22E51-811B-5FE2-9F78-C24300F84E4A

#### Cleora fraterna

(Moore, 1888)

13F376B2-15B6-588E-83D8-9E1756359BF9

##### Notes

Li (2023)

#### Dysphania

Hübner, 1819

B73904FB-F3A6-5EF8-A6DA-F15991E7782F

#### Dysphania militaris

(Linnaeus, 1758)

49C22FAF-FF5D-598C-B482-3DBAEAAD33B7

##### Notes

Pun and Batalha (1997)

#### Dasyboarmia

Prout, 1928

8735214E-78A4-5582-A272-EF13FC62FA76

#### Dasyboarmia subpilosa

(Warren, 1894)

202865B4-CB36-5B4E-9A61-AA1BA6E9C2A9

##### Notes

DSPA (2022)

#### Ectropis

Hübner, 1825

ED215D5C-6285-52C9-8928-769A524CD542

#### Ectropis crepuscularia

(Denis & Schiffermiiller, 1775)

5606B5C0-E9A5-5694-AB6E-0F3008FBCB6E

##### Notes

Li (2023)

#### Eumelea

Duncan & Westwood, 1841

1EFB8453-85EB-565A-9120-9A61BDF20C03

#### Eumelea biflavata

Warren, 1896

DB3BA961-81FF-5AB4-A39B-7A5F0CDE212C

##### Notes

Pun and Batalha (1997)

#### Eumelea ludovicata

Guenée, 1857

E840B846-5F8C-5AF2-827C-53CC0684CC17

##### Notes

Li (2023)

#### Fascellina

Walker, 1860

D5C8DEA1-7330-5B44-975C-95973E669F58

#### Fascellina plagiata

(Walker, 1866)

08522E77-289D-552F-ADC0-79FE4D72FE7D

##### Notes

Li (2023)

#### Herochroma

Swinhoe, 1893

06976599-2A66-530E-B41C-8D9B52E795CC

#### Herochroma cristata

(Warren, 1894)

587A9966-5361-54A4-9AC9-53F868F06151

##### Notes

Li (2023)

#### Hydrelia

Hübner, 1825

A66C4404-1734-5774-A6EF-A9377E844FA9

#### Hydrelia rufigrisea

(Warren, 1893)

CA058039-EBAF-5EEC-9235-F065EA8042CF

##### Notes

DSPA (2022)

#### Hyposidra

Guenée, 1858

F504F59D-9F75-51C5-A2DA-4449C0DE2610

#### Hyposidra infixaria

(Walker, 1860)

11C6F5C3-5249-5DF3-928E-417CF3C4E35D

##### Notes

Li (2023)

#### Hyposidra talaca

(Walker, 1860)

C0CD5999-1D07-5918-97A4-4F2997895EEE

##### Notes

Li (2023)

#### Idaea

Treitschke, 1825

8EF72AE4-9F2E-5979-A62B-369006F4C4BB

#### Idaea impexa

Butler, 1879

723E35DF-7BA0-5FF6-8E0D-4863EFD262D6

##### Notes

DSPA (2022)

#### Krananda

Moore, 1868

9D9B645F-42D6-5FA2-B1C5-F31EA079E7F9

#### Krananda latimarginaria

Leech, 1891

849A2CBD-7B61-5F9E-9D1E-C893D82355B1

##### Notes

Easton and Pun (1996)

#### Krananda straminearia

(Leech, 1897)

A1D84BC6-E222-5065-B1DA-73D58A28F225

##### Notes

Li (2023)

#### Menophra

Moore, 1887

44C45201-3D03-5EB3-AF70-A097DB86EEC6

#### Menophra tienmuensis

(Wehrli, 1941)

08081D36-0C28-5524-AB77-9EB9DD9567E1

##### Notes

Li (2023)

#### Nothomiza

Warren, 1894

F11CF06C-0349-54D4-98E4-015E54E4470B

#### Nothomiza flavicosta

Prout, 1914

90C2C9A9-1AB4-5379-A696-8D59CB0B2815

##### Notes

Pun and Batalha (1997)

#### Obeidia

Walker, 1862

A392ED8B-C489-5B23-92E2-7523585C800A

#### Obeidia tigrata

(Guenée, 1857)

8F6F5FAD-0CBA-5BAF-8CDD-CD4A64919B25

#### Obeidia tigrata neglecta

Thierry-Mieg, 1899

10EDBBD1-DAC3-5E37-A75A-0EE44AA7BBE2

##### Notes

Pun and Batalha (1997)

#### Ourapteryx

Leach, 1814

A29FF3DA-3345-5965-B80A-88B6EFB6D7EB

#### Ourapteryx clara

(Butler, 1880)

BFA928CF-BF06-5F50-8820-5450177BD32B

##### Notes

Pun and Batalha (1997)

#### Perixera

Meyrick, 1886

4E51EC21-BB5C-5CBA-9190-0C5F320DF8D2

#### Perixera minorata

(Warren, 1897)

DBB527C6-A207-527D-873E-4A3C460F4884

##### Notes

Li (2023)

#### Pingasa

Moore, 1887

76B96EA0-12C6-59D1-826C-0678D5041A27

#### Pingasa chlora

(Stoll, 1782)

07F35AF5-2DA8-5EF9-9491-7826DA2AAD3A

#### Pingasa chlora crenaria

Guenée, 1858

24F40A7B-32A5-5C01-ABD5-5C0920AEB676

##### Notes

Easton and Pun (1996)

#### Pingasa chloroides

Galsworthy, 1998

4F793CEE-E050-5179-8606-D9F005F2867D

##### Notes

Pun and Batalha (1997)

#### Pingasa ruginaria

(Guenée, 1858)

D03F6EBD-7720-5965-AEC9-5BDFA02A1BAA

#### Pingasa ruginaria pacifica

Inoue, 1964

BC88392F-9E6E-5931-BD5D-8BFE2C1296BF

##### Notes

Li (2023)

#### Problepsis

Lederer, 1853

070B5174-E2B4-5C61-B3B5-F123244576C7

#### Problepsis paredra

Prout, 1917

7CB7EBA9-6099-5AA6-9747-8A2EC7C91B23

##### Notes

Pun and Batalha (1997)

#### Psilalcis

Warren, 1893

5E2E0DD5-77DF-5022-96BF-D54F448F1AAD

#### Psilalcis breta

(Swinhoe, 1890)

8ED01765-7B3C-5777-A3B7-F26C44AB027A

##### Notes

Li (2023)

#### Pylargosceles

Prout, 1930

8AF4B613-95FC-51E5-AE7E-15903D176461

#### Pylargosceles steganioides

(Butler, 1878)

5CE0C2F8-9514-57BD-A13E-A7A868B67A84

##### Notes

Li (2023)

#### Ruttellerona

Swinhoe, 1894

F72848E3-2D17-5918-9355-38B41EC22FE1

#### Ruttellerona pseudocessaria

Holloway, 1994

EB2A6494-368E-581F-9835-33D3719CBAFE

##### Notes

Li (2023)

#### Scopula

Schrank, 1802

16F4E6EC-3E2F-50E6-915E-EA8DD2CA594B

#### Scopula personata

(Prout, 1913)

92C4C714-1998-5230-A876-F57230093646

##### Notes

DSPA (2022)

#### Thalassodes

Guenée, 1857

2DB9B321-81C9-59DC-AA13-DDF7086C1A4D

#### Thalassodes immissaria

Walker, 1861

33A93618-C6F3-5B7A-BCE8-4CEB3900D7C9

##### Notes

DSPA (2022)

#### Timandra

Duponchel, 1829

D2F4940E-D2A0-593D-9455-AFD80EE4E3BE

#### Timandra comptaria

Walker, 1863

AE321F2E-02C3-5D4B-B312-D24D18074974

##### Notes

Pun and Batalha (1997)

#### Timandra recompta

(Prout, 1930)

1A04B29F-9B25-5FF9-8519-3D7AF7B3DFE9

##### Notes

Li (2023)

#### Traminda

Saalmüller, 1891

DD146685-00F1-54B4-B614-C6A7726A1166

#### Traminda aventiaria

(Guenée, 1857)

59ADFD6D-9D96-53DD-91B8-224028F87BC5

##### Notes

Li (2023)

#### Hesperiidae

2CC54C9B-DD93-5FE8-8155-78DEE767C9E7

#### Ampittia

Moore, 1881

5F786FB5-C217-5683-85A7-BBAAE890D0D2

#### Ampittia dioscorides

(Fabricius, 1793)

5EF5F75F-7E34-52BD-BA6F-53BA2E418508

#### Ampittia dioscorides etura

(Mabille, 1891)

906C8C01-98FC-5A45-8190-022942E29919

##### Notes

MBD (2022)

#### Astictopterus

Felder & Felder, 1860

9A7B6F68-7AA9-5041-A57F-94C17798CB80

#### Astictopterus jama

Felder & Felder, 1860

67FD4C38-76DE-528B-AA72-EC11EB077554

##### Notes

Pun and Batalha (1997)

#### Baoris

Moore, 1881

1FA226A6-E927-5783-A7A1-E79F26666727

#### Baoris farri

Moore, 1878

6A66A45D-133F-54C3-94A2-B84F412922E6

##### Notes

DSPA (2022)

#### Borbo

Evans, 1949

FE8B6C13-0A1D-560D-A733-EAB27B6ED764

#### Borbo bevani

Moore, 1878

BA8836E5-4E15-5626-B6C7-ABB90F66D891

##### Notes

DSPA (2022)

#### Borbo cinnara

(Wallace, 1866)

0CBAA294-AF10-5F8B-80A9-0EE4E13F0C27

##### Notes

MBD (2022)

#### Caltoris

Swinhoe, 1893

C31CB595-394D-57C1-86AF-5AAE82729307

#### Caltoris bromus

Leech, 1844

FD293A13-A53E-5200-8B84-AB5E96990B1F

##### Notes

DSPA (2022)

#### Choaspes

Moore, 1881

9045D279-E29C-5707-80BC-A2A6D4FA2504

#### Choaspes hemixantha

Rothschild & Jordan, 1903

12F9A41C-FC72-51F8-8596-DF94DB71457F

#### Choaspes hemixantha furcata

Evans, 1932

CBAC3FAF-4C36-51B7-84C1-4B854C3AE630

##### Notes

Easton and Pun (1997b)

#### Erionota

Mabille, 1878

7CFB09B5-E710-5717-97BF-D46FE645A0FC

#### Erionota torus

Evans, 1941

2ADCE146-409F-599A-83A5-2C6A393FE8C5

##### Notes

Pun and Batalha (1997)

#### Hasora

Moore, 1881

E70DBE49-6BB1-57D6-8BD7-1C9635621F43

#### Hasora anura

De Nicéville, 1889

45EF55E1-20E3-52C7-871B-A9113EFB9DD4

##### Notes

DSPA (2022)

#### Hasora chromus

(Cramer, 1780)

91053264-2F4B-5886-A6B7-35A8F2BB3409

#### Hasora chromus chromus

(Cramer, 1780)

1C36A226-8F09-5B37-855B-2014680AB04E

##### Notes

MBD (2022)

#### Hasora vitta

(Butler, 1870)

1E9FF9D2-2F8B-5653-8A4F-5FF58DC12DA9

#### Hasora vitta indica

Evans, 1932

80298D06-ECF6-5803-AAB0-5BD19EEB2AE4

##### Notes

MBD (2022)

#### Hyarotis

Moore, 1881

18C49DCA-03A6-51D4-A8D7-D57FA82FB21F

#### Hyarotis adrastus

(Moore, 1866)

3A7A7ECC-3472-5B3B-A728-755B688F6F77

#### Hyarotis adrastus prabus

(Moore, 1866)

1836D305-86CD-505A-B6D2-301698E82444

##### Notes

Pun and Batalha (1997)

#### Lotongus

Distant, 1886

FBACD008-06EF-5510-BE41-2DBB850DBAB7

#### Lotongus saralus

(Nicéville, 1889)

23138F07-AAA7-5549-BC4E-E3F02C61F9B7

##### Notes

MBD (2022)

#### Parnara

Moore, 1881

5E65A46A-1127-5B2F-8D76-51BDEFCEFBB3

#### Parnara ganga

Evans, 1937

A3856943-2AB6-5E1D-8535-3FFDC6B09959

##### Notes

MBD (2022)

#### Parnara guttata

(Bremer & Grey, 1853)

14B2FDF4-A234-5C3E-B296-A1EAF21DD5F6

##### Notes

MBD (2022)

#### Pelopidas

Walker, 1870

74FFA2AA-8D75-55AC-9F46-D59E8C1B8FA6

#### Pelopidas agna

(Moore, 1866)

C18FB7B0-6F42-5B77-B549-CDB6021BE5C7

#### Pelopidas agna agna

(Moore, 1866)

F64F1AC7-BA4D-5429-9FBE-832E4184F73D

##### Notes

MBD (2022)

#### Pelopidas assamensis

De Nicéville, 1882

9149B129-5E0A-55C8-A361-353DF9838F7C

##### Notes

DSPA (2022)

#### Pelopidas conjuncta

(Herrich-Schäffer, 1869)

7EB48552-D36E-5E97-B5F7-79390FDE6CF9

#### Pelopidas conjuncta conjuncta

(Herrich-Schäffer, 1869)

135065FA-6999-5039-98E5-1D31D5865847

##### Notes

Pun and Batalha (1997)

#### Pelopidas mathias

(Fabricius, 1798)

23351072-BB6A-57A9-BAC7-428C8DE52593

#### Pelopidas mathias oberthueri

Evans, 1937

F15710E0-6358-52A4-B382-BF3777E66EE5

##### Notes

Pun and Batalha (1997)

#### Polytremis

Mabille, 1904

D5DB1077-5871-5D4E-B7C1-75061C2CDEB2

#### Polytremis lubricans

(Herrich-Schäffer, 1869)

FC5E6612-D420-579A-9041-5E620710671D

##### Notes

MBD (2022)

#### Potanthus

Scudder, 1872

25E4C9B4-5627-5DBF-BB18-F09AE21189E7

#### Potanthus confucius

(Felder & Felder, 1862)

743BBA37-229D-54D4-BBA1-415C00035E13

##### Notes

MBD (2022)

#### Potanthus trachalus

(Mabille, 1878)

DCA56B4C-94A3-5B40-AB45-0B0B80109A14

##### Notes

MBD (2022)

#### Tagiades

Hübner, 1819

94F6AA91-297A-5EA1-A7C7-2853BC091C7F

#### Tagiades litigiosa

Möschler, 1878

79D5D402-0986-50DB-9A1F-C8707DE568F8

#### Tagiades litigiosa litigiosa

Möschler, 1878

31E69EFD-1554-53E8-A1AE-D28B669F67C0

##### Notes

MBD (2022)

#### Suastus

Moore, 1881

2C73B712-3B01-5E6D-BDFB-EEBBFE31608B

#### Suastus gremius

(Fabricius, 1798)

7762FDAF-158F-53D4-822C-AF69A5D19651

#### Suastus gremius gremius

(Fabricius, 1798)

96DE8C87-27CD-5754-A379-D3F2889ABE59

##### Notes

Pun and Batalha (1997)

#### Telicota

Moore, 1881

647C9CCB-7C1E-5967-B68C-9056DB0B6F9D

#### Telicota ancilla

(Herrich-Schäffer, 1869)

189535E5-D14A-5C1A-B1EA-D6F81B76ECB7

#### Telicota ancilla horisha

Evans, 1934

6CB6EEF0-6E5A-5148-BE5C-7412DC35B0AE

##### Notes

MBD (2022)

#### Telicota colon

(Fabricius, 1775)

83305A07-2C88-5218-8450-FFB4666D04DD

#### Telicota colon stinga

Evans, 1949

F20395AB-45FF-58BE-9B59-1524D170BB25

##### Notes

MBD (2022)

#### Telicota formosana

Fruhstorfer, 1911

7E1FD025-90A8-51B3-BE1F-1AC230D5E021

##### Notes

Pun and Batalha (1997)

#### Telicota ohara

Plötz, 1883

B4109ED7-9A1A-5E50-9148-C1426BD1D73A

#### Telicota ohara formosana

Fruhstorfer, 1911

A4562622-9FF3-510F-A472-A21565F0BB7B

##### Notes

Easton and Pun (1997b)

#### Udaspes

Moore, 1881

A4A19A11-82A2-5E30-8BFC-4C095BFCB392

#### Udaspes folus

(Cramer, 1775)

EFC13BA0-7067-5424-8156-F475DF6CF979

##### Notes

Pun and Batalha (1997)

#### Lasiocampidae

6B98994C-F4A7-54E7-9A9F-5DE48D6C2FFE

#### Dendrolimus

Germar, 1812

A64AF38E-8FCF-5C67-B143-8A562AA6504A

#### Dendrolimus punctatus

(Walker, 1855)

29FAC9D5-BFB9-59FD-BAC9-DCCACD75B043

##### Notes

Pun and Batalha (1997)

#### Euthrix

Meigen, 1830

3862E911-D406-5564-9560-B49D24BB42A6

#### Euthrix laeta

(Walker, 1855)

349C1CB6-49C2-5DD2-BCA0-7C6BE4BF4364

##### Notes

Li (2023)

#### Odenestis

Germar, 1812

3F344943-CF96-5F8C-ACC6-83C4315227E8

#### Odenestis vita

Moore, 1859

99B2A89F-A245-5185-8E0E-75053AEAC318

##### Notes

Pun and Batalha (1997)

#### Trabala

Walker, 1856

5F49A2C9-CEFF-5F7D-8722-57622C4E1EB1

#### Trabala vishnou

(Lefebvre, 1827)

97A6E0B4-95A4-5BA5-A17F-6B4359031960

##### Notes

Pun and Batalha (1997)

#### Limacodidae

D3D22413-0CE9-59F8-A83E-A51141CCBA75

#### Oxyplax

Hampson, 1893

F1306D9F-3954-5E0E-8044-F042928E7807

#### Oxyplax pallivitta

(Moore, 1877)

E41827EE-F5AD-51E1-9D1D-45EE3E4A9387

##### Notes

Li (2023)

#### Quasinarosa

Solovyev & Witt, 2009

DE78E977-52F2-50DF-9D83-9E72B1FDCB29

#### Quasinarosa corusca

(Wileman, 1911)

188F46A8-0E95-544A-B731-F4F4E54BB5CD

##### Notes

Li (2023)

#### Thosea

Walker, 1855

8BF925C1-A86A-5E54-913F-3CCBCEE04797

#### Thosea loesa

(Moore, 1860)

98B70ECA-7ECF-5131-96EC-FBF6E67C46B6

##### Notes

Pun and Batalha (1997)

#### Thosea sinensis

(Walker, 1855)

8369E149-1414-5E62-87C5-E4F8359E25DE

##### Notes

Pun and Batalha (1997)

#### Lycaenidae

7E972B9C-C126-5C0D-9CAC-672483659873

#### Acytolepis

Toxopeus, 1927

405F1EBC-520F-5A83-A98E-2C19008DF03F

#### Acytolepis puspa

(Horsfield, 1828)

3CCB5949-4AB2-5D46-B94F-86DE898C3D82

#### Acytolepis puspa gisca

(Fruhstorfer, 1910)

AEB2088B-8203-53C5-9314-0095BB1877AF

##### Notes

Mendes and Sousa (2007)

#### Acytolepis puspa myla

(Fruhstorfer, 1909)

21F7521D-C18F-5B86-B47C-7F95D0A23F8F

##### Notes

Pun and Batalha (1997)

#### Arhopala

Boisduval, 1832

074E75EB-A121-50E1-85E7-1AE40212763D

#### Arhopala bazala

(Hewitson, 1862)

74167B4E-24FA-5B7D-9C04-6A2201FF17E2

#### Arhopala bazala teesta

(Nicéville, 1886)

FF040258-E925-514C-B318-9E6BF59AF9B1

##### Notes

MBD (2022)

#### Artipe

Boisduval, 1870

7F877D8A-C4F9-56FD-8DD1-B7166575A64D

#### Artipe eryx

(Linnaeus, 1771)

123296BD-FC76-5A51-8E7D-BDA6BC873A4F

##### Notes

MBD (2022)

#### Catochrysops

Boisduval, 1832

F0173E7A-E81E-5D38-BDCE-3014F3D9E657

#### Catochrysops strabo

(Fabricius, 1793)

4724A00E-E5FD-5C9C-A280-9E97816E2A2F

##### Notes

MBD (2022)

#### Celastrina

Tutt, 1906

CF7BDB1E-DC19-5BF5-A5AB-4D8F3945D406

#### Celastrina lavendularis

(Moore, 1877)

F84F8B74-ED2C-5C9F-8279-769177936436

##### Notes

Huang et al. 2016

#### Chilades

Moore, 1881

DE7A3511-33E2-5267-A846-491827C5CCAA

#### Chilades lajus

(Stoll, 1780)

2E47B0EC-E56C-5925-AAD1-9664C3BD89BA

##### Notes

MBD (2022)

#### Chilades pandava

(Horsfield, 1829)

56E3E29A-2873-5AD9-B3B2-872BF55D3D0C

##### Notes

MBD (2022)

#### Cigaritis

Donzel, 1847

4B0FCE13-3F03-5A02-96DB-BDF72FE07641

#### Cigaritis lohita

(Horsfield, 1829)

CBA47585-8C26-5799-B170-C0CD106F5A9A

##### Notes

Pun and Batalha (1997)

#### Deudorix

Hewitson, 1863

956F5BB1-A6F0-577D-BAEE-2AE481BEDAC6

#### Deudorix epijarbas

(Moore, 1857)

99C74663-FEDB-5663-B96D-C2D163F0BE81

#### Deudorix epijarbas menesicles

Fruhstorfer, 1912

84D370E7-44E2-5E4A-BEB7-062F667E33D2

##### Notes

Pun and Batalha (1997)

#### Euchrysops

Butler, 1900

79185751-51EA-5D67-A7FC-75900F0FAD30

#### Euchrysops cnejus

(Fabricius, 1798)

E6174418-2BC8-5DCD-B93D-A9941A9D1F6D

##### Notes

MBD (2022)

#### Everes

Hübner, 1819

F5E6819E-8032-56E3-98C9-92FEAAD20836

#### Everes lacturnus

(Godart, 1824)

ECD5650C-7837-53DC-9706-8E2AF7A8A600

##### Notes

MBD (2022)

#### Jamides

Hübner, 1819

93CDE81B-B97F-5EFC-8035-8B30A31D0F0D

#### Jamides alecto

(Felder, 1860)

1B8C3235-D021-57E5-81F8-A4AC33424572

##### Notes

Huang et al. (2016)

#### Jamides bochus

(Stoll, 1782)

06B57E53-FB25-587D-8DA0-7C421B3530D5

##### Notes

MBD (2022)

#### Jamides celeno

(Cramer, 1775)

AE141725-8C01-5A54-8E9A-B62E1EC519AC

##### Notes

MBD (2022)

#### Iraota

Moore, 1881

A6F72C96-95D5-5476-BAEB-6125E23E5FBD

#### Iraota timoleon

(Stoll, 1783)

D44C4F3D-EC1E-5B61-A0DD-8CCDF533BFB5

#### Iraota timoleon timoleon

(Stoll, 1783)

AC54BC98-D769-53F4-BF0C-5070DC87FF0F

##### Notes

Pun and Batalha (1997)

#### Lampides

Hübner, 1819

EAC4B8EC-340C-5A01-AF8D-BA61696D4DA9

#### Lampides boeticus

(Linnaeus, 1767)

03583F5D-EA88-50A0-B585-6770CA992C5F

##### Notes

MBD (2022)

#### Nacaduba

Moore, 1881

0FC4202D-8878-5041-AF80-7044EE732988

#### Nacaduba kurava

(Moore, 1857)

66604E1D-1E70-51BD-AD11-DD176D00E83B

##### Notes

DSPA (2022)

#### Neopithecops

Distant, 1884

7BA1694D-6B05-5CA4-87FD-357180B0ED2F

#### Neopithecops zalmora

(Butler, 1870)

606F671A-6496-5127-B2BB-FADFC5B573AE

##### Notes

MBD (2022)

#### Pseudozizeeria

Beuret, 1955

95E57017-DC96-5AB5-AA6E-87D376F16C70

#### Pseudozizeeria maha

(Kollar, 1844)

2E503AFA-496F-5EAE-BD0A-6766544D1D79

#### Pseudozizeeria maha maha

(Kollar, 1844)

175FA16C-17B9-5D73-AEEE-974B95DC003E

##### Notes

Pun and Batalha (1997)

#### Rapala

Moore, 1881

C479713F-AF57-58B4-B641-51A763BEA2F3

#### Rapala manea

(Hewitson, 1863)

22289A86-935A-55AA-846C-31B6A3EBFE86

##### Notes

MBD (2022)

#### Rapala nissa

(Kollar, 1844)

79D66779-E251-533D-8713-CAF645CCA0BA

#### Rapala nissa nissa

(Kollar, 1844)

ADEEC6FA-65B3-50CF-9521-45072F28137D

##### Notes

Pun and Batalha (1997)

#### Spindasis

Wallengren, 1857

C602FEB7-9BF4-58CA-AD90-FE4263E94AEA

#### Spindasis lohita

(Horsfield, 1829)

07133366-18F7-5C67-B971-0F385E447A17

#### Spindasis lohita formosan

(Moore, 1877)

8D33AA3A-5EBE-50F9-A411-F3FA7DB6FB76

##### Notes

Pun and Batalha (1997)

#### Tajuria

Moore, 1881

BDA30E27-4A38-5D64-91AC-FC89F2E33936

#### Tajuria cippus

(Fabricius, 1798)

3ABC03CD-4420-50C8-94E7-889C30C10A84

##### Notes

DSPA (2022)

#### Zeltus

Nicéville, 1889

F3561EBF-81E5-5B23-9DF0-49CE1C4826FB

#### Zeltus amasa

(Hewitson, 1869)

F66B666B-2CFB-5506-A6AB-E08AE08E1606

#### Zeltus amasa amasa

(Hewitson, 1869)

4228993B-BAA9-56ED-9109-20110A4F4D23

##### Notes

Perissinotto et al. (2023)

#### Zizeeria

Chapman, 1910

027848BD-250B-50F3-BA1B-BA95EFB39DDE

#### Zizeeria karsandra

(Moore, 1865)

4D97E83F-6187-5A80-A4C9-5769BB7C3C77

##### Notes

Yang et al. (2011)

#### Zizina

Chapman, 1910

8E8999A5-629E-55DD-AC66-0A1D289E2750

#### Zizina otis

(Fabricius, 1787)

B68A71A8-5CFC-570B-BD98-DD9CF6FFB194

#### Zizina otis otis

(Fabricius, 1787)

2278E73E-2CC7-53EF-A6E6-5DB54EE6B927

##### Notes

Mendes and Sousa (2007)

#### Lymantriidae

AD055581-A05C-50C5-9826-E1AB17B78469

#### Aroa

Walker, 1855

7736DC61-4F52-56E7-B366-834D24E53475

#### Aroa substrigosa

Walker, 1855

D9555493-386A-5EF3-BA4A-582FE5F98BF1

##### Notes

Pun and Batalha (1997)

#### Dasychira

Hübner, 1809

8479D91D-D363-5E2E-94DE-603D25AEABD9

#### Dasychira chekiangensis

Collentte, 1938

638849AB-37B3-523D-BD9E-D9BE41F05BC3

##### Notes

Li (2023)

#### Euproctis

Hübner, 1819

E00AB6FE-A0B4-52CE-A5E8-B3D99A8B3CCA

#### Euproctis bipunctapex

(Hampson, 1891)

F9282388-9340-5618-9187-F6FB49CCBB13

##### Notes

Pun and Batalha (1997)

#### Euproctis catapasta

Collenette, 1951

6814C9B9-0E20-5D52-893E-8A0A6F3EE32B

##### Notes

DSPA (2022)

#### Euproctis decussata

(Moore, 1877)

A4AB7FA1-A30E-5C29-A654-19A5E87F1731

##### Notes

Pun and Batalha (1997)

#### Euproctis diploxutha

Collenette, 1939

39E80B4F-2488-5C6A-BCA1-4D172E875B14

##### Notes

Pun and Batalha (1997)

#### Euproctis fraterna

(Moore, [1883])

4C60B837-DB06-5F8A-AD61-DF7CE5AD792C

##### Notes

Pun and Batalha (1997)

#### Euproctis plana

Walker, 1856

D081B0D2-5AAC-53A2-9C28-5282B6A2F239

##### Notes

Li (2023)

#### Euproctis scintillans

(Walker, 1856)

3F1B5A72-3647-5B1F-B97D-5EF94FE14CA9

##### Notes

Pun and Batalha (1997)

#### Euproctis straminea

Leech, 1899

BDE9876A-4D59-51CB-A540-7D6928F421DB

##### Notes

DSPA (2022)

#### Euproctis taiwana

Shiraki, 1913

ED4709CA-5F99-5F21-914F-E6C7A4965E49

##### Notes

DSPA (2022)

#### Lymantria

Hübner, 1819

05F09F7C-73EB-5C79-9394-61AB0093BF0E

#### Lymantria dissoluta

Swinhoe, 1903

20CF8C61-FF9E-5B89-A835-A49AE0AA5707

##### Notes

Li (2023)

#### Lymantria serva

(Fabricius, 1793)

78EBE400-0795-530E-8E4A-633FC5E3C5A4

##### Notes

Pun and Batalha (1997)

#### Orgyia

Ochsenheimer, 1810

29599EA0-0167-5654-816E-2FF233F07255

#### Orgyia postica

(Walker, 1855)

77A21B8B-2813-51B7-86D3-64E6EBD75E21

##### Notes

Pun and Batalha (1997)

#### Perina

Walker, 1854

FECC0326-C015-59D3-94A4-981350AF552C

#### Perina nuda

(Fabricius, 1787)

6808D17E-2A89-5F76-B421-2F85D1F302B5

##### Notes

Pun and Batalha (1997)

#### Metarbelidae

204D76EC-2BD1-5244-BD10-8ADCDE2395C9

#### Indarbela

Fletcher, 1922

0290C345-6CE9-5D21-94EB-F3E2FE75203D

#### Indarbela dea

(Swinhoe, 1890)

FE370323-5A54-55CD-8E72-ED726C0B830D

##### Notes

Pun and Batalha (1997)

#### Noctuidae

DF9E3757-ACD3-5F1A-B92C-85E260582A48

#### Adrapsa

Walker, 1859

C8C0D355-DA1B-5B9D-B3E7-A2AF93BFFDC2

#### Adrapsa quadrilinealis

Wileman, 1914

7E70EECD-9FEA-5F74-BAA3-E39712F5FCEF

##### Notes

Li (2023)

#### Aedia

Hübner, 1823

96201ADA-7DA9-54D5-A3C4-BA3C083C7961

#### Aedia leucomelas

(Linnaeus, 1758)

1D211295-C7CC-5104-95A0-606052C34353

##### Notes

Pun and Batalha (1997)

#### Achaea

Hübner, 1823

4C3FBC4C-BB43-5CEF-A922-495C62A2D81B

#### Achaea janata

(Linnaeus, 1758)

84A865F9-96E4-5DF6-8093-336760AD1D36

##### Notes

Pun and Batalha (1997)

#### Achaea serva

(Fabricius, 1775)

41C144A2-7688-547D-BA3B-E902A3FE9C2A

##### Notes

Pun and Batalha (1997)

#### Acronicta

Ochsenheimer, 1816

76C94A1E-CBE3-57C4-8E89-48BF4C3FA499

#### Acronicta pruinosa

(Guenée, 1852)

A275FE40-B569-5DB9-85A5-3710C79AA971

##### Notes

Li (2023)

#### Agrapha

Hübner, 1821

9BD173B7-CB09-501E-A247-0BAFAD2DFF22

#### Agrapha agnata

(Staudinger, 1892)

5E00887B-84BE-5A2A-85BE-D9804B2676AD

##### Notes

Pun and Batalha (1997)

#### Agrapha albostriata

(Bremer & Grey, 1853)

B345F1E1-0143-52D8-A3B8-E90F000E9BA5

##### Notes

Pun and Batalha (1997)

#### Agrotis

Ochsenheimer, 1816

2CE5E010-65DB-5C21-B7B9-6AE0C97FBFD0

#### Agrotis ipsilon

(Hüfnagel, 1766)

FFCCF67E-627E-5E3C-AD7D-5415C0985F60

##### Notes

Pun and Batalha (1997)

#### Athetis

Hübner, 1821

7CC942BD-EBAD-59BF-B0E3-78A2E05ACEBE

#### Athetis bipuncta

(Snellen, 1886)

3B8B7D5A-8C12-5624-9E8E-300B407260C0

##### Notes

Li (2023)

#### Athetis nonagrica

(Walker, 1863)

072BBA54-A1F5-5C41-AD63-A53138CF25AB

##### Notes

Li (2023)

#### Athetis reclusa

(Walker, 1862)

F5AEA8BF-C5BF-55B5-A765-E8F03C119C30

##### Notes

Li (2023)

#### Athetis stellata

(Moore, 1882)

AFA2BC8C-7176-5975-95BB-0307C5B6E8B3

##### Notes

Li (2023)

#### Amyna

Guenée, 1852

DA15C65B-8909-522F-81DF-721C4FAF99BB

#### Amyna axis

(Guenée, 1852)

26CEB410-5DDE-54BD-8D84-6E37619A6E75

##### Notes

Li (2023)

#### Amyna punctum

Fabricius, 1794

1AB49068-04B7-5955-A60F-84FA52EA5BCC

##### Notes

DSPA (2022)

#### Analetia

Calora, 1966

C73578AA-4847-503F-B04D-0A89743622EA

#### Analetia micacea

(Hampson, 1891)

B5E0EBE9-CC55-5871-AFDF-ECFDB7383231

##### Notes

Li (2023)

#### Anisoneura

Guenée, 1852

D3B67972-37EF-5C74-AEA4-96450E185038

#### Anisoneura aluco

(Fabricius, 1775)

150CB8D0-F340-5B11-B92A-68EA2DABBCA3

##### Notes

Pun and Batalha (1997)

#### Anomis

Hübner, 1821

C2BAF90E-340F-567A-BE8B-AE9C1599000B

#### Anomis flava

(Fabricius, 1775)

21EC8FCB-6327-5DDF-AEC1-8DB77C1FE1EB

##### Notes

Li (2023)

#### Anomis fulvida

Guenée, 1852

2C938262-8924-52A4-B7CE-978829F81B63

##### Notes

DSPA (2022)

#### Anomis leucolopha

Prout, 1928

6B2BAEA1-63DD-506E-BB7B-582044E8518F

##### Notes

Li (2023)

#### Anomis mesogona

(Walker, 1858)

2E88191B-1AFF-5B90-B8D5-5E8228D542C3

##### Notes

Pun and Batalha (1997)

#### Anuga

Guenée, 1852

37B3E34F-FA95-575C-9538-BC9410F53EFA

#### Anuga multiplicans

(Walker, 1858)

2AF14ECD-05CE-523F-9FD9-86EF4703F5BE

##### Notes

Pun and Batalha (1997)

#### Arcte

Kollar, 1844

C46AFD63-B500-5717-B85E-E650F5BFEBA9

#### Arcte coerula

(Guenee, 1852)

268101D5-6BEE-54C9-8196-63F7DBED78E3

##### Notes

Li (2023)

#### Artena

Walker, 1858

03195DC6-B28B-52FA-9C4E-B052B8164D24

#### Artena dotata

(Fabricius, 1794)

E88121ED-477C-542B-A62A-BFF8C931F478

##### Notes

Pun and Batalha (1997)

#### Avitta

Walker, 1858

94C1FB98-DB26-5777-AE38-F9991DBF32F6

#### Avitta fasciosa

(Moore, 1882)

8D9DCA85-8A53-53F2-A00F-6298DA6B490A

##### Notes

Li (2023)

#### Asota

Hübner, 1819

5EAF7BB7-FA7D-5614-8A66-5B3D97AF5189

#### Asota caricae

(Fabricius, 1775)

2D77D402-4F87-55E0-ADBD-2E764D95A6BA

##### Notes

Pun and Batalha (1997)

#### Asota plaginota

(Butler, 1857)

EAC57818-4506-58B4-99F3-84080F63FBF1

##### Notes

Li (2023)

#### Asota heliconia

(Linnaeus, 1758)

F37204B6-7FDE-5DD2-96CF-D022AC2833DD

##### Notes

Pun and Batalha (1997)

#### Blasticorhinus

Butler, 1893

7C5766A8-2704-5422-B7AF-DBA9F6E03187

#### Blasticorhinus rivulosa

(Walker, 1865)

A5D88ED3-2BCC-570A-A7F6-6F680D72ABC3

##### Notes

DSPA (2022)

#### Bocula

Guenée, 1852

C571D8E2-3098-53AE-8B17-AF30F6C8D4D5

#### Bocula marginata

(Moore, 1882)

14B21711-3AF7-5970-AB88-2B0E26FD510F

##### Notes

Li (2023)

#### Brithys

Hübner, [1821]

3A58AFF4-D669-57CF-9148-A6F8CA5DE84F

#### Brithys crini

(Fabricius, 1775)

CA8405CD-0051-5080-8637-AB2425F11C12

##### Notes

Li (2023)

#### Callopistria

Hübner, 1821

E71401FA-FB63-5002-BA3B-74889E6AE631

#### Callopistria albistrigoides

Poole, 1989

3845496E-12BF-5D8A-A042-A1377A0FDAF5

##### Notes

DSPA (2022)

#### Callopistria albomacula

Leech, 1900

310917E6-2F7A-5462-9653-CF1B31FF90D6

##### Notes

Pun and Batalha (1997)

#### Calyptra

Ochsenheimer, 1816

288698A0-26A0-58AA-8783-A193AE98B37E

#### Calyptra minuticornis

(Guenée, 1852)

77FA0C52-0901-53C1-BB70-B04A288FDD3D

##### Notes

Pun and Batalha (1997)

#### Carea

Walker, 1857

9B3B574C-C1FC-5BC7-AED7-66A94E70EF90

#### Carea angulata

(Fabricius, 1793)

A830BCF2-EA39-5EC5-A076-FCAAAB3CB5E2

##### Notes

Pun and Batalha (1997)

#### Chalciope

Hübner, 1823

0D038F8F-CABA-512E-91D0-E3C282B8BE57

#### Chalciope mygdon

(Cramer, 1777)

B3A1A16A-C1FB-5EE6-A7C8-169C20A1E46A

##### Notes

Pun and Batalha (1997)

#### Chasmina

Walker, 1856

F5F85855-2915-507C-B259-AFA36D802BF9

#### Chasmina candida

(Walker, 1865)

47E2018F-FD29-589A-8A27-E785572A8B62

##### Notes

Li (2023)

#### Chlumetia

Walker, 1866

AD5F8513-CA1E-502A-A25B-83CD4763C709

#### Chlumetia transversa

(Walker, 1863)

2BB7F8A9-429D-5AE5-B4E4-21544E2156AF

##### Notes

Pun and Batalha (1997)

#### Chlumetia guttiientris

Walker, 1866

1261E763-BE26-5516-B74A-5926639BD87D

##### Notes

Li (2023)

#### Condica

Walker, 1856

42C9B795-F422-5FBA-8A3A-370EEE812A3B

#### Condica fuliginosa

Leech, 1900

49F9DC23-324B-53DF-B45E-BF6A414687F5

##### Notes

DSPA (2022)

#### Chrysodeixis

Hübner, 1821

A61CAC42-1D25-547D-B4F1-F5A0C89798C1

#### Chrysodeixis eriosoma

(Doubleday, 1843)

B36481EC-6BB3-5B3B-A087-66062FC56203

##### Notes

Li (2023)

#### Craniophora

Snellen, 1867

AB7C5DB3-8F45-5FC2-A189-B01A8FEED463

#### Craniophora fasciata

(Moore, 1884)

574E509B-2442-510F-AE4E-51C170837336

##### Notes

Li (2023)

#### Crithote

Walker, 1864

77E3CDE7-B2B0-593C-B8A6-8B2F8287BA29

#### Crithote pallivaaga

Holloway, 2005

2F18831D-D0B5-529A-BDE4-AE9299155069

##### Notes

Li (2023)

#### Dyrzela

Walker, 1858

127B4591-0696-5D87-8766-CB5F3177E345

#### Dyrzela plagiata

Walker, 1858

0D6A275B-45F4-5AB9-AE01-4E1EF6888ACF

##### Notes

Li (2023)

#### Dysgonia

Hübner, 1823

BFAE3BF5-D30B-590C-8E9F-EB3767801A8F

#### Dysgonia arcuata

Moore, 1877

E92CFF06-26D1-5674-B6AD-8B0B7218A9B2

##### Notes

Pun and Batalha (1997)

#### Dysgonia crameri

Moore, 1885

60D7AA57-7D06-5565-B3D3-836C05E8D433

##### Notes

Pun and Batalha (1997)

#### Dysgonia fulvotaenia

(Guenée, 1852)

61AE723E-6E0E-557C-A11B-5604E80E2EF9

##### Notes

Pun and Batalha (1997)

#### Dysgonia illibata

(Fabricius, 1775)

4226A652-D78A-5DF6-BDAD-564568486C52

##### Notes

Li (2023)

#### Dysgonia joviana

(Stoll, [1782])

91111600-EEAB-54F5-8AE9-58BBB6C6728D

##### Notes

Li (2023)

#### Dysgonia maturata

(Walker, 1858)

0E6D72D0-5A9E-5DCD-AED0-A36C31B29F0B

##### Notes

Pun and Batalha (1997)

#### Dysgonia palumba

(Guenee, 1852)

BA7C604E-FCD1-5DB5-A883-D78F1353D70C

##### Notes

Li (2023)

#### Dysgonia stuposa

(Fabricius, 1794)

2A7B100C-C1D7-537D-8429-9E8E0CDBD714

##### Notes

Pun and Batalha (1997)

#### Dysgonia umbrosa

(Walker, 1865)

A1ABC66E-7D2B-539E-A227-3BFB2A7085D9

##### Notes

Li (2023)

#### Ecpatia

Turner, 1902

050C9BB0-B47A-5427-8BFF-33629898D416

#### Ecpatia longinquua

(Swinhoe, 1890)

F8E912BA-18C9-5AA8-AD66-B818614A7281

##### Notes

Li (2023)

#### Entomogramma

Guenée, 1852

E57E5C76-9443-511C-AC61-3788D4555D90

#### Entomogramma fautrix

Guenée, 1852

08B85C27-9C67-5D4D-BFE0-BD5393F86B3A

##### Notes

Pun and Batalha (1997)

#### Episteme

Hübner, 1820

ABAE31AE-C917-5810-9EA7-CCF07BABC5AE

#### Episteme lectrix

(Linnaeus, 1764)

295C70AD-109F-5313-AFF9-E7A008A9BD92

##### Notes

Pun and Batalha (1997)

#### Ercheia

Walker, 1857

4EBC0A6F-2814-5803-8E0B-36C25A12232A

#### Ercheia cyllaria

(Cramer, 1779)

1CDDE24D-0CD8-5FE0-B5F4-DE7F03E51FB8

##### Notes

Li (2023)

#### Erebus

Latrielle, 1810

86DB439B-B494-5041-9FE8-9B653149D5EB

#### Erebus crepuscularis

(Linnaeus, 1758)

6C531706-3B25-592D-AAB9-6109A61B34AB

##### Notes

Pun and Batalha (1997)

#### Erebus ephesperis

(Hübner, 1823)

1A715DDC-74DB-535F-91CA-352F00003C52

##### Notes

Li (2023)

#### Erygia

Guenée, 1852

477A5664-BACE-5E3A-BF6C-96C38B44140E

#### Erygia apicalis

Guenée, 1852

E4B8CD93-18C4-5D5C-93DF-AF0205E2F4D4

##### Notes

Li (2023)

#### Ericeia

Walker, 1858

6B8242D4-3D5D-587A-95DB-35787E0BAB03

#### Ericeia fraterna

(Moore, 1887)

5516C29E-4980-5765-8128-D3928D4A63DF

##### Notes

Pun and Batalha (1997)

#### Ericeia inangulata

(Guenee, 1852)

DDFB940E-4165-52D6-BD38-D460AB67A437

##### Notes

Li (2023)

#### Ericeia pertendens

(Walker, 1858)

2A5D1F4F-4EC2-5638-99C1-94F8BC8C38C7

##### Notes

Li (2023)

#### Ericeia subcinerea

(Snellen, 1880)

A0165617-4D72-5C57-9B0D-D8844FBC2A0A

##### Notes

Li (2023)

#### Eudocima

Billberg, 1820

AF1812B1-2D28-552C-89FB-A49D15416FDB

#### Eudocima phalonia

(Linnaeus, 1763)

094A2E30-E9F4-5E79-8A64-5F2205C8C66E

##### Notes

Easton and Pun (1996)

#### Eudocima salaminia

(Cramer, 1777)

502C1D74-0D05-5DAA-BFDD-F77F929384FD

##### Notes

Pun and Batalha (1997)

#### Eudocima fullonica

(Clerck, 1764)

2750B0A8-9490-5D29-80D8-6F03E1897B4C

##### Notes

Pun and Batalha (1997)

#### Eudocima homaena

(Hubner, [1823])

3752CCD9-301E-5048-BAEF-BB56E03B4F43

##### Notes

Li (2023)

#### Euplocia

Hübner, 1819

4430E9C3-C618-57F2-BFD2-35448D7B1D4F

#### Euplocia membliaria

(Cramer, 1780)

B72B14CD-4673-5D59-A5D8-BA7A7FA5A153

##### Notes

Pun and Batalha (1997)

#### Eutelia

Hübner, 1823

80F6394B-9952-54CA-8E3B-599386632C73

#### Eutelia adulatricoides

(Mell, 1943)

38A766EB-6106-56C5-9F29-15C25C6FC5A2

##### Notes

Li (2023)

#### Flammona

Walker, 1863

1871F21C-BEB9-5448-AB62-CCFE1BA4B8E4

#### Flammona trilineata

Leech, 1900

902296E2-EA9B-5F4D-BA2E-05FA99724BDE

##### Notes

Pun and Batalha (1997)

#### Grammodes

Guenée, 1852

5E7E3813-7081-568D-8E0F-782636515206

#### Grammodes geometrica

(Fabricius, 1775)

F3D838E9-1CA9-5894-8187-78CE5F5D663E

##### Notes

Pun and Batalha (1997)

#### Hulodes

Guenée, 1852

2E55C205-2AB9-5FBA-A018-7440B5BAE44D

#### Hulodes caranea

(Cramer, 1780)

3A145A29-5846-586D-83D2-195DCAAB4C21

##### Notes

Pun and Batalha (1997)

#### Hydrillodes

Guenée, 1854

352C1F78-B834-5578-8BB9-69D927C43C21

#### Hydrillodes abavalis

(Walker, 1859)

AF92CA2E-7756-5FBC-9777-92D28EFE7A6B

##### Notes

Li (2023)

#### Hydrillodes repugnalis

(Walker, 1863)

99C257EE-7AB4-5791-89F5-587B9A73DC38

##### Notes

Li (2023)

#### Hypena

Schrank, 1802

DA3CAE0F-73CB-5E01-9C24-982914D34BFC

#### Hypena albopunctalis

Leech, 1889

B93505F1-303F-5F4C-B71F-F7322A9E61AC

##### Notes

Li (2023)

#### Hypena sagitta

(Fabricius, 1775)

759E2F75-4710-5D52-9A20-0482C47CCF4C

##### Notes

Pun and Batalha (1997)

#### Hypocala

Guenée, 1852

83706229-1097-54D6-9296-B1F7024DD9C7

#### Hypocala deflorata

(Fabricius, 1794)

2EB0AF76-805D-5512-B6C3-BB9286A38C06

##### Notes

Li (2023)

#### Hypocala subsatura

Guenée, 1852

3D8EBE7D-67EE-5B6E-BBEF-347057346CF8

##### Notes

Pun and Batalha (1997)

#### Hypocala violacea

Butler, 1879

FD711826-73C6-5540-BA1B-EBA7D461C91E

##### Notes

Li (2023)

#### Hypopyra

Guenée, 1852

155A10DE-1A5B-5DA5-ABB9-A7A05E5035EC

#### Hypopyra ossigera

Guenée, 1852

F98D3476-E27E-532A-AC57-DC30F677C6A2

##### Notes

Li (2023)

#### Hypopyra vespertilio

(Fabricius, 1787)

EDB30523-00FB-59C1-84E5-C3EAD798490D

##### Notes

Easton and Pun (1996)

#### Hypospila

Guenée, 1852

41CA3053-E714-5C32-8EE3-D29C3AF0BA38

#### Hypospila bolinoides

Guenée, 1852

985F3941-2BB4-5F8E-A671-34BF541C4015

##### Notes

Li (2023)

#### Ischyja

Hübner, 1823

9E69017B-FA69-5743-8A4E-127BF578DCE3

#### Ischyja manlia

(Cramer, 1766)

B51241E9-0E81-5A40-A2D9-98994339E046

##### Notes

Pun and Batalha (1997)

#### Lacera

Guenée, 1852

5EF8AE8E-ECC9-53C5-8087-D29755D520C3

#### Lacera alope

(Cramer, 1780)

7DD93400-7F7F-59D1-9B19-982A5987B2F4

##### Notes

Pun and Batalha (1997)

#### Laspeyria

Germar, 1810

6B17DCCB-5D08-5AFF-A3E5-D64133BB26D7

#### Laspeyria ruficeps

(Walker, 1864)

C8299587-AECD-54EF-B2D7-E517439764B6

##### Notes

Li (2023)

#### Leucania

Ochsenheimer, 1816

697F6C9F-7198-59C6-BDB4-62A72F18AF8A

#### Leucania insularis

Butler, 1880

0A4E8E22-3407-5B70-B6B2-AB0925294DF6

##### Notes

DSPA (2022)

#### Leucania loreyi

(Duponchel, 1827)

F47F6630-8236-5316-8B1B-9A18CE0545EF

##### Notes

Pun and Batalha (1997)

#### Leucania venalba

Moore, 1867

2E0E162D-EB62-570D-BE06-FC9868DD057F

##### Notes

Li (2023)

#### Leucania yu

Guenee, 1852

31317F28-2D0F-5BD9-B42D-8F2F0FB6B496

##### Notes

Li (2023)

#### Lopharthrum

Hampson, 1895

EF4AC731-24AF-5B8F-AB9D-1518463D6DFE

#### Lopharthrum comprimens

(Walker, 1858)

3BF0C179-AAD3-569D-A6A5-9059253217D4

#### Lophoptera

Guenée, 1852

E3ECD51D-4A0F-5FFC-8D9B-7BA21594A6FC

#### Lophoptera squammigera

(Guenée, 1852)

C389F6FC-C41A-5B57-9A65-9E931916A3A9

##### Notes

Li (2023)

#### Lophoruza

Hampson, 1910

20B25352-7D5E-5BA8-881F-0B0715F231AB

#### Lophoruza lunifera

(Moore, 1885)

17258F23-C4F0-558C-8BAD-7C62AB2CC725

##### Notes

Li (2023)

#### Lophoruza kuehni

(Hollway, 2009)

06A98294-BA37-5AC3-BFCB-B86DDA884383

##### Notes

Li (2023)

#### Metaemene

Hampson, 1910

223D0E4F-E521-5616-88E0-B728D9866215

#### Metaemene atrigutta

(Walker, 1862)

F0100164-84C0-5651-9A08-12182B7EBADF

##### Notes

Li (2023)

#### Mythimna

Ochsenheimer, 1816

3914EE7D-4E3B-5AA1-B00E-81A3CBC8F66F

#### Mythimna formosana

(Butler, 1880)

BD72C738-0270-5D78-9C4F-8DCA7B06E939

##### Notes

Li (2023)

#### Mythimna snelleni

Hreblay, 1996

F7568B8F-4FC6-54EC-82B3-2EB9D5BDFDD7

##### Notes

Li (2023)

#### Mocis

Hübner, 1823

61F32C3F-B6CD-56C7-A7CC-22B4DFBE9E5D

#### Mocis frugalis

(Fabricius, 1775)

34439BE5-086F-5F6A-893A-55417343413E

##### Notes

Pun and Batalha (1997)

#### Mocis proverai

Zilli, 2000

F28B236D-5E6F-54EC-B38C-AD250E2114CA

##### Notes

DSPA (2022)

#### Mocis undata

(Fabricius, 1775)

4F69EE8A-E8F3-558B-BD6C-D6DE30088734

##### Notes

Pun and Batalha (1997)

#### Nodaria

Guenée, 1854

04EA046C-581C-59FA-8243-810C3CFF1E2F

#### Nodaria externalis

Guenée, 1854

34A5A6C7-340E-530D-BBE3-4F2D915A27FF

##### Notes

Li (2023)

#### Ophiusa

Ochsenheimer, 1816

B3C37FCC-DDE1-5A96-8783-04E45CE1F4A1

#### Ophiusa disjungens

(Walker, 1858)

2152B654-B05C-5EB5-A387-BF74C4A62F2C

##### Notes

Li (2023)

#### Ophiusa trapezium

(Guenée, 1852)

3CA83EB1-A949-5C8F-8795-B77887C95582

##### Notes

Pun and Batalha (1997)

#### Ophiusa tirhaca

(Cramer, 1777)

D9CF4D5E-D7E1-5E2C-BBCC-1296F1FCBAE6

##### Notes

Pun and Batalha (1997)

#### Ophiusa triphaenoides

(Walker, 1858)

96D5A02F-5D1A-5FCA-B9D6-0DDB3F153AF9

##### Notes

Pun and Batalha (1997)

#### Ophisma

Guenée, 1852

04DDABDA-9B0E-5A05-9A8E-EA9F6C4C09BF

#### Ophisma gramta

Guenée, 1852

656664FE-8783-51D5-BA97-11E421C3CEBA

##### Notes

Li (2023)

#### Oraesia

Guenée, 1852

5E647C94-48FF-5A05-9848-BA030710C736

#### Oraesia emarginata

(Fabricius, 1794)

D8D7F8EE-20B9-5F13-BFD7-B5B13B997BD7

##### Notes

Pun and Batalha (1997)

#### Oraesia excavata

(Butler, 1878)

80BF65A4-4462-5F2B-A966-06C08E28E194

##### Notes

Pun and Batalha (1997)

#### Oraesia rectistria

Guenée, 1852

3E2D2F3D-3AEE-5538-AFB4-F8EA64C64B29

##### Notes

DSPA (2022)

#### Oxyodes

Guenée, 1852

2D774891-FB62-5BE9-9E22-DDA3ED6A35C5

#### Oxyodes scrobiculata

(Fabricius, 1775)

2291FEEA-22E0-593F-BDA8-DA7B250B5DE7

##### Notes

Pun and Batalha (1997)

#### Pangrapta

Hübner, 1818

670B3FAE-8172-5A95-9D60-C5EEAE5E4C6D

#### Pangrapta shivula

(Guenée, 1852)

BB2007A9-5738-58A2-A3C0-9BB7DBCAD517

##### Notes

Li (2023)

#### Pantydia

Guenée, 1852

0E9BC2FF-1101-5A2D-BD5C-65D51CBBF405

#### Pantydia metaspila

Walker, 1857

67A6C4E1-C971-59B4-9B35-9C10C4D17D16

##### Notes

DSPA (2022)

#### Pataeta

Walker, 1858

C462A9F7-F0C7-52CE-8DE1-A8B5D1E9CFF8

#### Pataeta carbo

(Guenée, 1852)

6769FF6C-5502-5BE0-8491-1B1428D03A34

##### Notes

Li (2023)

#### Pericyma

Herrich-Schäffer, 1851

3FDEB2AD-0D97-5762-82C9-72DA912E14C7

#### Pericyma cruegeri

(Butler, 1886)

CDBEAEE5-E4E8-5A42-8A3B-F79359227BE0

##### Notes

Pun and Batalha (1997)

#### Penicillaria

Guenée, 1852

B7F168FF-6493-5530-AD3B-EC9E50677F9F

#### Penicillaria maculata

Butler, 1889

FC3A3736-C3F2-50F9-86D4-F419E2BC58FF

##### Notes

Li (2023)

#### Polydesma

Boisduval, 1833

C7BE584B-5033-5916-9FB0-162D5698A4F9

#### Polydesma otiosa

Guenée, 1852

763BA574-C0E7-5E6D-8E1A-4FF8E69FD218

##### Notes

Pun and Batalha (1997)

#### Platyja

Hübner, 1823

671D2576-3FF1-5000-A0DF-892FA0B0BFE3

#### Platyja umminia

(Cramer, 1780)

11C1D3F0-C7B3-5FF1-A6CC-4F49D42AD99F

##### Notes

Li (2023)

#### Platyja torsilinea

(Guenée, 1852)

3FC7BA34-0983-5728-9174-6B54B8B90B0A

##### Notes

Li (2023)

#### Plusiodonta

Guenée, 1852

D299A1B0-B7F5-5A61-8BC6-63E0EBFDBBC4

#### Plusiodonta coelonota

(Kollar, 1844)

7B92D639-A652-5C56-9E1A-1A0C4D7B2AD0

##### Notes

Li (2023)

#### Prospalta

Walker, [1858]

BFF1BA56-1F40-5395-AA04-AC5AD944381A

#### Prospalta leucospila

Walker, 1858

31E8BFEE-41CB-5916-B324-1ABA83641261

##### Notes

Li (2023)

#### Psimada

Walker, 1858

E310A43D-8DEF-5D74-8CE8-172C00EFFF81

#### Psimada quadripennis

Walker, 1858

B79EFF18-985B-5794-933B-E133654F013B

##### Notes

Li (2023)

#### Sarbanissa

Walker, 1865

67BD45CC-D379-5AC1-B22B-F38E2383202C

#### Sarbanissa albifascia

(Walker, 1865)

F34E00FE-188C-5668-B732-C37D5F5BFBF5

##### Notes

Pun and Batalha (1997)

#### Saroba

Walker, 1865

6785DB85-875C-540A-B011-F03E143806C7

#### Saroba pustulifera

Walker, 1865

CF1CB9DE-19B0-5C84-9F34-A9CB72915D8A

##### Notes

Li (2023)

#### Sasunaga

Moore, 1881

E6E99CAD-E951-595F-8798-AF11C644B7EB

#### Sasunaga longiplaga

Warren, 1912

B3BD0124-2E63-52AB-BD67-CBC340B6AEFE

##### Notes

Li (2023)

#### Serrodes

Guenée, 1852

467E0B4C-E742-5039-9B33-E14A99F41E0A

#### Serrodes campana

Guenée, 1852

089A7B30-3542-552D-998C-BF0A24702FCA

##### Notes

Pun and Batalha (1997)

#### Simplicia

Guenée, 1854

63864CD8-791D-532C-A309-1139F2288E48

#### Simplicia cornicalis

(Fabricius, 1794)

414C9676-24F0-55B1-B6E0-4057F8F045E5

##### Notes

Li (2023)

#### Simplicia niphona

(Butler, 1878)

79846641-F2E4-58E7-8BE5-7C3115AB8166

##### Notes

Pun and Batalha (1997)

#### Simplicia xanthoma

Prout, 1928

BD767A2E-EE7E-57C3-86C7-88DCBD5AABB9

##### Notes

DSPA (2022)

#### Singara

Walker, 1865

2ED5CDDF-487A-58CD-B872-923748FA2749

#### Singara diversalis

Walker, 1865

9D151ED1-C841-5AA1-A0FA-D25182C52A77

##### Notes

Li (2023)

#### Spirama

Guenée, 1852

1916EE58-FDBC-5627-B5F6-B481831A60E3

#### Spirama retorta

(Clerck, 1764)

99ECA55E-DB3B-5105-9B5C-F079119A9A91

##### Notes

Pun and Batalha (1997)

#### Spirama helicina

(Hübhner, 1831)

B3C72403-4D68-56B7-A398-BEDF420FAEBC

##### Notes

Li (2023)

#### Spodoptera

Guenée, 1852

10C0C870-C6F2-589A-9D6A-7EC63FDD7FEC

#### Spodoptera cilium

Guenée, 1852

060E4963-4A04-5EC2-8D70-691670BF4F5C

##### Notes

Li (2023)

#### Spodoptera exigua

(Hübner, 1808)

18570851-9747-58E5-A13F-B6DF4C8CA936

##### Notes

DSPA (2022)

#### Spodoptera litura

(Fabricius, 1775)

3099AA7D-2711-564C-BA65-B230CD243021

##### Notes

Pun and Batalha (1997)

#### Spodoptera mauritia

(Boisduval, 1833)

860B0840-E26B-591E-933F-972DEB75C8BB

##### Notes

Pun and Batalha (1997)

#### Spodoptera pecten

Guenée, 1852

23D73A0A-8A4F-5524-89C8-6FC231F4D756

##### Notes

Li (2023)

#### Spodoptera picta

(Guérin-Méneville, 1838)

1698D8C5-B74D-592F-89BA-C8AC41F2E27B

##### Notes

Pun and Batalha (1997)

#### Spodoptera festiva

(Donovan, 1805)

9BD35F7D-4A9B-5BA9-8E89-06E1D4AE7BC8

##### Notes

Pun and Batalha (1997)

#### Sympis

Guenée, 1852

E77F5DBB-40A7-524F-963B-CF372FC94148

#### Sympis rufibasis

Guenee, 1852

A51E4713-43D5-59CD-89D2-428040258E67

##### Notes

Li (2023)

#### Thyas

Hübner, 1824

A476DC33-930B-5C07-B948-C7FF84CCD845

#### Thyas coronala

(Fabricius, 1775)

125A6B31-AFD2-518D-B53D-60398634E1AE

##### Notes

Li (2023)

#### Thyas juno

(Dalman, 1823)

6123EBB4-807D-54E3-83E9-8C4F65E48F1D

##### Notes

Pun and Batalha (1997)

#### Trigonodes

Guenée, 1852

B04AD921-157A-5B60-83AE-4805EF1545F5

#### Trigonodes hyppasia

(Cramer, 1779)

19B1D22F-24EC-5D5E-B416-813F48BC942D

##### Notes

Pun and Batalha (1997)

#### Tycracona

Moore, 1882

61AC9EB3-5CA8-5D94-B91B-19C368937664

#### Tycracona obliqua

Moore, 1882

A6A2232B-EDB7-5068-A5BB-D7168334BD05

##### Notes

Li (2023)

#### Ugia

Walker, 1858

2CAFD539-EDDB-5D16-BC85-BC0A8A59A4B0

#### Ugia disjungens

Walker, 1858

10751297-1D25-5853-8DE5-54840F71ED35

##### Notes

Li (2023)

#### Xanthodes

Guenée, 1852

054FCDBC-D86E-51A9-B8AA-E50011A6DCCF

#### Xanthodes transversa

Guenée, 1852

F75BF590-6C87-58D4-BCDE-5CEC2A214CF4

##### Notes

Easton and Pun (1996)

#### Nolidae

33070999-A6AC-587E-AE93-258ED54474C8

#### Earias

Hübner, 1825

7AB11A93-D6D8-5E01-92FB-DF36FBEFCE5E

#### Earias flavida

Felder, 1861

82CC251F-5FEB-590A-979E-C6338A98C7E2

##### Notes

Li (2023)

#### Earias insulana

(Boisduval, 1833)

047812AC-860B-55D8-893B-0DF9CA647E2F

##### Notes

DSPA (2022)

#### Eligma

Hübner, 1819

13CF5348-5A8B-57C0-B45F-55D491BCD040

#### Eligma narcissus

(Cramer, 1775)

F95EB932-CC07-5DF1-B0EF-B03B449A0F93

##### Notes

Pun and Batalha (1997)

#### Iscadia

Walker, 1857

E0664D25-C6A6-52BE-ABB0-164CD7A3691F

#### Iscadia fusca

Pogue, 2014

9AC69D5B-5240-567B-8646-52A170DA77C7

##### Notes

Li (2023)

#### Iscadia inexacta

(Walker, 1857)

FA507214-2441-529D-AE72-F706EE4742E4

##### Notes

Pun and Batalha (1997)

#### Melanographia

Hampson, 1900

C2D06D5C-3DA8-5187-AAEB-45563D3AD6C0

#### Melanographia flexilineata

(Hampson, 1898)

53724016-01AC-5C0F-8275-F95C92CFF63B

##### Notes

Pun and Batalha (1997)

#### Narangodes

Hampson, 1910

117F2815-E2DD-58D7-855E-1E6AF216EEB9

#### Narangodes confluens

Sugi, 1990

AD78BA94-BC04-5967-A945-8EFFB6C6D0C0

##### Notes

Li (2023)

#### Nola

Leach, 1815

7C2BBB8F-AD9B-59B1-B3C9-28B6FED24749

#### Nola taeniata

Snellen, 1875

8AFF7B95-2889-56AC-AD2A-4506166B1F91

##### Notes

DSPA (2022)

#### Selepa

Moore, 1858

208D2F6A-26A1-545A-B6C0-8AFC3EB5DCC0

#### Selepa celtis

Moore, 1858

CF499F1E-7FD8-5580-B87E-1F71DAF0F202

##### Notes

Pun and Batalha (1997)

#### Notodontidae

290EC3DA-52CC-5672-B889-2A8272E5BCAC

#### Cerura

von Schrank, 1802

2FDA9C65-0A82-5FC9-A326-8C2ADEAC6B83

#### Cerura priapus

Schintlmeister, 1997

B09D6E6F-46E4-5A90-B384-312CD83D294D

##### Notes

Li (2023)

#### Cerura tattakana

Matsumura, 1927

B677B3C0-6B85-530F-A6DF-0216291F343C

##### Notes

Pun and Batalha (1997)

#### Neocerura

Matsumura, 1929

ECA1E304-D784-54C5-8851-4E6630DF79E0

#### Neocerura wisei

(Swinhoe, 1891)

15B1E0C4-04C5-5338-8EDB-D53978B978A0

##### Notes

Li (2023)

#### Stauropus

Germar, 1812

21254B66-8F27-5666-BC71-A93A737DF260

#### Stauropus alternus

Walker, 1855

B70A58AC-50AD-5CF8-9DD9-28F1AFA7677A

##### Notes

Pun and Batalha (1997)

#### Syntypistis

Turner, 1907

4DD4BE21-AA7B-5A32-A084-05FFD4418462

#### Syntypistis melana

Wu & Fang, 2003

96AF2536-BF16-5E17-A062-B14A0DDF5B88

##### Notes

Li (2023)

#### Nymphalidae

7BAE920D-C735-5691-8183-5BEFF28DB953

#### Argyreus

Scopoli, 1777

336B8320-7AE6-5EB3-A75B-882A0051BBCD

#### Argyreus hyperbius

(Linnaeus, 1763)

9507D506-D11F-5BD1-BA21-D8E0EA1719C2

##### Notes

MBD (2022)

#### Ariadne

Horsfield, 1829

7DCD140A-14D4-563F-BD36-29AD5342454D

#### Ariadne ariadne

(Linnaeus, 1763)

969BA620-EDDE-53B4-88AD-D8537FB1FB00

#### Ariadne ariadne pallidior

(Fruhstorfer, 1899)

17E0673C-C21C-5136-983E-D87CC6D5149B

##### Notes

MBD (2022)

#### Athyma

Westwood, 1850

ACD88E56-12CA-58ED-A1F4-2EA0E994C5D0

#### Athyma cama

Moore, 1858

5E0F3357-ADE4-5197-81BF-DA641E57936E

#### Athyma cama cama

Moore, 1858

E97F9C5D-A781-535B-ACFB-E72E9DCD381F

##### Notes

Mo et al. (2006)

#### Athyma nefte

(Cramer, 1780)

1EFAFFC2-B08D-55CE-8F94-D8E54B0ED2FF

#### Athyma nefte seitzi

(Fruhstorfer, 1906)

7C75218A-6DF1-5F11-A215-9E38CE5E22E5

##### Notes

MBD (2022)

#### Athyma perius

(Linnaeus, 1758)

3DF1E4AB-E07D-585C-A989-D3B33BA96C5E

#### Athyma perius perius

(Linnaeus, 1758)

EAF30674-4AE7-5C8F-8A7B-FE12FC8CC9A0

##### Notes

Pun and Batalha (1997)

#### Athyma selenophora

(Kollar, 1844)

896E7BCA-7DC3-5FC1-9685-1070919A58F0

#### Athyma selenophora leucophryne

(Fruhstorfer, 1912)

F93D369D-EC37-5302-ABA2-AC1EEE4B7411

##### Notes

MBD (2022)

#### Charaxes

Ochsenheimer, 1816

46FB4823-320C-59E6-9EB3-AB39E362BAEC

#### Charaxes bernardus

(Fabricius, 1793)

2F4A1819-5C03-58D1-85D5-8E77376FE221

##### Notes

Pun and Batalha (1997)

#### Cupha

Billberg, 1820

541533A3-8D5F-5A78-AF98-7E3157DE0980

#### Cupha erymanthis

(Drury, 1773)

65D4F50A-BA11-5F01-B427-C3DB536305DD

#### Cupha erymanthis erymanthis

(Drury, 1773)

7E1E902B-040A-5EE3-BC42-072D3627682B

##### Notes

Easton and Pun (1997b)

#### Cyrestis

Boisduval, 1832

DC83447F-EF8B-52BE-8FFD-1CA3705C3DB4

#### Cyrestis thyodamas

Doyère, 1840

512B142F-DA50-5E8F-BE72-33FB05AEC1E0

##### Notes

MBD (2022)

#### Danaus

Kluk, 1780

A560E809-6C35-57FC-A601-82234DCCAFB0

#### Danaus chrysippus

(Linnaeus, 1758)

73138D8C-7EEA-5350-973E-A0CA9C84F0E1

#### Danaus chrysippus chrysippus

(Linnaeus, 1758)

B2C7008A-CA0C-551E-A809-237780EFD9B5

##### Notes

Easton and Pun (1997b)

#### Danaus genutia

(Cramer, 1779)

9EF5DD73-260C-5545-956B-FEAC48861B19

#### Danaus genutia genutia

(Cramer, 1779)

9E770713-2DE2-554B-A0C9-2EB6D94C27C9

##### Notes

Easton and Pun (1997b)

#### Discophora

Boisduval, 1836

759413D7-D548-51CF-9A55-128C3044AEEF

#### Discophora sondaica

Boisduval, 1836

23A6FC11-0DDF-5E7A-9F50-A898CE16452B

#### Discophora sondaica tulliana

Stichel, 1902

36EE9128-F941-5682-AE0F-D8135B39EC5A

##### Notes

Pun and Batalha (1997)

#### Elymnias

Hübner, 1818

571CCC86-4379-519C-97DD-E2CE98A1E08D

#### Elymnias hypermnestra

(Linnaeus, 1763)

BD0DC7BB-FF94-571D-AECF-B1107345EE97

##### Notes

MBD (2022)

#### Euploea

Fabricius, 1807

D6FF20ED-D779-5FA0-942C-1FE192800925

#### Euploea core

(Cramer 1780)

2F8D767D-AD2C-55CB-865F-40B9BFC1D37C

#### Euploea core amymone

(Godart, 1819)

CFF8178D-2B44-54A1-BBB6-8EB60FB3C83C

##### Notes

Pun and Batalha (1997)

#### Euploea midamus

(Linnaeus, 1758)

9FA8BD51-23A5-5460-8FC9-F918D07A57C4

##### Notes

Easton and Pun (1997b)

#### Euploea mulciber

(Cramer, 1777)

BD8E0CF7-EB61-5321-9310-645D48D45197

#### Euploea mulciber mulciber

(Cramer, 1777)

F0885626-327D-5089-88D7-1714DD142976

##### Notes

MBD (2022)

#### Euthalia

Hübner, 1819

88B54873-B434-5078-A367-D30075CF49F8

#### Euthalia phemius

(Doubleday, 1848)

C71AF1E2-5644-5130-B9D4-19ED1BE4B5B4

##### Notes

MBD (2022)

#### Faunis

Hübner, 1819

DC17B885-FB2C-5078-A72D-4E7CDA889FFD

#### Faunis eumeus

(Drury, 1773)

F742DBD5-9453-5799-963F-A654743B751B

#### Faunis eumeus eumeus

(Drury, 1773)

998329CA-40F7-5983-82C4-2C12F859BBBF

##### Notes

Pun and Batalha (1997)

#### Hestina

Westwood, 1850

1190A8C8-584D-5E98-9625-671680F098B9

#### Hestina assimilis

(Linnaeus, 1758)

72ED6789-4474-5AAE-9A26-125F264E8821

#### Hestina assimilis assimilis

(Linnaeus, 1758)

C79A87C6-E2F6-59D5-89E4-EFF58BA92249

##### Notes

Pun and Batalha (1997)

#### Hypolimnas

Hübner, 1819

A97E6DB9-3831-5B33-AA73-6D2D3FEB076E

#### Hypolimnas bolina

(Linnaeus, 1758)

CCDD4091-23DA-505F-8B07-11959BFA8366

##### Notes

MBD (2022)

#### Hypolimnas missipus

(Linnaeus, 1764)

A448FF6B-9CA0-5401-AF19-228A63024175

##### Notes

Pun and Batalha (1997)

#### Ideopsis

Horsfiled, 1857

766DF448-3538-56D5-B59B-C8A0660368A6

#### Ideopsis similis

(Linnaeus, 1758)

39C5B125-E56D-5C81-85CB-1E493D9FBEEE

##### Notes

Easton and Pun (1997b)

#### Ideopsis vulgaris

(Butler, 1874)

FC3389E5-6903-537F-8DCE-722206C060F4

#### Ideopsis vulgaris contigua

(Talbot, 1939)

26572819-30AC-5DE4-A0D5-F922E43E9555

##### Notes

Mo et al. (2006)

#### Junonia

Hübner, 1819

B75BAECA-854E-58E8-9208-91C7A59A6D0F

#### Junonia almana

(Linnaeus, 1758)

05FA20E1-1FF0-558C-8396-5AFC71A2B66B

#### Junonia almana almana

(Linnaeus, 1758)

22EC7C49-0EF1-5988-92FF-F57F818CFFC5

##### Notes

MBD (2022)

#### Junonia atlites

(Linnaeus, 1763)

4DD4613E-236B-5E19-B10A-3EADC44E6F7D

##### Notes

MBD (2022)

#### Junonia hierta

(Fabricius, 1798)

0B0C8E3B-2995-50FA-958B-1703E6257EAB

#### Junonia hierta hierta

(Fabricius, 1798)

F2498136-1976-52AD-92F8-1AC1842D6113

##### Notes

Mendes and Sousa (2007)

#### Junonia lemonias

Linnaeus, 1758

C76AB317-D495-53DD-BA74-0F2AF7D6D245

##### Notes

DSPA (2022)

#### Junonia orithya

(Linnaeus, 1758)

9F830E97-8E0E-546E-9706-2A97F0AAB4B5

#### Junonia orithya orithya

(Linnaeus, 1758)

88552CE9-BE37-5E16-B21F-6A738658634C

##### Notes

Pun and Batalha (1997)

#### Kaniska

Moore, 1899

E67AF7F8-E934-52CB-8B49-8D950B9A8B07

#### Kaniska canace

(Linnaeus, 1763)

03C95059-7831-5866-9236-386FED9FEC8D

#### Kaniska canace canace

(Linnaeus, 1763)

1B8155C8-AF32-5494-97B2-6EBDC8813479

##### Notes

Pun and Batalha (1997)

#### Lethe

Hübner, 1819

F390F66D-37B0-5B6B-BFAB-D3FCA5DA023E

#### Lethe europa

(Fabricius, 1775)

14EBE52C-BEA8-57F7-B6EF-E0003E8ABD45

##### Notes

MBD (2022)

#### Lexias

Boisduval, 1832

30F27F19-10FE-5502-BD2D-71C76A94A9C7

#### Lexias pardalis

(Moore, 1878)

02BBC036-C7F4-5F36-B416-C054EAD9D0A0

##### Notes

MBD (2022)

#### Limenitis

Fabricius, 1807

23871281-E9EF-5512-9EBA-96601136BCDD

#### Limenitis sulpitia

(Cramer, 1779)

E28EE7B1-40F9-5D59-9635-7059A488A102

#### Limenitis sulpitia sulpitia

(Cramer, 1779)

8E7D23DE-5E5E-5F75-9AFA-A88441497E96

##### Notes

MBD (2022)

#### Melanitis

Fabricius, 1807

7A3E9B5A-3594-5513-9339-B23099479624

#### Melanitis leda

(Linnaeus, 1758)

BAFBE063-83FD-546E-9422-2B4FB1689D3A

#### Melanitis leda leda

(Linnaeus, 1758)

2BBE4F21-ACDC-586D-81F7-73BD2412F3F3

##### Notes

MBD (2022)

#### Melanitis phedima

(Cramer, 1780)

8902F0E1-9F4D-57C9-9780-F81ACA41DF68

##### Notes

Easton and Pun (1997b)

#### Mycalesis

Hübner, 1818

D0F58700-BCD6-5E80-B386-5696278125EC

#### Mycalesis mineus

(Linnaeus, 1758)

672BA80C-B097-5799-BF62-78B27FFAA374

#### Mycalesis mineus mineus

(Linnaeus, 1758)

EB9CEFBA-199F-5A90-861C-9570E7074368

##### Notes

MBD (2022)

#### Mycalesis sangaica

Butler, 1877

0EB2C780-46F0-5034-9EB1-2BC6A95AEAA9

##### Notes

DSPA (2022)

#### Mycalesis mucianus

Fruhstorfer, 1908

05729DF2-43CB-5C37-9110-3A6375348758

##### Notes

Huang (2019)

#### Neptis

Fabricius, 1807

D8030F3B-F727-5A5E-A6C6-AA24126F9016

#### Neptis clinia

Moore, 1872

04316B22-FA7C-5729-A1F1-9531C177AAD8

##### Notes

DSPA (2022)

#### Neptis hylas

(Linnaeus, 1758)

8D3B16CE-91F5-58F0-8E7A-E6BC6B13EEAA

#### Neptis hylas hylas

(Linnaeus, 1758)

A48352C2-824C-5873-84D8-89CACF55E00F

##### Notes

Mendes and Sousa (2007)

#### Neptis mahendra

Moore, 1872

D83C56BC-FD6F-5234-BF45-0CB44EB4AEEF

##### Notes

Mo et al. (2006)

#### Parantica

Moore, 1880

6B8DA4B8-1C36-512B-BECC-1E4FD86A71EA

#### Parantica aglea

(Stoll, 1782)

2716B544-B763-5BEA-8242-3140A96CF18A

#### Parantica aglea melanoides

Moore, 1883

14D2A23E-7C70-54E5-875B-60A22A3C10D3

##### Notes

MBD (2022)

#### Parantica melaneus

(Cramer, 1775)

A10054F1-0048-59C6-BE3E-D40DD3FA95A2

##### Notes

MBD (2022)

#### Phaedyma

Felder, 1861

35E8B105-6BA6-596A-88AC-B4610E2BC9AB

#### Phaedyma columella

(Cramer, 1780)

A4633CF0-E5A3-5B57-BA4A-7C45C85C364D

##### Notes

MBD (2022)

#### Phalanta

Horsfield, 1829

CBC7C0C1-F87F-566B-ACC3-E7865E92A95C

#### Phalanta phalantha

(Drury, 1773)

0962E83B-1CA1-5247-9591-47B4BEDDD8C2

##### Notes

Easton and Pun (1997b)

#### Polygonia

Hübner, [1819]

C53CB64B-6A94-55A2-A123-D07344699AA2

#### Polygonia c-aureum

Linnaeus, 1758

814D660F-7BCE-5F6D-AB3A-F34EDDA15505

##### Notes

Easton and Pun (1997b)

#### Polyura

Billberg, 1820

095E9D9A-BFD6-5C2F-82C4-874B8D24B285

#### Polyura arja

(Felder & Felder, 1867)

06D87053-5E69-538A-81E7-DE65296C5AF2

##### Notes

Pun and Batalha (1997)

#### Polyura athamas

(Drury, 1773)

BC3591FC-2A84-5FF4-83C1-417646816E19

##### Notes

MBD (2022)

#### Rohana

Moore, 1880

A44371A8-F810-5B2D-B1A9-56E4DEA5887D

#### Rohana parisatis

(Westwood, 1850)

585E33A7-130A-5C0A-A844-3A7E8312E3E4

#### Rohana parisatis staurakius

(Fruhstorfer, 1913)

363A2790-D1BD-5759-A27E-8E0AF66E0724

##### Notes

MBD (2022)

#### Symbrenthia

Hübner, 1819

CDCB9312-CB59-5D55-B9BB-77ECF8AF6B5B

#### Symbrenthia hippoclus

(Cramer, 1779)

CD1D7360-CA85-587E-9DBB-165B71E9221E

#### Symbrenthia hippoclus lucina

(Cramer, 1780)

6C318E0E-5572-559F-A730-B71868D34A0A

##### Notes

Easton and Pun (1997b)

#### Symbrenthia lilaea

(Hewitson, 1864)

2D51D5FE-E9F1-55E3-9A0F-84F53D06ED62

##### Notes

Huang (2019)

#### Tirumala

Moore, 1880

16E592B9-0CED-582C-8574-06775E2C4109

#### Tirumala septentrionis

(Butler, 1874)

99C04B62-37F5-58AF-A003-68A53B8672C4

##### Notes

MBD (2022)

#### Vanessa

Fabricius, 1807

9615D45C-C725-57C1-929B-964958B6F60E

#### Vanessa cardui

(Linnaeus, 1758)

F1842629-1432-5D02-B997-2AABCC5A7C39

#### Vanessa cardui cardui

(Linnaeus, 1758)

B1B54D9A-0984-5D3A-BE67-16B39922C61E

##### Notes

Easton and Pun (1997b)

#### Vanessa indica

(Herbst, 1794)

ED068691-D171-5D04-8817-F2CE3CC43B54

#### Vanessa indica indica

(Herbst, 1794)

97FE3A54-8FC9-5569-AE3C-B1DB7BFBF762

##### Notes

MBD (2022)

#### Ypthima

Hübner, 1818

89DD5CAC-F922-5F3A-91D7-A08724758782

#### Ypthima baldus

(Fabricius, 1775)

8D9168F5-6B52-572F-BBCC-A6A2C58C9B2E

##### Notes

Easton and Pun (1997b)

#### Ypthima lisandra

(Cramer, 1780)

E9E0DCB0-C343-503D-814A-026861E93F98

##### Notes

MBD (2022)

#### Papilionidae

650F5648-3A49-5864-B1BE-A1C950B029B7

#### Chilasa

Moore, 1881

2D69FD58-C489-5471-AA38-0A017FF29B7C

#### Chilasa clytia

(Linnaeus, 1758)

C3839EA5-2299-59DE-AD87-14F006D223A6

##### Notes

Pun and Batalha (1997)

#### Graphium

Scopoli, 1777

5DBDD87B-1E1F-5F7A-8BB6-2A6687EC6503

#### Graphium agamemnon

(Linnaeus, 1758)

2CC8CE37-41E0-58A7-A106-0E3541D6FC32

#### Graphium agamemnon agamemnon

(Linnaeus, 1758)

6F0308AD-14A5-59B7-8FAF-9025349F3819

##### Notes

Pun and Batalha (1997)

#### Graphium doson

9DE7D550-C95E-5CFC-8B0B-8E2089E236B6

#### Graphium doson axion

(Felder & Felder, 1864)

929B8565-43E1-53E9-84E6-2E846609B74B

##### Notes

Pun and Batalha (1997)

#### Graphium sarpedon

(Linnaeus, 1758)

FCECD454-1224-5ED9-9AEE-68947274CFEA

##### Notes

MBD (2022)

#### Papilio

Linnaeus, 1758

951E996C-0452-5ED0-BF17-EF7CA837A570

#### Papilio bianor

Cramer, 1777

0E6DFA4C-AB79-5072-AE1D-AFE6A89AB433

#### Papilio bianor bianor

Cramer, 1777

FF699872-C3FD-5018-97A8-3B96A805A520

##### Notes

Pun and Batalha (1997)

#### Papilio demoleus

Linnaeua, 1758

1EE069C6-71DE-529C-A05F-BAED8D608CDC

##### Notes

Pun and Batalha (1997)

#### Papilio helenus

Linnaeus, 1758

93E7A59E-488F-5A32-BC87-7AE145FEA082

#### Papilio helenus helenus

Linnaeus, 1758

CC8DFC7F-0D79-5CA1-BEEF-0D0053206CF3

##### Notes

MBD (2022)

#### Papilio memnon

Linnaeus, 1758

750EFEA7-2236-5687-9359-F8F6249CAA71

#### Papilio memnon agenor

Linnaeus, 1758

683F13FE-4D36-589F-9392-BDE8853E568E

##### Notes

MBD (2022)

#### Papilio nephelus

Boisduval, 1836

9C0CF652-3880-54B3-B4A5-A8731BA7F29C

#### Papilio nephelus chaonulus

Fruhstorfer, 1902

91D74C4F-850B-5F2D-A6F3-8864206CC502

##### Notes

MBD (2022)

#### Papilio paris

Linnaeus, 1758

EE2514D4-3A01-55A3-8C1A-CEFD5CC9AC5F

#### Papilio paris paris

Linnaeus, 1758

89D5EE2A-BC07-56C1-B719-B48EB3FE76D6

##### Notes

Pun and Batalha (1997)

#### Papilio polytes

Linnaeus, 1758

0B15D8FB-F334-58D4-8713-862A142563D7

#### Papilio polytes polytes

Linnaeus, 1758

F8E4CF08-B0ED-5D3A-B08B-F45AC428E37C

##### Notes

MBD (2022)

#### Papilio protenor

Cramer, 1775

501EA69F-98A8-5FE5-9E75-A6870F68E8D4

#### Papilio protenor protenor

Cramer, 1775

F024E918-AE00-5CAA-A9D1-E32FD90E4EF5

##### Notes

MBD (2022)

#### Papilio xuthus

Linnaeus, 1767

3FEB07E5-A655-5C33-94B9-AB5C76A94C3E

#### Papilio xuthus xuthus

Linnaeus, 1767

1082FDC3-6510-5B0F-9D8A-098952B14598

##### Notes

Pun and Batalha (1997)

#### Pathysa

Reakirt, 1865

9A8E8ED0-90F6-5F5D-A7B5-7FC1368434F9

#### Pathysa antiphates

(Cramer, 1775)

ACEEF16E-02B8-5637-B298-F415EE5F26BF

##### Notes

Pun and Batalha (1997)

#### Phyllocnistidae

2F1761E0-4E50-5FE2-B1FB-1888400B6594

#### Phyllocnistis

Zeller, 1848

D71EE270-3B32-58D8-BFE8-DCD45220E041

#### Phyllocnistis citrella

Stainton, 1856

326CB064-1C0B-5DED-9967-65516D36A542

##### Notes

Pun and Batalha (1997)

#### Pieridae

4CCA2232-0FF5-57DE-BE6E-51E1A6D8EB97

#### Appias

Hübner, 1819

F7D2D435-56D0-5482-92C8-ADB78302CCBE

#### Appias albina

(Boisduval, 1836)

16FF40D7-54C1-530D-85F0-81B62B1EB9BB

#### Appias albina darada

(Felder & Felder, 1865)

17C678DA-6928-5D3B-A885-EC056F9464C5

##### Notes

MBD (2022)

#### Catopsilia

Hübner, 1819

5832F5AB-38FA-5885-90F9-9FE6D781DD0F

#### Catopsilia pomona

(Fabricius, 1775)

D441FE48-4951-5707-89DF-15F7B965FC65

##### Notes

MBD (2022)

#### Catopsilia pyranthe

(Linnaeus, 1758)

4DA99F27-9F93-56DA-826E-025C79452CDE

##### Notes

Mendes and Sousa (2007)

#### Cepora

Billberg, 1820

3A46F716-BEC0-50C4-80D8-4726340661F3

#### Cepora nephelus

Fabricius, 1775

A40206F8-775C-56C1-A8A4-06BDD070E177

##### Notes

Huang (2019)

#### Cepora nerissa

(Fabricius, 1775)

9E9F9740-331A-5D3A-BDC4-15850F37B7F5

##### Notes

Easton and Pun (1997b)

#### Delias

Hübner, 1819

9DDC1969-94F8-56C1-B296-7EA9BFF9943C

#### Delias aglaja

(Linnaeus, 1758)

67E443D4-52A1-58EB-A72D-1D60824858AF

##### Notes

Easton (1992)

#### Delias hyparete

(Linnaeus, 1758)

F23CD871-DF79-505A-93A6-DB3C055EFA44

#### Delias hyparete hierte

(Hübner, 1818)

7B1E9746-3B7B-5C96-9CCB-EF9D8622040B

##### Notes

Pun and Batalha (1997)

#### Delias pasithoe

(Linnaeus, 1767)

E5698741-9421-5712-8F1E-32D48E0C0EA9

#### Delias pasithoe porsenna

(Cramer, 1776)

1FAC4233-0A8C-5F86-A3C7-478117FAFFCC

##### Notes

Easton and Pun (1997b)

#### Eurema

Hübner, 1819

B0F9A4F1-282E-57CE-AD6A-AEF0FA37F0EC

#### Eurema blanda

(Boisduval, 1836)

4452F264-1253-510D-8FF9-F71FD4ED901A

##### Notes

Pun and Batalha (1997)

#### Eurema brigitta

(Stoll, 1780)

96AED687-5A3A-5761-B231-084A40F4390A

##### Notes

Pun and Batalha (1997)

#### Eurema hecabe

(Linnaeus, 1758)

363849F0-CE46-5B08-8684-B3EA0E1DBF06

##### Notes

Pun and Batalha (1997)

#### Eurema laeta

(Boisduval, 1836)

CB15A96E-11AF-53C9-B001-956968E7FF55

##### Notes

Mendes and Sousa (2007)

#### Hebomoia

Hübner, 1819

1B7711AC-46E5-5A87-A384-CA7C884F2988

#### Hebomoia glaucippe

(Linnaeus, 1758)

71AC7779-E2DA-5568-93AB-8C118D2A9833

##### Notes

Easton and Pun (1996)

#### Ixias

Hübner, 1819

1000025F-C0D2-580F-B0BF-F39422DE3788

#### Ixias pyrene

(Linnaeus, 1764)

71798497-FC7D-5E2D-B0C4-FE1B0E59820E

##### Notes

Easton and Pun (1997b)

#### Pieris

Schrank, 1801

4CC1DEB4-FD66-5EBF-99F0-C53DDDC38047

#### Pieris canidia

(Sparrman, 1768)

5D6E3C81-A8B8-5BB4-9700-B1101A4C9A2C

##### Notes

Easton and Pun (1997b)

#### Pieris rapae

(Linnaeus, 1758)

157DB8AC-19B2-55EB-8BA5-7E318083330D

##### Notes

Easton and Pun (1997b)

#### Prioneris

Wallace, 1867

ACB111C6-0391-596D-8B6C-FFF76C134006

#### Prioneris thestylis

(Doubleday, 1842)

842FB4AE-4AB1-511D-A088-088C3276E778

##### Notes

Easton and Pun (1997b)

#### Plutellidae

2B84777D-97E4-5E70-9184-1A2A8ACD5EEF

#### Plutella

Schrank, 1802

C8C7056E-01C7-5AAB-9A01-DBD546CDD9BD

#### Plutella xylostella

(Linnaeus, 1758)

2FBEB2BE-AFCE-5315-977E-A56B9A8AD37C

##### Notes

Pun and Batalha (1997)

#### Psychidae

4FE02AC8-26D9-5990-949F-88547575007D

#### Acanthopsyche

Heylaerts, 1881

5CA0B37E-AACA-5262-B8FD-99CE34B2E858

#### Acanthopsyche subteralbata

(Hampson, 1892)

67C0948B-4BE5-573D-98E6-F4653AE682FA

##### Notes

Pun and Batalha (1997)

#### Chalioides

Swinhoe, 1892

9E91E2BF-ED7F-5B94-B545-C9EAC7DEF29D

#### Chalioides kondonis

Kondo, 1922

D025A5E4-C86F-53BC-A41D-6A73FDBD2AFA

##### Notes

Pun and Batalha (1997)

#### Clania

Walker, 1855

8430CA9B-ACE0-5A70-9045-A966AAB326B0

#### Clania minuscula

Butler, 1881

7B9F4EAA-C146-5D35-AD46-F252B79371DB

##### Notes

Pun and Batalha (1997)

#### Clania variegate

(Snellen, 1879)

F89844B6-8120-57EB-812A-982003CA9805

##### Notes

Pun and Batalha (1997)

#### Pterophoridae

FA8E9A46-CCE6-595B-8757-1CB5BD2074F3

#### Adaina

Tutt, 1905

E8826CDB-054A-5FE4-AA70-BB3FAE7E0A49

#### Adaina microdactyla

(Hübne, 1813)

8170EC54-EE57-5518-8277-3993BE1C249A

##### Notes

Li (2023)

#### Nippoptilia

Matsumura, 1931

1656D1D0-DB3B-51CB-A2F5-B843AE4C6535

#### Nippoptilia cinctipedalis

(Walker, 1864)

425AB182-23EE-546B-9346-3AAA235158EF

##### Notes

Li (2023)

#### Pyralidae

FD4F4B03-DDF0-52ED-B5B0-DCB2EC22BBD4

#### Arctioblepsis

Felder & Felder, 1862

261FFD5D-83B2-570D-BF28-14314414C043

#### Arctioblepsis rubida

Felder & Felder, 1862

697ADD33-94DD-5ABD-9F6A-B55E5EF98F2E

##### Notes

Li (2023)

#### Arippara

Walker, 1863

FABD1543-F96D-5CD7-A5DA-39A723D3965C

#### Arippara indicator

Walker, 1864

2119585A-F0BF-59A4-9428-73BE04B9DF8C

##### Notes

Li (2023)

#### Etiella

Zeller, 1839

020D819D-B276-52AB-808A-439448A57012

#### Etiella zinckenella

(Teritschke, 1891)

EDF1EAE2-E0BA-51B0-BBC6-E87DCF34CBB7

##### Notes

Li (2023)

#### Endotricha

Zeller, 1847

FB7BF76F-1D4D-53C0-877B-ADD6A1C19193

#### Endotricha costoemaculalis

Christoph, 1881

E3668F95-1CDF-5BBB-A738-499AC15FAA32

##### Notes

DSPA (2022)

#### Endotricha olivacealis

(Bremer, 1864)

4D290609-3681-5AF4-A362-7E4046CF4844

##### Notes

Li (2023)

#### Endotricha ruminalis

(Walker, 1859)

CED8D185-C508-50B3-BB3B-255ECCE3E73B

##### Notes

Li (2023)

#### Herculia

Walker, 1859

528A464E-7FD5-5929-A45A-5E1B2C9FDBAA

#### Herculia pelasgalis

(Walker, 1859)

E936E52D-7A95-5FD4-A795-07A541D3392E

##### Notes

Li (2023)

#### Hypsopygia

Hübner, 1825

0B655697-20E5-5323-8A2D-8215086CE978

#### Hypsopygia nannodes

(Butler, 1879)

4EE67818-EED7-545F-8EE1-47269CB85BC1

##### Notes

DSPA (2022)

#### Hypsopygia nonusalis

(Walker. 1859)

4D730E48-F8A5-5F65-8059-C085674B49D0

##### Notes

Li (2023)

#### Hypsopygia repetita

(Butler, 1887)

E5889BCF-98BF-52A4-A27E-02FE2B954E5C

##### Notes

Li (2023)

#### Locastra

Walker, 1858

EF7EB7B2-6A57-5E1C-8C6D-C9D39A8BA754

#### Locastra muscosalis

(Walker, 1866)

3B5FF8C1-F199-564D-90A5-CCA3047C4C8C

##### Notes

Li (2023)

#### Orthaga

Walker, 1859

8EC8F637-893A-504C-B35F-1A9E4D21B22B

#### Orthaga achatina

(Butler, 1878)

9BE49664-8408-5D8A-A630-9E9FBF63C985

##### Notes

Pun and Batalha (1997)

#### Orthaga olivacea

(Warren, 1891)

1E07413A-6B1B-5247-8BC0-521CEC890487

##### Notes

Li (2023)

#### Piesmopoda

Zeller, 1848

41D606E8-EB42-57D8-9F8F-C66B7C8B82D4

#### Piesmopoda semilutea

(Walker, 1863)

BF65B080-3F95-5565-8CFB-C5A7B420D2C4

##### Notes

Li (2023)

#### Pyralis

Linnaeus, 1758

19638E71-355D-5206-9852-BC75C7A4A10B

#### Pyralis pictalis

(Curtis, 1834)

FA21B1F2-B11E-5E5C-A280-EEAE82D66278

##### Notes

Pun and Batalha (1997)

#### Riodinidae

AF62F8F9-2DA4-51A2-BEB2-24571ABF81C7

#### Abisara

Felder & Felder, 1860

9DBB0ACE-FEC5-5C10-BEC0-92D7AFC6680A

#### Abisara echerius

(Stoll, 1790)

62E6B899-3036-556D-8525-60BC70AB26D8

##### Notes

MBD (2022)

#### Zemeros

Boisduval, 1836

DD95D78B-3222-50D9-8D93-4B185AC7FAF2

#### Zemeros flegyas

(Cramer, 1780)

B5EB9F10-2C0F-54FE-8417-B210B9477F79

#### Zemeros flegyas flegyas

(Cramer, 1780)

B7CDB264-BC6D-5FAE-AB29-704467408208

##### Notes

Pun and Batalha (1997)

#### Saturniidae

D8EF3B2B-0A93-5706-8CCC-340BFEAEDA2C

#### Actias

Leach, 1815

BA9505D8-C306-5D67-A40A-1BAA5CF3CBA1

#### Actias selene

(Hübner, 1807)

D6325C05-5E68-5FA3-82DC-A9FA102E3D2E

#### Actias selene ningpoana

Felder, 1862

2021C5FB-E577-5788-A3A9-348DFEFCF0DF

##### Notes

Pun and Batalha (1997)

#### Samia

Hübner, 1819

39BCFF85-2B31-53EC-8168-4AD39C24C490

#### Samia cynthia

(Drury, 1773)

F5ED96D4-AB00-5767-B7A2-D8AE09394036

##### Notes

Pun and Batalha (1997)

#### Sphingidae

81566AE5-8F72-55D8-8554-0E538938F9C8

#### Acherontia

Laspeyres, 1809

85F5A180-C60E-520C-943B-6F1E3AAEADEF

#### Acherontia lachesis

(Fabricius, 1798)

0EE981D9-4F6A-5802-92E9-A97EDB89B9F4

##### Notes

Pun and Batalha (1997)

#### Acherontia styx

(Westwood, 1847)

3E7E95E0-F759-5362-9217-D82F06BB6148

##### Notes

Pun and Batalha (1997)

#### Agrius

Hübner, 1819

5F14944D-10C0-57FB-ADBD-7C32E3BD2C1B

#### Agrius convolvuli

(Linnaeus, 1758)

BEFB6B80-392F-5D7E-B419-823EFE1D069E

##### Notes

Pun and Batalha (1997)

#### Angonyx

Boisduval, 1875

72544884-569B-5CDD-9AF8-911FB73A94EE

#### Angonyx testacea

(Walker, 1856)

A1A04BFE-8ACF-5CAD-8076-7ABD690ED03F

##### Notes

Li (2023)

#### Cephonodes

Hübner, 1819

7FC65D31-FC1A-51C3-8E01-623D5A531B5C

#### Cephonodes hylas

(Linnaeus, 1771)

3A92B4E1-D208-5CDE-A4D6-4FB5230A1697

##### Notes

Pun and Batalha (1997)

#### Clanis

Hübner, 1819

0BFCF593-8729-55B5-97A0-1E6EBB9A3D84

#### Clanis bilineata

Walker， 1866

27421E68-40AD-5CFD-B158-09069BDC9AB5

##### Notes

Li (2023)

#### Daphnis

Hübner, 1819

4F0F9632-6576-5EB7-9AF8-E9B2F05FC17A

#### Daphnis hypothous

(Cramer, 1780)

4CBEF67F-2976-5806-920E-2D3BDC696EA2

##### Notes

Pun and Batalha (1997)

#### Daphnis nerii

(Linnaeus, 1758)

267431E6-B155-5F0D-BE5F-9AAEE3C2FB3C

##### Notes

Pun and Batalha (1997)

#### Daphnis placida

(Walker, 1856)

09FE7208-51E3-5D79-BB78-82B47113C4F1

##### Notes

DSPA (2022)

#### Eupanacra

Cadiou & Holloway, 1989

F4AE81F8-9603-5784-B663-0A725F916ADF

#### Eupanacra mydon

(Walker, 1856)

2E7881F2-8633-5FC8-8CE6-625163462CFF

##### Notes

Easton and Pun (1996)

#### Hippotion

Hübner, 1819

19EAB1C7-B8D4-552C-9F4E-FE3D5CC6B5B3

#### Hippotion celerio

(Linnaeus, 1758)

EAD858B2-C846-5FF7-923C-F06E538C160C

##### Notes

Pun and Batalha (1997)

#### Hippotion rafflesi

(Moore, 1858)

9AADB653-279A-5B4B-A81E-447D41F52E85

##### Notes

Pun and Batalha (1997)

#### Macroglossum

Scopoli, 1777

C8CB5DAD-3A02-58C6-AF78-9924ED29D5A4

#### Macroglossum belis

(Linnaeus, 1758)

58189955-7479-51E9-8761-BBBD22BFD01B

##### Notes

Easton and Pun (1996)

#### Macroglossum corythus

Walker, 1856

9583EC59-DFA5-5F66-AB57-93E3CFC6417E

#### Macroglossum corythus luteata

Butler, 1875

79C780CD-154F-5799-A4BA-08EE614552A0

##### Notes

Pun and Batalha (1997)

#### Macroglossum divergens

Walker, 1856

60D37088-648E-53B2-AA96-80D03C2B15BD

#### Macroglossum divergens heliophila

Boisduval, 1875

CFC1C92C-E7CC-501D-AD96-B9FB45622DCC

##### Notes

Pun and Batalha (1997)

#### Macroglossum fritzei

Rothschild & Jordan, 1903

8CCFE07E-9120-57F2-BB01-608A3CA0B914

##### Notes

Li (2023)

#### Macroglossum pyrrhosticta

Butler, 1875

B0122BB7-9738-5EEA-8C07-9C143A2BDF23

##### Notes

Pun and Batalha (1997)

#### Macroglossum saga

Butler, 1878

190729C8-D8A4-59B4-AE78-CA96F5457CE3

##### Notes

Li (2023)

#### Macroglossum sitiene

Walker, 1856

DE0F7598-2B7C-57AE-A1DF-8CC0FB517C8D

##### Notes

Easton and Pun (1996)

#### Marumba

Moore, 1882

C3B17317-80E0-5423-B26B-94790A866A20

#### Marumba dyras

(Walker, 1856)

8124D776-BF95-543C-BB8C-B78CEB615DCE

##### Notes

Pun and Batalha (1997)

#### Marumba gaschkewitschi

(Bremer & Grey, 1853)

5D19ACFD-839D-59F7-A510-7AE4E952299E

#### Marumba gaschkewitschi complacens

(Walker, 1865)

BDCC0B42-2D16-5B3D-A34B-935281F7D2DB

##### Notes

Pun and Batalha (1997)

#### Marumba sperchius

(Ménétriès, 1857)

CD101F1F-247A-52D2-B670-A7C86619C6E0

##### Notes

Li (2023)

#### Neogurelca

Hogenes & Treadaway, 1993

AC659B39-FFBF-5D28-AAC8-719579C778B7

#### Neogurelca hyas

(Walker, 1856)

C2968942-EE3F-5188-83B4-0AFFD2754AFA

##### Notes

Pun and Batalha (1997)

#### Pergesa

Walker, 1856

648E27F8-917A-5BB9-9728-E459D6FB8B8A

#### Pergesa acteus

(Cramer, 1779)

958CFFD3-0138-573D-B6EC-7968DE80A122

##### Notes

DSPA (2022)

#### Psilogramma

Rothschild & Jordan, 1903

E9509B4B-7A4B-507A-BEBD-6B0D007CA463

#### Psilogramma menephron

(Cramer, 1780)

DE7FA037-9847-5C95-89BE-A0858E4915B3

##### Notes

Pun and Batalha (1997)

#### Theretra

Hübner, 1819

DFF6A910-C874-5CD9-BE81-74384A5B9D53

#### Theretra alecto

(Linnaeus, 1758)

428F4C10-1851-59E9-A91A-E58BBCAA27C3

##### Notes

Pun and Batalha (1997)

#### Theretra clotho

(Drury, 1773)

6C7B49C6-39BA-53A4-880B-C6A71D51D165

#### Theretra clotho clotho

(Drury, 1773)

09FD26E8-41F7-5AAE-B7E7-0221F11D612F

##### Notes

Pun and Batalha (1997)

#### Theretra latreillii

(Macleay, 1826)

EEC242FE-46A6-52E6-933F-B8BFC822750A

#### Theretra latreillii lucasii

(Walker, 1856)

730A1CB5-6825-5524-9E5D-1069F0371465

##### Notes

Pun and Batalha (1997)

#### Theretra nessus

(Drury, 1773)

E27247A7-1478-592E-9FD7-965E7277B95E

##### Notes

Pun and Batalha (1997)

#### Theretra silhetensis

(Walker, 1856)

B20FE5EB-5923-5FAD-BD21-61015AA3E8ED

#### Theretra silhetensis silhetensis

(Walker, 1856)

89916BFC-33CB-5A27-B9EB-A0C55A383B96

##### Notes

Pun and Batalha (1997)

#### Theretra suffusa

(Walker, 1856)

1147830A-F720-5DC5-810C-59EB21387FEA

##### Notes

Pun and Batalha (1997)

#### Tortricidae

511E81BE-1A8D-5C53-8C51-4932C5EC235F

#### Archips

Hübner, 1822

8653EA27-BC2F-572F-BA27-34E295D3351B

#### Archips sayonae

Kavabe, 1985

75908CE6-1705-5F2E-9914-CDFC1864E7F1

##### Notes

Pun and Batalha (1997)

#### Cryptophlebia

Walsingham, 1899

AC3C6D66-AC05-51E1-B3E3-26A53DD054C0

#### Cryptophlebia ombrodelta

(Lower, 1898)

20B0C6E4-A180-5C27-9D16-D40457A6A1F2

##### Notes

Pun and Batalha (1997)

#### Diplocalyptis

Diakonoff, 1976

EF99AD0F-F629-55ED-ABBA-550E1E048E26

#### Diplocalyptis tennuicula

Razowski, 1984

1B73A809-AC69-54B8-AEA8-B6BF7624B573

##### Notes

DSPA (2022)

#### Neoanathamna

Kawabe, 1978

D326CF20-31F0-5472-A6A4-346E1568DE2C

#### Neoanathamna negligens

Kawabe, 1978

CD7E386E-5D3A-52CD-98F0-F67D9F65401F

##### Notes

DSPA (2022)

#### Rhadinoscolops

Obraztsov, 1968

CA57FAB6-DA0E-562F-9065-14E0A4099E78

#### Rhadinoscolops koenigana

(Fabricius, 1775)

D88344FB-AD17-5998-87E9-EA53AFBE2D2C

##### Notes

Pun and Batalha (1997)

#### Uraniidae

ED84AD4D-C4B0-5D22-ADD6-94F843126B26

#### Micronia

Guenée, 1857

07FAF84D-57D4-5960-8B21-4F95D38649CE

#### Micronia aculeata

Guenée, 1857

BFA814E7-1C39-54E5-9541-400DD649665A

##### Notes

Pun and Batalha (1997)

#### Lyssa

Hübner, 1823

5B59449F-D438-5182-B40A-0F9B96558927

#### Lyssa zampa

(Butler, 1869)

8122BD9A-3113-5620-8A12-21891F972DB9

##### Notes

Pun and Batalha (1997)

#### Phazaca

Walker, 1863

DA726F32-1FF1-5FC6-846F-FC0D82205C9C

#### Phazaca kosemponicola

(Strand, 1916)

8DA62E9F-FE3C-543F-BED9-81F29402852F

##### Notes

Li (2023)

#### Zygaenidae

52F2913E-D33C-5A6F-AB42-A48E5F243900

#### Cyclosia

Hübner, 1820

2E096E1A-6767-5027-8988-B1ADAA50B1D6

#### Cyclosia papilionaris

(Drury, 1773)

5B10B34C-0677-5058-BF40-0DD241E9693A

##### Notes

MBD (2022)

#### Gynautocera

Guérin-Méneville, 1831

C4CC8181-C401-5B4C-9444-1B1A708A7A7A

#### Gynautocera papilionaria

Guérin-Méneville, 1831

75A58159-2AC5-5350-AAFE-4D919A843024

##### Notes

Easton and Pun (1996)

#### Histia

Hübner, 1820

DFC37BF4-8FBC-504F-A38D-9848F1F9C104

#### Histia flabellicornis

(Fabricius, 1775)

B9ACAFB1-A456-56D4-B2FC-D14561C34466

##### Notes

DSPA (2022)

#### Pidorus

Walker, 1854

C1FD6244-4638-52F2-B407-B0EB952FBCA4

#### Pidorus gemina

(Walker, 1854)

E832957C-119B-5B3E-BF64-4E29030A5891

##### Notes

Pun and Batalha (1997)

#### Pidorus glaucopis

(Drury, 1773)

4E2B3CAB-3281-5980-AA04-B0AE08CCB9CF

##### Notes

Pun and Batalha (1997)

#### Thyrassia

Butler, 1876

9939ED2D-AF2F-57A4-B32D-70E1DAB40DCC

#### Thyrassia penangae

(Moore, 1859)

3C4EDABF-4F1B-5D48-BD27-26709D41519F

##### Notes

Li (2023)

#### Trypanophora

Kollar, 1844

108E77A5-27EB-5E9F-8E07-91BA7820DC35

#### Trypanophora semihyalina

Kollar, 1844

DF54FDEB-91AD-58AA-AC38-643A79664B84

##### Notes

Li (2023)

#### Coleoptera

2C52636F-24B9-58B1-87A9-209E29D02280

#### Bostrychidae

736CA3B1-53C3-5044-BE7B-2E55743E9D11

#### Bostrychopsis

Lesne, 1899

88321C0B-2E89-55CD-9472-39350ED1A0A1

#### Bostrychopsis parallela

(Lesne, 1895)

F885D2E3-2FA5-5A87-AADB-F7E92C28FF7D

##### Notes

Easton (1992)

#### Dinoderus

Stephens, 1830

F8621170-195F-5880-8CE2-CF1A22E940DA

#### Dinoderus minutus

(Fabricius, 1775)

1FB7FC02-217F-5E7E-997D-A8BEED822A89

##### Notes

Zhang (2006)

#### Heterobostrychus

Lesne, 1899

43108416-F357-5CBE-85C8-131DE89A8AB7

#### Heterobostrychus aequalis

(Waterhouse, 1884)

69D8718F-BCBF-56C7-908F-4901EC932011

##### Notes

Pun and Batalha (1997)

#### Bruchidae

10A03B5B-DC04-5E3B-AB2D-5B4085D83F84

#### Callosobruchus

Pic, 1902

586F9846-9169-5319-BCD3-C9A9A13395B1

#### Callosobruchus chinensis

(Linnaeus, 1758)

EFE991D3-F2B6-5353-8251-B25CD58F2542

##### Notes

Mo et al. (2006)

#### Chalcophora

Dejean, 1833

63F522EB-9623-52AF-B855-90DA75335967

#### Chalcophora japonica

(Gory, 1840)

9C11509C-7E2E-5F88-AB74-1057AA657316

##### Notes

Pun and Batalha (1997)

#### Spermophagus

Schoenherr, 1833

CEEA9108-B033-5AFA-A111-7D2D5BCD4FFC

#### Spermophagus albonotatus

Chûjô, 1937

2D0742E7-0A78-59E1-899E-000C5AAD3904

##### Notes

Pun and Batalha (1997)

#### Carabidae

99466765-FCCA-5A8C-8589-482ADA77DF74

#### Chlaenius

6653894E-34F5-524F-B2BD-C73A50A91F44

#### Chlaenius bioculatus

Chaudoir, 1856

A8966E62-A261-59FC-83DC-B11FD0B4F5D7

##### Notes

Pun and Batalha (1997)

#### Cicindela

Linnaeus, 1758

EEDED0AF-4362-5452-B4C3-EABEDC11351E

#### Cicindela anchoralis

Chevr, 1845

43E350F2-5EEF-547B-BAE3-E072ACC20CFA

##### Notes

Zhang (2006)

#### Cicindela aurulenta

Fabricius 1801

1024A557-A8D9-5175-80E3-12623A6C3E9B

#### Cicindela aurulenta juxtata

Acciavatti & Pearson, 1989

2C614333-1CDC-5336-9C49-E19FE38EA973

##### Notes

Pun and Batalha (1997)

#### Cicindela kaleea

Bates, 1866

37E54D3D-4931-5FAD-BDA2-56E9525F3E8A

##### Notes

DSPA (2022)

#### Cicindela separata

Fleutiaux, 1894

68E29723-787B-5597-813A-536F40BAB349

##### Notes

Easton (1992)

#### Craspedοphorus

Hope, 1838

0DBC97DA-4DE1-5EB1-92FC-5A4A93D1D66F

#### Craspedοphorus mandarinus

(Schaum, 1854)

B5410CE4-4ED6-5919-9364-6DFB5D783511

##### Notes

Pun and Batalha (1997)

#### Odacantha

Paykull, 1798

F0C934BF-5AE1-5668-930D-F38F94E53EF9

#### Odacantha metallica

(Fairmaire, 1889)

6D8E5F61-1BA1-5A98-8643-4F1CE7C23B5A

##### Notes

DSPA (2022)

#### Pheropsophus

Solier, 1833

02773526-4421-5D3D-B609-B34AD400F660

#### Pheropsophus occipitalis

(Macleay, 1825)

A99CD7DD-4AFF-5DE6-9125-4DA103AD72CE

##### Notes

Pun and Batalha (1997)

#### Stenolophus

Dejean, 1821

D220E050-4E24-508B-88DE-4BC9B7ED7E7A

#### Stenolophus quinquepustulatus

(Wiedemann, 1823)

A7ED0A13-5577-5910-B99D-D24FB5B99AB3

##### Notes

DSPA (2022)

#### Cerambycidae

AD3B4A8A-CED5-5729-A7E9-B0ABA7908258

#### Aegolipton

Gressitt, 1940

E4DCFF38-4A8F-5BC4-8F71-4E67122B1FDD

#### Aegolipton marginale

(Fabricius, 1775)

05233656-A0DE-5368-9837-B9EEEE851801

##### Notes

Lin et al. (2021)

#### Aeolesthes

Gahan, 1890

5E033E60-7502-5781-B8C4-A10CA0945409

#### Aeolesthes induta

(Newman, 1842)

1A1416D5-C3EC-52F6-BB6A-62A0A60AFE1F

##### Notes

Lin et al. (2021)

#### Anoplophora

Hope, 1839

C6AB73D3-49F6-5677-AD34-A16EE346C740

#### Anoplophora chinensis

(Forster, 1771)

9B062280-D858-5522-BC43-13B562C5B351

#### Anoplophora chinensis chinensis

(Forster, 1771)

56271C69-67F2-5A8E-BF5D-706BBCC15712

##### Notes

Lin et al. (2021)

#### Apomecyna

Dejean, 1821

6DF4D2D0-6FCC-5A2E-8DA5-A67D39D85B79

#### Apomecyna longicollis

Pic, 1926

657605EA-530E-51F6-B1C3-D859739B3B04

#### Apomecyna longicollis longicollis

Pic, 1926

BCE3452E-0424-5AB2-AB52-DBE01075E38C

##### Notes

Lin et al. (2021)

#### Apomecyna saltator

(Fabricius, 1787)

40CC95F0-3993-547B-B9CE-850BCF0E877A

##### Notes

Lin et al. 2021

#### Apriona

Chevrolat, 1852

C1040BB7-30CB-5DB1-9DA3-283300AFD5F2

#### Apriona germari

(Hope, 1831)

47CA53A6-2CF2-52C6-93BD-E91FD2ACD137

##### Notes

Lin et al. (2021)

#### Aristobia

Thomson, 1868

5B7B897E-46AA-5A14-A6E5-C38BD268B3E4

#### Aristobia approximator

(Thomsonm, 1865)

856D9220-3492-5EC0-9A66-E772A1B43F4D

##### Notes

Lin et al. (2021)

#### Batocera

Castelnau, 1840

DA19B863-F303-5502-ADAB-AEF7FF7297B0

#### Batocera horsfieldi

(Hope, 1839)

026919B8-923D-5B91-939B-9C2A663EB330

##### Notes

Pun and Batalha (1997)

#### Batocera rubus

(Linnaeus, 1758)

33C33BB6-5408-55AB-8F78-B109FF9AF53C

#### Batocera rubus rubus

(Linnaeus, 1758)

4E11D994-DB45-553D-9920-CD24BE57F633

##### Notes

Pun and Batalha (1997)

#### Blepephaeus

Pascoe, 1866

A247F171-B3FD-5EF9-A906-2EDC6F9967B6

#### Blepephaeus subcruciatus

(White, 1858)

A260326F-C8B6-5B3E-8AC6-CFC10B3ABBFB

##### Notes

Lin et al. (2021)

#### Blepephaeus succinctor

(Chevrolat, 1852)

4E8A5AC8-BAF9-5979-A7D5-E2A584BAE68C

##### Notes

Lin et al. (2021)

#### Bumetopia

Pascoe, 1858

3E4ED311-0EAC-5E7B-9045-7AAC6D27416A

#### Bumetopia oscitans

Pascoe, 1858

7807186C-40E3-5FA3-B67C-3366F6ABD0CE

##### Notes

Lin et al. (2021)

#### Cephalallus

Sharp, 1905

EBA02F54-7860-5BA1-8D2F-C85CD0366D57

#### Cephalallus unicolor

(Gahan, 1906)

1704C163-212B-5B5B-B0A6-90AA3EA8D390

#### Cephalallus unicolor unicolor

(Gahan, 1906)

7CD9F1E9-02DA-50D6-910F-F84D58E9770B

##### Notes

Lin et al. (2021)

#### Ceresium

Newman, 1842

B911F295-B4A4-54DB-9AF8-003D5D0BC57C

#### Ceresium elongatum

Matsushita, 1933

6EBA6611-D385-52DD-9411-AEBCB2659E79

#### Ceresium elongatum elongatum

Matsushita, 1933

77901492-67D8-5DC1-9CA4-0512986D068C

##### Notes

Lin et al. (2021)

#### Ceresium longicorne

Pic, 1926

40D1B484-53A8-5133-B790-B95CA118505D

##### Notes

Lin et al. (2021)

#### Ceresium sinicum

White, 1855

DFC4B5E5-53A7-5587-B7C2-AF7311F701D5

#### Ceresium sinicum ornaticolle

Pic, 1907

AF4929F6-528B-5951-8853-42F6E8D90F43

##### Notes

Lin et al. (2021)

#### Ceresium zeylanicum

Yokoi, 2015

5C0EAA20-E3A0-5A53-9648-C773A5007F0D

##### Notes

Lin et al. (2021)

#### Chelidonium

Thomson, 1864

47D11992-A2D5-52A5-AF8A-3BA6D0503BE3

#### Chelidonium argentatum

(Dalman, 1817)

5AA9F53E-FB6C-5DEF-8DFE-9B20956D6212

##### Notes

Lin et al. (2021)

#### Chlorophorus

Chevrolat, 1863

0364C5A0-D1BA-5865-B9A7-74116C8AE0E3

#### Chlorophorus annularis

(Fabricius, 1787)

4C069BE5-6926-5EA3-A33E-442753AFD0EB

#### Chlorophorus macaumensis

(Chevrolat, 1845)

D092E24F-F56E-56D8-BF79-9A88D57856C1

##### Notes

Lin et al. (2021)

#### Chlorophorus macaumensis macaumensis

(Chevrolat, 1845)

7E208A80-261F-5F58-8C72-46D88897AA24

##### Notes

Lin et al. (2021)

#### Coptops

Audinet-Serville, 1835

04EC10E6-0709-5D50-B940-E031AB6F9BAD

#### Coptops licheneus

Pascoe, 1865

A7C94FB5-A7A2-53A2-80EA-6D24660A4C3B

##### Notes

Lin et al. (2021)

#### Coptops variegata

(Breuning, 1938)

40C0BE06-F11B-5E89-B40C-B5C9B7D06719

##### Notes

Pun and Batalha (1997)

#### Demonax

Thomson, 1861

9012A795-F358-5FC8-AA74-1BFA989B92AA

#### Demonax bimaculicollis

(Schwarzer, 1925)

2A126972-56F9-523F-BFED-992014FE8321

##### Notes

Lin et al. (2021)

#### Desisa

Pascoe, 1865

14BC647D-98C5-5E37-ADA1-4EE130F0795F

#### Desisa subfasciata

(Pascoe, 1862)

7B955001-7726-526C-BBD7-36D64F339018

##### Notes

Lin et al. (2021)

#### Embrikstrandia

Plavilstshikov, 1931

C2A639B9-8EDF-5C36-AABD-99AAE99AEEC0

#### Embrikstrandia unifasciata

(Ritsema, 1896)

53F9D2C8-214E-5F2A-9DCF-840F77F9C2EF

##### Notes

Lin et al. (2021)

#### Exocentrus

Dejean, 1835

391E2FA0-2882-5CEC-AE6E-D8CAD403869F

#### Exocentrus alboguttatus

Fisher, 1925

539A3B60-A47C-53FB-B85F-94BFD4332A3F

#### Exocentrus alboguttatus subconjunctus

Gressitt, 1940

5AC83FFB-B85B-550D-8319-C9E1D297DC7B

##### Notes

Lin et al. (2021)

#### Exocentrus formosofasciolatus

Kusama & Tahira, 1978

9332C4C5-18E8-5087-BFBC-3D88507E10BA

##### Notes

Lin et al. (2021)

#### Glenea

Newman, 1842

6EA9CD19-CE7B-53EE-A9DC-523356E4AE7C

#### Glenea cantor

(Fabricius, 1787)

FF4C27DD-E50F-5971-9D72-9CDE6DB33AE2

#### Glenea cantor cantor

(Fabricius, 1787)

2AD07519-82BF-5FA3-B7A6-B4870A83AA24

##### Notes

Lin et al. (2021)

#### Imantocera

Dejean, 1835

4B9169D5-2E02-5FA8-B7F6-2E66D90B94A4

#### Imantocera penicillata

(Hope, 1831)

B6157791-59F9-5327-A6B7-AB4E83F06035

##### Notes

Lin et al. (2021)

#### Kuegleria

Holzschuh, 2017

51A8DEDB-6884-56EA-9F48-91A8E96C8963

#### Kuegleria annulicornis

(Pic, 1935)

1BF33BAE-4093-5D41-8F18-ABAEE84B4958

##### Notes

Lin et al. (2021)

#### Megopis

Serville, 1832

B7E2D805-B5B5-5FD0-972D-027CECB48040

#### Megopis marginalis

(Fairmaire, 1775)

CC8763DD-AF3A-5D1F-9987-9B5776BCCC17

##### Notes

Lin et al. (2021)

#### Mispila

Pascoe, 1864

7A7E9018-D262-5865-A1F3-FE6EAA0C0954

#### Mispila tholana

(Gressitt, 1940)

C24C2AD8-FE58-593F-A361-671C7D882C5C

##### Notes

Lin et al. (2021)

#### Monochamus

Dejean, 1821

0D09EDC6-CDAE-5593-9604-239AF5B18EBB

#### Monochamus alternatus

Hope, 1842

9B5A7159-F7B1-5559-BA07-8AD7D915B421

#### Monochamus alternatus alternatus

Hope, 1842

94368813-1588-5162-8D49-B79B918A4C14

##### Notes

Lin et al. (2021)

#### Nysina

Gahan, 1906

0675B599-D785-5716-A4E5-3CEAB3A283CA

#### Nysina rufescens

(Pic, 1923)

8E16AD4E-03F5-5C0D-9C48-84194FBBA923

#### Nysina rufescens asiatica

(Schwarzer, 1925)

D0DC06DC-8262-5343-9C5E-2C2E50E114B1

##### Notes

Lin et al. (2021)

#### Oberea

Dejean, 1835

F7A1663B-E845-5655-AB1C-6BBE0C8A4AFE

#### Oberea ferruginea

(Thunberg, 1787)

98982ABB-33B4-5DE5-9D38-6B172F46916E

##### Notes

Lin et al. (2021)

#### Oberea walkeri

Gahan, 1894

CF7A3A31-7329-5FAF-8599-164097B0F452

##### Notes

Lin et al. (2021)

#### Olenecamptus

Chevrolat, 1835

4DC88118-BBC0-5368-99C4-80636E8AEEFF

#### Olenecamptus bilobus

(Fabricius, 1801)

8EE6B6E8-63C3-5A7F-9EBA-65CB62D38797

##### Notes

Lin et al. (2021)

#### Olenecamptus taiwanus

Dillon & Dillon, 1948

383F7926-40A9-54AE-9662-1BDEE77E3CD3

##### Notes

Lin et al. (2021)

#### Perissus

Chevrolat, 1863

4A817804-5127-52FF-982F-027357F1B240

#### Perissus indistinctus

Gressitt, 1940

38C5D002-FB23-50B2-8001-157E49CF892B

##### Notes

Lin et al. (2021)

#### Pothyne

Thomson, 1864

44D42B19-5EE1-5AF6-8061-61C1017C1D02

#### Pothyne rugifrons

Gressitt, 1940

AC396460-45E7-5305-A6B2-CFCB5D08CAC3

##### Notes

Lin et al. (2021)

#### Polyzonus

Dejean, 1835

722F886B-865D-56B8-8D7F-18251FB38410

#### Polyzonus sinensis

(Hope, 1842)

55CC4E72-66B1-5BB8-8520-FEDAA393FDF0

##### Notes

Lin et al. (2021)

#### Prosoplus

Blanchard, 1853

70345E2C-FD69-5F63-9B94-65A74BA195AA

#### Prosoplus bankii

(Fabricius, 1775)

2E43C9C9-F3CA-56C9-A8FD-F234D6FBEA9C

##### Notes

Lin et al. (2021)

#### Pseudoterinaea

Breuning, 1940

7C1E9F7E-D113-5553-9FF3-5A51DDEB2464

#### Pseudoterinaea bicoloripes

(Pic, 1926)

822DB4EC-08C0-5669-8062-C2836DA6A989

##### Notes

Lin et al. (2021)

#### Pterolophia

Newman, 1842

D09C429E-5F72-5F57-8E81-7E70A5586474

#### Pterolophia annulata

(Chevrolat, 1845)

CFB7A3FA-4D13-57F7-8940-02B7C9A1676B

##### Notes

Lin et al. (2021)

#### Pterolophia consularis

(Pascoe, 1866)

BE943895-FDF9-5B4B-BC0C-177A42AA8537

##### Notes

Lin et al. (2021)

#### Pterolophia crassipes

(Wiedemann, 1823)

D7057904-B555-5190-931D-1D24E5AAD349

##### Notes

Lin et al. (2021)

#### Pterolophia kaleea

(Bates, 1866)

230A3F30-81F3-56D5-880D-88E9615AF94C

#### Pterolophia kaleea inflexa

Gressitt, 1940

F9B53282-600B-5D05-9B48-5FDDB490965C

##### Notes

Lin et al. (2021)

#### Purpuricenus

Dejean, 1821

977A0BEF-47E9-537A-BFD3-CB1124876F53

#### Purpuricenus temminckii

(Guérin-Méneville, 1844)

1881EA85-F4CE-5C5B-9849-3ACCBB2F4F62

#### Purpuricenus temminckii sinensis

White, 1853

431C6C6A-D2F4-52BF-909F-72307935AA17

##### Notes

Lin et al. (2021)

#### Pyrestes

Pascoe, 1857

EF90DAE1-3A8F-5C91-BA34-654816940DC9

#### Pyrestes haematicus

Pascoe, 1857

77BC303A-5D0C-5A88-8863-F43332C5BFFA

##### Notes

Lin et al. (2021)

#### Rhytidodera

White, 1853

1F219D3F-7D2C-54CB-812F-35E7626F7E20

#### Rhytidodera integra

Kolbe, 1886

2298B6AA-9045-5204-9AB5-E79EA53251E2

##### Notes

Lin et al. (2021)

#### Rondibilis

Thomson, 1857

96BEAA26-213B-586E-B083-6D881C42B4F9

#### Rondibilis undulata

(Pic, 1922)

B1BFE697-E162-575B-B8D4-5093057ACC99

##### Notes

Lin et al. (2021)

#### Ropica

Pascoe, 1858

5B7B2178-C455-577B-AF59-107FC653A159

#### Ropica dorsalis

Schwarzer, 1925

32EF4078-0787-5FA7-B1AA-AC437BB29A0C

##### Notes

Lin et al. (2021)

#### Sophronica

Blanchard, 1845

4DB079A2-FFF8-5200-B73F-B30ADBC38CA8

#### Sophronica apicalis

(Pic, 1922)

2E39EFF2-9813-5BFB-AF18-39F2D9278E84

##### Notes

Lin et al. (2021)

#### Stromatium

Audinet-Serville, 1834

65CC38F2-12F1-5BA2-B42C-76FF5C0B2620

#### Stromatium longicorne

(Newman, 1842)

D55795DA-C811-59C5-BA7B-5814E43BD26B

##### Notes

Lin et al. (2021)

#### Sybra

Pascoe, 1865

4155CAC9-EB5C-568F-B49D-7DCF0C5D8403

#### Sybra marmorea

Breuning, 1939

E2CE8AAE-BA9C-5724-9D02-4D8EB420B5EB

##### Notes

Lin et al. (2021)

#### Sybra posticalis

(Pascoe, 1858)

B57710BD-D5C4-519E-8873-3566F34784CF

##### Notes

Lin et al. (2021)

#### Trirachys

Hope, 1843

D6A24F41-E739-5EC3-8DA7-A2E8AA86AE5A

#### Trirachys indutus

(Newman, 1842)

3600EFFD-044C-5A1B-BB66-BEF47C374BC5

##### Notes

Lin et al. (2021)

#### Eutaenia

Thomson, 1857

8C8C62B2-D03B-56BB-B3BF-91A3FD515BAC

#### Eutaenia tanoni

Breuning, 1962

F1C5E832-CE33-579D-AFB6-3330D5AD6518

##### Notes

Lin et al. (2021)

#### Xystrocera

Audinet-Serville, 1834

0673D06A-078C-574A-A1F2-386543BB10BB

#### Xystrocera globosa

(Olivier, 1795)

24C68737-FC0E-5BD1-A0BB-7F00EDAB9026

##### Notes

Lin et al. (2021)

#### Zotalemimon

Pic, 1925

74591B1D-87FD-5AB7-BDE4-A6FADE6A9B78

#### Zotalemimon ciliatum

(Gressitt, 1942)

CCF2CCD7-0508-5913-9E02-75035656416D

##### Notes

Lin et al. (2021)

#### Cetoniidae

E5EB264D-BD0A-5458-8980-312A6B200F03

#### Agestrata

Eschscholtz, 1829

CDB25F82-7F5F-51D4-81B9-167A76BC752A

#### Agestrata orichalca

(Linnaeus, 1769)

45766B23-03CA-5EAE-AE62-FA04FE33762E

#### Agestrata orichalca orichalca

(Linnaeus, 1769)

0B3E150B-534F-5AD8-96BB-AF284591AC9C

##### Notes

Perissinotto and Clennell (2021)

#### Cosmiomorpha

Saunders, 1852

7BF8FEBC-FD9B-57D1-A0B2-78025A6CB42F

#### Cosmiomorpha setulosa

Westwood, 1854

21322E76-466C-5C96-AA35-45714185B589

##### Notes

Zhang (2006)

#### Dicronocephalus

Hope, 1831

0E21EE97-E232-5340-9A2C-18005BC16271

#### Dicronocephalus wallichi

Hope, 1831

5FF260A4-B524-543C-9216-795499FFFE49

##### Notes

Zhang (2006)

#### Euselates

Thomson, 1880

692F1E75-52A9-530D-AAB5-A4978F0967F6

#### Euselates magna

Thomson, 1880

0022FAC8-F990-5F08-AD8D-6C2C83C8B1A5

##### Notes

Perissinotto and Clennell (2021)

#### Gametis

Burmeister, 1842

3B5600E3-223A-526B-B49C-483CC699D011

#### Gametis bealiae

(Gory & Percheron, 1833)

4E4DDF1D-59C8-5632-B809-3B489CD1EF23

##### Notes

Perissinotto and Clennell (2021)

#### Gametis jucunda

(Faldermann, 1835)

DF9F929B-EF17-55A4-BFE8-41DC3E2ED055

##### Notes

Perissinotto and Clennell (2021)

#### Glycyphana

Burmeister, 1842

7C818D31-77C3-5F92-AEB3-F28BBBB38108

#### Glycyphana horsfieldi

(Hope, 1831)

D08CD7E0-C4E6-5C72-87E0-8152B58056FB

##### Notes

Perissinotto and Clennell (2021)

#### Glycyphana laotica

Mikšić, 1968

14E59392-8EE4-526C-8F13-C9F1F93D5A3B

##### Notes

Perissinotto and Clennell (2021)

#### Glycyphana nicobarica

Janson, 1877

63EB30FF-287B-5489-BD09-7587DD309D00

##### Notes

DSPA (2022)

#### Protaetia

Burmeister, 1842

3E1F520A-E12F-52CA-A9CB-22673B3B125B

#### Protaetia cathaica

(Bates, 1890)

97D222D3-74C6-5EF8-B725-5AA610DE8676

##### Notes

DSPA (2022)

#### Protaetia fusca

(Herbst, 1790)

A24D67B9-A38A-57D8-B9E7-38688664115F

##### Notes

Perissinotto and Clennell (2021)

#### Protaetia intricata

Saunders, 1852

EAFCB744-D37C-5FA8-89BD-EA654E13094A

##### Notes

Perissinotto and Clennell (2021)

#### Protaetia orientalis

(Gory & Percheron, 1833)

7B8EF7EB-C0C0-5EB5-9427-A62D58D0BE85

#### Protaetia orientalis orientalis

(Gory & Percheron, 1833)

1849A9DD-4D8B-5BC8-A428-8EA289D3B15A

##### Notes

Perissinotto and Clennell (2021)

#### Protaetia speculifera

(Swartz, 1817)

5FD0523A-CDF4-53C1-8C6E-A39F34536D3A

##### Notes

Perissinotto and Clennell (2021)

#### Thaumastopeus

Kraatz, 1885

AC75D470-56B3-5C36-B286-2EA3F7878BEA

#### Thaumastopeus shanghaicus

Poll, 1886

0CAB433B-16DA-55E0-AF2A-C0CADED02B03

##### Notes

Perissinotto and Clennell (2021)

#### Thaumastopeus nigritus

(Fröhlich, 1792)

0E459F8F-E3AC-520A-9E93-45B5E41EC08B

##### Notes

Perissinotto and Clennell (2021)

#### Chrysomelidae

DC907277-18AA-5FAC-B24A-DEB77ED04E7B

#### Aulacophora

Chevrolat, 1842

8D64D661-E4BF-5E0A-8A38-849E8AE3CA80

#### Aulacophora indica

(Gmelin, 1790)

5E7DE819-7BFA-54C4-981A-E20DBB5E5F97

##### Notes

Pun and Batalha (1997)

#### Aulacophora lewisii

Baly, 1866

BEBD6131-A4D6-5727-9C0F-F40509CB2918

##### Notes

Pun and Batalha (1997)

#### Aspidomorpha

Hope, 1840

FC0BF384-218F-5970-BDB6-1E8B19BCF6AB

#### Aspidomorpha furcata

(Thunberg, 1789)

176646B2-F0A3-5CE2-B5F7-D8A6CF01295D

##### Notes

Pun and Batalha (1997)

#### Aspidomorpha miliaris

(Fabricius, 1775)

D002B290-4694-5B9E-8F75-4ECE24FC7B83

##### Notes

Pun and Batalha (1997)

#### Brontispa

Sharp, 1904

21FC9915-6D2E-5BC3-A65F-26E82EC67A7B

#### Brontispa longissima

(Gestro, 1885)

303AB885-8EC7-546F-B216-2B1BD1EA4206

##### Notes

Mo et al. (2006)

#### Donacia

Fabricius, 1775

A162FC29-9888-5DDE-8876-4C3DBA2AEC8C

#### Donacia provost

(Fairmaire, 1885)

5B1B1F1A-EF53-553F-9963-2E3FF1098180

##### Notes

DSPA (2022)

#### Haplosomoides

Duvivier, 1890

AA243097-DBEC-5708-A9BA-1CD2FCF50FC2

#### Haplosomoides costata

(Fairmaire, 1885)

840313D0-4DAD-5899-AB4A-83F26233D4F5

##### Notes

DSPA (2022)

#### Lema

Fabricius, 1798

3179D2FE-24FE-5D35-A86D-A06240C1894C

#### Lema commelinae

Gressitt, 1942

D7B541AC-DA0A-5FC7-B7AB-9BA4334A8A51

##### Notes

DSPA (2022)

#### Lema infranigra

Pic,1924

6C268BEE-D3F7-5E44-95CD-DA66366217C7

##### Notes

DSPA (2022)

#### Lema rufotestacea

Clark, 1866

71DC67F8-8F34-5CA4-8F78-05AC8B0E618B

##### Notes

DSPA (2022)

#### Lilioceris

Reitter, 1913

D578CD56-70EE-5D8F-A16B-2CF52B92069D

#### Lilioceris egena

(Weise, 1922)

D746821E-7584-57A0-8DDB-E590C85F135E

##### Notes

Pun and Batalha (1997)

#### Monolepta

Chevrolat, 1837

99C4E300-9AA2-5945-9818-FEA926FD7EEE

#### Monolepta longitarsodies

Chujo, 1938

EC184EEA-CD43-5C71-BD8E-BCF925FD4E48

##### Notes

DSPA (2022)

#### Monolepta pallidula

(Baly, 1874)

394F0AF7-4ED2-52A2-BF62-97FB0960205D

##### Notes

Mo et al. (2006)

#### Monolepta signata

(Olivier, 1808)

431F3AD2-8131-55D2-A044-D66A52270F5E

##### Notes

Pun and Batalha (1997)

#### Nisotra

Baly, 1864

EF771A94-80D9-5910-8EBA-B390E5878D82

#### Nisotra orbiculata

(Motschulsky, 1866)

2EA67C56-62C0-5C60-AB55-FA401221DF60

##### Notes

Pun and Batalha (1997)

#### Oides

Weber, 1801

41763978-6198-52E7-B02A-280B78D631DE

#### Oides decempunctatus

(Billberg, 1808)

826547AE-808E-5E94-96E4-5C7FC69FFC72

##### Notes

Pun and Batalha (1997)

#### Ophrida

Chapuis, 1875

A9C1A091-5655-5039-9901-7C520D24BD39

#### Ophrida scaphoides

(Baly, 1865)

C771790E-DA5C-5835-96CE-83E13983A918

##### Notes

Pun and Batalha (1997)

#### Pachnephorus

Chevrolat, 1837

1EBF8FBE-8B4E-59C8-BF87-9CABFBD50FF6

#### Pachnephorus brettinghami

Baly, 1878

D6FA0F65-4305-5A49-B3AD-8779EF396ACD

##### Notes

DSPA (2022)

#### Paraluperodes

Ogloblin, 1936

1942DA3C-AF0B-550A-8A6A-DC69A6F62672

#### Paraluperodes suturalis

(Motschulsky, 1858)

B88CE8B9-C545-56CD-AF1F-2762F9434B93

##### Notes

DSPA (2022)

#### Phygasia

Baly, 1876

45880DD8-4349-5351-986F-1FD91C04F5F5

#### Phygasia ornate

Baly, 1876

FDB78F65-BBA4-5E8F-98A3-EF6C2684D73B

##### Notes

Pun and Batalha (1997)

#### Phyllotreta

Chevrolat, 1836

F6242A2E-274D-5A3A-8470-9F9D99D2EC62

#### Phyllotreta striolata

(Fabricius, 1801)

B8DF9BEB-F4B1-51F9-9BF2-B09565D7B0EE

##### Notes

Pun and Batalha (1997)

#### Podagricomela

Heikertinger, 1924

3CCAD728-3FC5-5D33-8CD9-DDF7521C9153

#### Podagricomela apicipennis

(Jacoby, 1905)

41ADE242-BF0D-5224-A9F3-AA6B69B02AC0

##### Notes

DSPA (2022)

#### Rhadinosa

Weise, 1905

46B0084F-8562-587D-8ED7-A931CEE04618

#### Rhadinosa fleutiauxia

(Baly, 1890)

57BBD5FC-13B4-556E-B607-366ACA7CF463

##### Notes

DSPA (2022)

#### Sagra

Fabricius, 1792

BD403E62-9605-5F8A-9C9A-12D6DD250496

#### Sagra femorata

(Drury, 1773)

44E3EB2B-1031-5220-9C1C-98A07A117CA0

#### Sagra femorata purpurea

Lichtenstein, 1795

58F744C6-2109-56A9-9258-A54062A141EB

##### Notes

Pun and Batalha (1997)

#### Sphenoraia

Clark, 1865

7F17B192-D71C-5032-96F9-984EBE3604BD

#### Sphenoraia nebulosa

(Gyllenhal, 1808)

61C248F7-A732-5C09-AB6D-02599940E816

##### Notes

DSPA (2022)

#### Taiwania

Spaeth, 1913

0867B160-26A7-542C-A7AE-010285E31817

#### Taiwania circumdata

(Herbst, 1799)

9C080AEB-2CF3-5EAA-B111-F1FA0FBFF9D9

##### Notes

Pun and Batalha (1997)

#### Taiwania obtusata

(Boheman, 1854)

33F93B25-7D9F-5235-A11E-8F9CAA94B79B

##### Notes

Pun and Batalha (1997)

#### Coccinellidae

43E0AD50-2810-5E42-A3AF-3128DD9F0E72

#### Afissula

Kapur, 1955

24D233F7-9F84-5137-B954-E8D96A626DF3

#### Afissula bisquadripunctata

(Gyllenhal, 1808)

B45A846F-8668-5E7E-A9CA-CE49893C0002

##### Notes

DSPA (2022)

#### Bothrocalvia

Crotch, 1874

40E2EBC0-D213-5BCF-A5FA-CDDE50ADD8BF

#### Bothrocalvia albolineata

(Gyllenhal, 1808）

F507027D-9EE1-5C55-97EE-2632006E0806

##### Notes

Pun and Batalha (1997)

#### Coccinella

Linnaeus, 1758

8D64E026-63C6-5E04-BA7B-E0138CE7DCF7

#### Coccinella transversalis

Fabricius, 1781

F233E716-4DC4-5B62-A99D-6F81655EE512

##### Notes

DSPA (2022)

#### Harmonia

Mulant, 1850

D3F9773E-004B-5B66-A411-9C8B9C528CB9

#### Harmonia octomaculata

(Fabricius, 1781)

AE57D4B2-F6A4-5719-82A8-4BE9B21C2E8D

##### Notes

DSPA (2022)

#### Harmonia sedecimnotata

(Fabricius, 1801)

DC904BED-20B4-51AC-B2F7-13E94510F78C

##### Notes

Pun and Batalha (1997)

#### Henosepilachna

Li & Cook, 1961

EA83E082-1DAB-5459-8D28-2D801D25AC18

#### Henosepilachna vigintioctopunctata

(Fabricius, 1775)

4460C27D-178D-55DE-BEE7-334DF6C4523E

##### Notes

Pun and Batalha (1997)

#### Illeis

Mulsant, 1850

6B7DA939-D850-53DD-8512-C6350CDA5E2D

#### Illeis chinensis

Iablokoff-Khnzorian, 1978

8835ACCD-5708-54B2-AC9D-DCACB550256F

##### Notes

DSPA (2022)

#### Illeis koebelei

Timberlake, 1943

EFD04B97-4EBA-5676-AE3D-7B78BC9ADDBB

##### Notes

Pun and Batalha (1997)

#### Lemnia

Mulsant, 1850

713061F2-A56A-5095-97F4-6768407F419E

#### Lemnia biplagiata

(Swartz, 1801)

CE6E5503-C5A7-5527-BC29-AE4BCFEBC2A7

##### Notes

Pun and Batalha (1997)

#### Menochilus

Timberlake, 1943

4BED4441-E110-5B48-A021-3B01F356F269

#### Menochilus sexmaculata

(Fabricius, 1781)

E7C07214-5114-5A60-85DC-43C0D3DF82A1

##### Notes

Pun and Batalha (1997)

#### Micraspis

Chevrolata, 1836

8E0B5C26-07B1-5864-8294-1AF58603DCE7

#### Micraspis discolor

(Fabricius, 1798)

B17EAFE7-5D33-5EF4-8113-0157CE9F9E90

##### Notes

Mo et al. (2006)

#### Propylaea

Mulsant, 1846

42B494B3-2B68-5C51-A33C-F0D554A63DB0

#### Propylaea japonica

(Thunberg, 1781)

77409E31-6074-5C02-82CA-B80004F51B83

##### Notes

Pun and Batalha (1997)

#### Rodolia

Mulsant, 1850

D189A725-2B6C-596D-89ED-6117534A7C28

#### Rodolia pumila

Weise, 1892

2837FB6A-46C8-51B6-96FE-4271E650542A

##### Notes

Pun and Batalha (1997)

#### Curculionidae

521ED8CB-D631-5A95-97D2-608C9EFDAAAC

#### Cyrtotrachelus

Schöenherr, 1838

10017E4E-EF77-5AAD-B487-016B2CF697EC

#### Cyrtotrachelus longimanus

(Fabricius, 1775)

B9630393-293C-5C2C-BF87-0874F966F9B0

##### Notes

Pun and Batalha (1997)

#### Desmidophorus

Schöenherr, 1837

50F0AF69-C2C9-508B-9BA2-B0883A422CEB

#### Desmidophorus hebes

(Fabricius, 1781)

F8044547-9CF3-5699-B211-6DF3CDB5A53F

##### Notes

Pun and Batalha (1997)

#### Hypomeces

Schöenherr, 1823

D0AD2370-835D-5038-B1DE-84332ADEB08B

#### Hypomeces squamosus

(Fabricius, 1792)

6091EB40-C313-52BF-956A-5E9CFDFDDDA5

##### Notes

Pun and Batalha (1997)

#### Odoiporus

Chev, 1885

C5D23702-D553-5D18-9B64-383B9F2CAB7A

#### Odoiporus longicollis

(Oliver, 1807)

998C44E7-26F9-5A99-89C2-237498107F84

##### Notes

Pun and Batalha (1997)

#### Sipalinus

Marshall, 1943

12A2625E-A678-5C7F-8CBF-7FB784E32AC0

#### Sipalinus gigas

(Fabricius, 1775)

09170B2E-6926-570F-9F4D-10B7D7D6170C

##### Notes

Pun and Batalha (1997)

#### Sitophilus

Schöenherr, 1838

07B11BE6-4FD1-5D7D-B9CD-8BA0590F3F24

#### Sitophilus oryzae

(Linnaeus, 1767)

4C827E83-0E31-5FDE-8430-ACAD591A75B9

##### Notes

Pun and Batalha (1997)

#### Dermestidae

ED1974FC-0ADC-5E9D-8C6C-FD4333ED33E1

#### Dermestes

Linnaeus, 1758

0FE3A80A-4BFF-5228-A5B1-B938255EBFC6

#### Dermestes ater

Degeer, 1774

F945EC36-DFA2-50B5-8566-A663F9AA6C50

##### Notes

Zhang (2006)

#### Dynastidae

62AB6E43-5F0A-5356-AD3C-B112F8C7136E

#### Alissonotum

Arrow, 1908

419CDCD1-F49D-51A9-9349-8CDD17A5568F

#### Alissonotum pauperum

(Burmeister, 1847)

DBCF6C7E-2B30-51EB-A401-F19A0140B1EA

##### Notes

Perissinotto and Lu (2022)

#### Allomyrina

Arrow, 1911

FE29D478-B3DD-5491-A6E3-D83175E36684

#### Allomyrina dichotoma

(Linnaeus, 1771)

E1212FED-5ED6-5791-84C7-F6D01C86A749

##### Notes

Zhang (2006)

#### Xylotrupes

Hope, 1837

25E7CD6B-F163-51DE-A9EF-9DB4B58A9681

#### Xylotrupes gideon

(Linnaeus, 1767)

FB2DBD1D-3FF3-5DA0-861F-031DBBF539BD

##### Notes

Pun and Batalha (1997)

#### Xylotrupes siamensis

Minck, 1920

068F28AE-E277-5621-92C1-6E6D11C6F411

##### Notes

Perissinotto and Lu (2022)

#### Dytiscidae

552BBEC1-53A5-5C13-ABBB-AD749DB0063D

#### Bidessus

Sharp, 1882

22B93EB6-EBA2-53B9-99B8-A0C0D5A5035F

#### Bidessus japonicus

(Sharp, 1873)

2AAD4899-6F27-5DF1-A2F3-CCEBAB9F5CC7

##### Notes

Zhang (2006)

#### Copelatus

Erichson, 1832

E00EF8EB-6AD8-5ADB-88FA-75D78A625D57

#### Copelatus bangalorensis

Vazirani, 1970

46837994-C130-5919-B596-AAB18557F9E0

##### Notes

DSPA (2022)

#### Copelatus oblitus

Sharp, 1882

0703F056-0034-53FB-9E3E-8A521F03E62C

##### Notes

Jiang et al. (2022)

#### Copelatus sociennus

Balfour-Browne, 1952

DFB0D1A2-C897-5669-AE16-1EC9B396BC57

##### Notes

Jäch and Easton (1998)

#### Copelatus subfasciatus

Zimmermann, 1919

0C0408AF-CBA2-58CD-81FE-52312257B97E

##### Notes

Jäch and Easton (1998)

#### Copelatus tenebrosus

Régimbart, 1880

F20D3CA5-46B6-5A9B-9F59-7641E4C4957E

##### Notes

Jäch and Easton (1998)

#### Cybister

Curtis, 1827

CA00D276-6DBD-56CB-B1F0-8960EA3476B1

#### Cybister suyiuatus

Erichson, 1834

E760D2E0-A6B0-59B7-A670-83F894933B23

##### Notes

Zhang (2006)

#### Cybister tripunctatus

(Olivier, 1795)

3603CFC2-6625-5C78-993A-050221444A91

#### Cybister tripunctatus lateralis

(Fabricius, 1798)

ED46C3CB-67EE-5662-A6E3-CF9EF3199E09

##### Notes

Jäch and Easton (1998)

#### Cybister ventralis

Sharp, 1882

CCEA1CC3-6A95-55C2-8DD0-E0A55D8AEA68

##### Notes

Jäch and Easton (1998)

#### Eretes

Laporte, 1833

57AB8C5E-55C9-5A08-9541-989B6096C05F

#### Eretes sticticus

(Linnaeus, 1767)

582133CF-E310-586C-AED2-140E171551DB

##### Notes

Zhang (2006)

#### Hydaticus

Leach, 1817

E76F83F0-80F4-5A4E-B8A8-EB83107ADEEE

#### Hydaticus vittatus

(Fabricius, 1775)

13379FCD-B0C0-5ABB-B37D-721C453E45C5

##### Notes

Jäch and Easton (1998)

#### Hydrovatus

Motschulsky, 1853

7FF30EF7-598C-52C5-A870-CD0F07532F83

#### Hydrovatus rufoniger

(Clark, 1863)

906AA5A5-AE13-57AE-B529-CE4D14CD520C

#### Hydrovatus rufoniger rufoniger

(Clark, 1863)

FA4E21ED-EA8D-5BE9-9EA0-CEB2006D2E51

##### Notes

Jäch and Easton (1998)

#### Hydrovatus subtilis

Sharp, 1882

7D7DE983-4C79-58EA-921C-91B87086072F

##### Notes

Jäch and Easton (1998)

#### Hyphydrus

Illiger, 1802

E820F33B-AACE-5A51-BCEB-EFDA7D61BEEF

#### Hyphydrus lyratus

Swartz, 1808

B79E40DC-0F85-5ED7-8491-1EC42C0103B0

#### Hyphydrus lyratus lyratus

Swartz, 1808

3437C787-4524-5E70-9C31-FB89EBF42D88

##### Notes

Jäch and Easton (1998)

#### Laccophilus

Leach, 1815

8C7B1199-E7E6-540A-9BD8-CCBA14A8CA40

#### Laccophilus difficilis

Sharp, 1873

FE49867B-036B-5F0B-AFD5-959CEA8CFC6C

##### Notes

Zhang (2006)

#### Laccophilus sharpi

(Régimbart, 1889)

F7CD9458-12E1-5550-8562-E415D7953397

##### Notes

Zhang (2006)

#### Leiodytes

Guignot, 1936

6D6C856D-0339-5ABE-BB23-755F2A9641A4

#### Leiodytes nicobaricus

(Redtenbacher, 1867)

8E94CC50-1968-526B-B0ED-0F59A7F3F192

##### Notes

Jäch and Easton (1998)

#### Platynectes

Régimbart,1879

ADC66029-B846-563B-898A-782A36235E67

#### Platynectes gemellatus

Stastný, 2003

8209F55B-15FB-5FF3-B0E8-567ED791620A

##### Notes

Jiang et al. (2023)

#### Platynectes dissimilis

(Sharp, 1873)

2013C63B-997B-5360-A8E2-B7935304A988

##### Notes

Jäch and Easton (1998)

#### Rhantus

Dejean, 1833

013CD97A-0ACB-5DA7-8C60-579AE57A6DDE

#### Rhantus suturalis

(Macleay, 1825)

57EE4FD4-AAAC-53B2-82AD-C8023697525C

##### Notes

Zhang (2006)

#### Elateridae

95101618-F6BB-5EE6-AF2B-3265DD2C848C

#### Agonischius

Canděze, 1863

01CC7832-524D-58CE-B2A9-2AFE1887E256

#### Agonischius obscuripes

(Gyllenhal, 1817)

6BE93493-A5A8-565E-B1DB-CC5F12DDC288

##### Notes

Pun and Batalha (1997)

#### Campsosternus

Latreille, 1834

2577A769-32CD-576E-903E-44F74A68E5B6

#### Campsosternus auratus

(Drury, 1773)

7DD87EE5-550A-51ED-9393-7FEB8DF7C11A

##### Notes

Pun and Batalha (1997)

#### Eumolpidae

6DCE9C8A-9AE1-5748-9CFA-5F2D2A045F02

#### Colasposoma

Castelnau, 1833

5C287799-3AA1-5D2E-BD7B-C3ED13E4A0A3

#### Colasposoma dauricum

(Mannerheim, 1849)

C9452BE6-B205-54BF-B9CF-35CF564C2B60

#### Colasposoma dauricum auripenne

(Motschulsky, 1860)

A6F943F1-BDC8-5DB4-8941-471AC8332601

##### Notes

Pun and Batalha (1997)

#### Colasposoma pretiosum

Baly, 1860

77626DE9-B227-57C2-9D3C-3DE2324CF622

##### Notes

Pun and Batalha (1997)

#### Cryptocephalus

Geoffrey, 1762

77FC6FE2-DAE8-5711-921B-FAA376CA6368

#### Cryptocephalus suavis

Duvivier, 1892

7551191C-A01E-5F70-AEA8-1878F0DAAD3B

##### Notes

DSPA (2022)

#### Cryptocephalus trifasciatus

Fabricius, 1787

F62318ED-8EC5-5CF9-8582-7FF7BBC51CA2

##### Notes

Pun and Batalha (1997)

#### Gyrinidae

413686E5-52FF-5DDC-B925-0DD3FED269B3

#### Patrus

Aubé, 1838

F27CAA7A-1333-5125-9FC8-28D92B97AED3

#### Patrus productus

(Régimbart, 1884)

4428FA02-B558-564B-9DA6-CA359B0F9A3E

##### Notes

Jäch and Easton (1998)

#### Haliplidae

40109A4A-7F81-5166-99F4-D3857C7718D0

#### Peltodytes

Régimbart, 1879

3B9AB75C-29ED-5F0A-9E7F-81B1BECF31B7

#### Peltodytes sinensis

(Hope, 1845)

C1FBEEB4-2395-5C93-B8A3-2366E4D57F6A

##### Notes

Zhang (2006)

#### Hydrophilidae

A4055F0D-C9FA-5B37-A634-1984ACDAB6CB

#### Chaetarthria

Stephens, 1835

8B673947-D66E-5773-B4A7-D2CEEB9F63F8

#### Chaetarthria saundersi

Orchymont, 1923

16C660FE-FE4A-5D90-9B7A-DE954D15E0B3

##### Notes

Jia et al. (2018)

#### Amphiops

Erichson, 1843

4FC9FB64-27BC-573E-8197-82594F3FF61C

#### Amphiops amplelevatus

(Jia, 1995)

545E9E5D-A570-58AE-AF68-BEE9193E44DF

##### Notes

DSPA (2022)

#### Amphiops coomani

Orchymont, 1926

E01F3B01-0603-54A3-8E6F-21E182690EE1

##### Notes

Jia (2014)

#### Amphiops globus

Erichson, 1843

EF33EDE9-0F69-5EA6-9F19-C27BD480CE8A

##### Notes

Jia (2014)

#### Amphiops mater

Sharp, 1884

E1F59AA4-B696-5FD1-96A4-2061101A740E

##### Notes

Zhang (2006)

#### Berosus

Leach, 1817

140AF7DF-6784-590B-A7EC-ED3FB646AB21

#### Berosus elongatulus

Jordan, 1894

F13FA9C6-D87A-5E33-A91C-D3668354D4D3

##### Notes

DSPA (2022)

#### Berosus incretus

Orchymont, 1937

5F4460DD-4806-520C-A8F2-A18EBAD00B3C

##### Notes

DSPA 2022

#### Berosus lewsius

Sharp, 1873

FB0D52D3-CC89-5057-BE9A-9FCA20A0462D

##### Notes

DSPA (2022)

#### Cercyon

Leach, 1817

CAFF0EB1-553C-57B6-9500-52A981FEC63F

#### Cercyon laminatus

Sharp, 1873

A3B95DAC-E0F3-5286-BBF0-10548EAE686E

##### Notes

DSPA (2022)

#### Coelostoma

Brulle, 1835

674C0B2D-CC39-5CB8-ADD1-91A67BB70C01

#### Coelostoma stultum

(Walker, 1858)

019A5787-ACCF-5F44-8CE5-82E9A84722BA

##### Notes

Jia et al. (2014)

#### Coelostoma sulcatum

Pu, 1963

E23742C9-BCFA-5757-9E01-7D908147A8D0

##### Notes

Mai et al. (2022a)

#### Dactylosternum

Wollaston, 1854

BB4CF2B4-5A73-5C64-9814-8EF81CBC369F

#### Dactylosternum abdominale

(Fabricius, 1792)

AB073B22-1FD5-5118-A0CC-E026B9B5EDBE

##### Notes

Mai et al. (2022b)

#### Dactylosternum corbetti

Balfour-Browne, 1942

AFB2EC3C-C2E3-5C71-8CBD-9FCAE0C1A843

##### Notes

Mai et al. (2022b)

#### Enochrus

Thomson, 1859

250DDCD5-F056-573A-8AD0-79BAE098687D

#### Enochrus esuriens

(Walker, 1858)

FFE8E8E0-B34E-5552-9585-54597A83E880

##### Notes

DSPA (2022)

#### Enochrus flavicans

(Regimbart, 1903)

8B5AB11F-368D-5CFD-865D-308B4484A8F0

##### Notes

DSPA (2022)

#### Helochares

Mulsant, 1844

3F21B074-938E-5E88-AB21-E13045852406

#### Helochares atropiceus

Régimbart, 1903

D19294D5-BE58-599B-B43E-EDF1E73DD474

##### Notes

DSPA (2022)

#### Helochares densus

Sharp, 1890

1429E204-1A37-59AF-A67A-20B1577F1E02

##### Notes

Yang et al. (2021)

#### Helochares fuligonosus

Orchymont, 1932

22FF9B48-A594-50F2-8E64-9A0BE071B715

##### Notes

DSPA (2022)

#### Helochares neglectus

(Hope, 1854)

A2CB7477-04AB-5FC1-AE47-C76EE2FB589A

##### Notes

DSPA (2022)

#### Helochares pallens

(MacLeay, 1825)

D9175320-7EA4-5D63-A27B-E1636DD6E18D

##### Notes

DSPA (2022)

#### Paracymus

Thomson, 1867

13A87E34-7CBE-5453-8DED-86C5504F515A

#### Paracymus orientalis

Orchymont, 1926

FA539236-10E4-5337-BC5C-5AB36C95E9B9

##### Notes

DSPA (2022)

#### Sternolophus

Solier, 1834

00751120-F2CE-581A-82DF-240916AE7E49

#### Sternolophus inconspicuus

(Nietner, 1856)

59B96F1D-A07A-5B2F-A652-BC179AFBC019

##### Notes

Przewoźny (2017)

#### Sternolophus rufipes

(Fabricius, 1792)

DBA7F1CE-2B8D-5069-9EC3-946904369E04

##### Notes

Liu et al. (2021)

#### Laemophloeidae

307FA916-23D9-5D8E-A2CC-F81BFAECA30E

#### Cryptolestes

Ganglbauer, 1899

3FB67C6E-5248-5EED-BD4E-933A7DF15252

#### Cryptolestes ferrugineus

(Stephens, 1831)

F106F418-2CD2-5676-B101-43BB69F610C0

##### Notes

Zhang (2006)

#### Cryptolestes pusillus

(Schönherr, 1817)

8890C84E-7014-56AD-9E5D-D44716E92E36

##### Notes

Zhang (2006)

#### Lampyridae

6590791F-0B40-592F-8F64-8AA6158A5AF0

#### Pyrocoelia

Gorham, 1880

3B046B36-C753-5925-B3EC-1A888F91245D

#### Pyrocoelia analis

(Fabricius, 1801)

421AAB73-B7C4-5D1E-8953-4681AAAE4F11

##### Notes

Pun and Batalha (1997)

#### Lucanidae

70CC2335-F873-5C66-8E2C-4004C3E1D291

#### Prosopocoilus

Westwood, 1845

C9DFC886-4A52-529D-A166-74911E32F994

#### Prosopocoilus biplagiatus

(Westwood, 1855)

46519722-D2A7-5708-B0EE-49FB20125E76

##### Notes

Pun and Batalha (1997)

#### Melolonthidae

104CF0B6-D806-54EE-9F7F-CBB563AC28D7

#### Apogonia

Kirby, 1819

8575ABB3-442A-557F-A0CF-BC9767405916

#### Apogonia cribricollis

Burmeister, 1855

6391E5F2-95A5-50F7-9E6C-F5F6A05A3365

##### Notes

Pun and Batalha (1997)

#### Holotrichia

Hope, 1837

48D8907E-8ADD-59E6-B25D-C7B91504A2CA

#### Holotrichia geilenkenseri

Brenske, 1902

B5CD62D4-79F1-58B1-889B-7EB257C26F74

##### Notes

Easton (1992)

#### Holotrichia sinensis

Hope, 1842

135AD025-F66D-50A8-8044-9CB26C007F49

##### Notes

DSPA (2022)

#### Meloidae

C5F38D5C-6290-55DA-95E7-DDD519321D0A

#### Epicauta

Dejean, 1834

92E88991-774B-5C6C-BAA9-E98773469D7B

#### Epicauta tibialis

Waterhouse, 1871

64FA3915-2A8B-58DB-95E8-5A0A4E719660

##### Notes

Pun and Batalha (1997)

#### Mylabris

Fabricius, 1775

5687A930-C65E-5464-8DCD-FF77746DC14E

#### Mylabris phalerata

(Pallas, 1782)

549AEA08-63C5-5708-9D8C-702C686FB359

##### Notes

Pun and Batalha (1997)

#### Noteridae

E7E80FE5-6B66-564C-A882-E086DA477E87

#### Noterus

Clairvlle, 1806

0E14D169-8AAE-5564-ADE2-ABEFBA50B60D

#### Noterus japonicus

Sharp, 1873

AF53A972-D5E1-5866-A6C4-756F03BB7673

##### Notes

Zhang (2006)

#### Scarabaeidae

21AD76C0-3DC7-5823-99F8-CEF292614AC2

#### Adoretus

Dejean, 1833

6ED79F83-05C3-5B8E-8AA6-561DEF5FCF59

#### Adoretus convexus

Burmeister, 1855

A1260D8B-B2F4-5A79-894D-6501FE08BD76

##### Notes

Perissinotto and Lu (2022)

#### Adoretus sinicus

Burmeister, 1855

847BD2D1-37D7-588A-B272-DF626CAD90FC

##### Notes

Perissinotto and Lu (2022)

#### Anomala

Samouelle, 1819

009E4D79-1039-5F8F-BD21-03F17DA63E7C

#### Anomala antiqua

(Gyllenhal, 1817)

7DE5E36E-6661-52D5-96D4-9EA2785C76E7

##### Notes

Perissinotto and Lu (2022)

#### Anomala corpuleata

Motschulsky, 1853

8C906E5C-B95F-566F-A3A3-2FEC794B120E

##### Notes

Zhang (2006)

#### Anomala cupripes

(Hope, 1839)

B87370A0-1B86-56CB-911F-18EC954A27DB

##### Notes

Pun and Batalha (1997)

#### Anomala rubripes

Lin, 1996

1652B22F-316D-5804-8ABF-B9984E84AE4F

#### Anomala rubripes rubripes

Lin, 1996

50A81BF4-5983-5972-BFC5-0A7842ECA7A8

##### Notes

Perissinotto and Lu (2022)

#### Anomala russiventris

Fairmaire, 1893

3F5743EB-88CC-553C-94CF-8A40C418B4B8

##### Notes

Perissinotto and Lu (2022)

#### Anomala sulcipennis

(Faldermann, 1835)

134E06F4-AD51-5B4C-B4F8-5BD55B65DFC3

##### Notes

Perissinotto and Lu (2022)

#### Anomala virens

Linnaeus, 1996

FFED44BB-D096-5120-AB6E-DCFCE55BBB41

##### Notes

DSPA (2022)

#### Mimela

Kirby, 1823

04D70591-BC90-5A80-AFE2-C42783A9C462

#### Mimela chinensis

Kirby, 1823

84E2DC0A-D477-5585-A071-E037825D04F4

##### Notes

Perissinotto and Lu (2022)

#### Popillia

Dejean, 1821

268B4F65-F4B8-53A9-9BB9-2BCCA23FD02A

#### Popillia dilutipennis

Fairmaire, 1888

9B197833-60EA-52E4-A740-B4C837249540

##### Notes

Perissinotto and Lu (2022)

#### Popillia histeroidea

(Gyllenhal, 1817)

08F73D88-9D10-54AF-9ADC-0E8708080084

##### Notes

Pun and Batalha (1997)

#### Popillia indigonacea

Motschulsky, 1853

6A0AC410-D3C1-5575-BEAC-16CE6D22D6C5

##### Notes

Zhang (2006)

#### Popillia livida

Linnaeus, 1988

EFEDD40C-5688-558A-9B8F-582BB59E443B

##### Notes

Pun and Batalha (1997)

#### Popillia mutans

Newman, 1838

D3B64939-ACB6-5119-AE9E-9411117E3502

##### Notes

Perissinotto and Lu (2022)

#### Pseudosinghala

Heller, 1891

5A6E79D9-97AE-5A46-8866-BF91E278881C

#### Pseudosinghala dalmanni

(Gyllenhal, 1817)

88F2FF35-1C4A-5A21-A381-3FCAB4A08232

##### Notes

Perissinotto and Lu (2022)

#### Catharsius

Hope, 1837

151EC86A-5E51-591F-8721-181E658A1315

#### Catharsius molossus

(Linnaeus, 1758)

704A6E85-406B-5251-8418-8447D750B74E

##### Notes

Zhang (2006)

#### Staphylinidae

82621253-CDCA-5E37-A120-4E896CA8C2CA

#### Anotylus

Bulyĉeva, 1955

5CF629D2-9F3E-56E2-8351-4E68E02D57DD

#### Anotylus tetrocarinatus

(Block, 1799)

ED19B38E-7698-58F1-A1C4-7AC96EA8320B

##### Notes

Easton (1992)

#### Paederus

Fabricius, 1775

805DA974-D45B-5D0E-A80E-2BD77FF5C78E

#### Paederus fuscipes

Curtis, 1826

2F7C63A9-CFF3-5A6C-9FB9-30CCB3F75016

##### Notes

DSPA (2022)

#### Philonthus

Stephens, 1829

944541F6-796B-51D4-96A3-F0FE8A530243

#### Philonthus donckieri

Bemhuaer, 1915

5DAA1079-3B7D-5A00-891E-2886E5FFA1CA

##### Notes

Ades and Kendrick (2004)

#### Philonthus industanus

Fauvel, 1903

5C45DFD9-7D6E-55E6-9699-BDC1E2BE79E6

##### Notes

Ades and Kendrick (2004)

#### Philonthus longicornis

Stephens, 1832

1F6D7209-43BF-5CFD-8EE2-AA66D4B047C5

##### Notes

Ades and Kendrick (2004)

#### Phucobius

Sharp, 1874

C536BC54-75BE-5334-8D36-7178DCABA1E1

#### Phucobius simulator

Sharp, 1874

311D22E9-1CD7-570B-A224-4FA51BF4DD5D

##### Notes

Easton (1991)

#### Tenebrionidae

A91A46BD-4452-5A50-ADCD-660E6B6A893B

#### Gonocephalum

Solier, 1834

00555A33-C83D-5551-8ED4-62DFE2A21383

#### Gonocephalum subspinosum

(Fairmaire, 1894)

7E2E9C05-BD2F-5D26-AEB7-587E29DC6500

##### Notes

DSPA (2022)

#### Strongylium

Kirby, 1819

77FA0EB8-0DDD-523B-A20F-7035D715941D

#### Strongylium erythrocephalum

(Fabricius, 1801)

E9E279FF-C771-562E-84EB-91A0542D14EB

##### Notes

Pun and Batalha (1997)

#### Tenebrio

Linnaeus, 1758

A7E28927-1F0E-5505-AEE3-B101CCAE192B

#### Tenebrio molitor

Linnaeus, 1758

69016800-3357-5816-BD66-E2055258BF86

##### Notes

Pun and Batalha (1997)

#### Tribolium

Macleay, 1825

A4513055-8C11-51C6-AB52-64C6D3B165B7

#### Tribolium castaneum

(Herbst, 1797)

3E99A7AA-6B33-590E-A287-754A743AB108

##### Notes

Pun and Batalha (1997)

#### Trogossitidae

62FECD51-4AFB-585A-8F3B-29369BA56933

#### Tenebroides

Piller & Mitterpacher, 1783

18AEE5CA-FD40-53D3-9EAB-B671C6A4C98C

#### Tenebroides mauritanicus

(Linnaeus, 1758)

E7CBCC89-DE7C-536E-BDA0-BAF3DD0AC7B8

##### Notes

Zhang (2006)

#### Vesperidae

55D1B42B-888B-51B4-BAAE-88C81C153136

#### Philus

Saunders, 1853

1FF4C027-036D-5497-97BE-465531A7441E

#### Philus antennatus

(Gyllenhal, 1817)

A3201B55-A73B-5889-ABDC-57FF0098F817

##### Notes

Lin et al. (2021)

#### Philus pallescens

Bates, 1866

799D3081-58BA-55AA-AB79-1647478DF01D

#### Philus pallescens pallescens

Bates, 1866

D32697E0-A38F-5088-BA9F-3A7DB3EF4E8C

##### Notes

Lin et al. (2021)

#### Hymenoptera

F48A1976-40C6-5668-B25E-1A5460A8D620

#### Aphelinidae

B49ABE50-171D-578E-92AA-6436A7914D48

#### Encarsia

Förster, 1878

8CB5D68C-937E-5CA9-90BE-206E57A3E724

#### Encarsia smithi

(Silvestri, 1926)

B9797B04-17D6-5150-9A9A-E28687492CA1

##### Notes

Heraty et al. (2007)

#### Encarsia strenua

(Silvestri, 1927)

DEA994D4-AB10-5725-B929-45788B7C92E6

##### Notes

Heraty et al. (2007)

#### Apidae

361044FC-FBCD-55B7-9820-D180B768F891

#### Amegilla

Friese, 1897

B0786B65-C076-5534-A425-A223A3744102

#### Amegilla andresi

(Friese, 1914)

B779DFEC-2FC0-5B8D-BA57-84A673269474

##### Notes

Pun and Batalha (1997)

#### Apis

Linnaeus, 1758

452EA10E-8D38-571A-B4B3-EBEE20AFD7D9

#### Apis cerana

Fabricius, 1793

BF897384-4D38-50CF-8898-3916C187F332

##### Notes

DSPA (2022)

#### Argidae

C56B4F1B-BCBA-5A1D-90E5-C1570D78A79D

#### Cimbex

Olivier, 1791

D9274438-FA9E-5FE9-A1B4-F3CA735E70AD

#### Cimbex femoratus

(Linnaeus, 1758)

42CE4F9E-47DD-57C5-BB22-B38DDFDB9793

##### Notes

DSPA (2022)

#### Braconidae

8E4A798B-0039-53FF-85C3-E0AACAAB16EB

#### Euagathis

Szépligeti, 1900

D85BA5BC-6569-5234-891C-51C44AAEDB88

#### Euagathis forticarinata

(Cameron, 1899)

FB4E69A9-0A2A-50CC-9B34-9421F16F7296

##### Notes

Achterberg et al. (2014)

#### Chalcididae

251E0E8B-7939-551E-BC50-3CFC8AC18872

#### Brachymeria

Westwood, 1829

60120A53-3B27-5E8E-96D2-665D9CD1A1DB

#### Brachymeria lasus

(Walker, 1841)

060D3EB3-4DD5-5209-8121-8AB2A4ADEC13

##### Notes

Pun and Batalha (1997)

#### Cimbicidae

95DE580C-2179-5C9F-ACA6-A21CD3A5E55B

#### Agenocimbe

Rohwer, 1910

B0BDDD19-0C71-54BF-B766-52AEFE7E0443

#### Agenocimbe jucunda

(Mocsary, 1896)

37276F3C-80CC-520A-908B-C86BF2B847F8

##### Notes

Pun and Batalha (1997)

#### Agenocimbe maculatus

(Marlatt, 1898)

1ACAC96A-9EF6-558C-B6AC-E3F1BFEC012B

##### Notes

Pun and Batalha (1997)

#### Dryinidae

EB4D3C5F-3FB5-50E0-88A2-B75B6D570117

#### Gonatopus

Ljungh, 1810

2DBD5DF3-BB9A-5C5F-A7FB-7455E824D316

#### Gonatopus nearcticus

(Fenton, 1927)

F3417C77-8B35-5E80-BE33-DCD7D711CF94

##### Notes

Barthélémy and Olmi (2019)

#### Eulophidae

B00D33FF-6048-5BC2-9FFB-EF9A25AE923E

#### Pediobius

Walker, 1846

C8DB5224-BE8F-5559-B232-3DD010EB4BCB

#### Pediobius maximus

(Girault, 1919)

4D3ADCC5-4235-586F-822C-2A44F562529D

##### Notes

Gumovsky (2001)

#### Eumenidae

7D0B1B96-766F-5A81-B444-93200E34907A

#### Antepipona

Saussure, 1855

90CB9B39-E65D-5ACD-81B5-C1D9CC56B8AA

#### Antepipona biguttata

(Fabricius, 1787)

A0D4038D-6968-56A7-B283-4E9D99CFEA41

##### Notes

DSPA (2022)

#### Phimenes

Giordani Soika, 1992

2FF6B100-4B75-57BB-86E7-8E4C1D67D2D8

#### Phimenes flavopictus

Blanchard, 1845

134C5A5B-4562-56F4-98C9-4FC7A2EBD840

##### Notes

Pun and Batalha (1997)

#### Evaniidae

6B52903E-92D8-5B1F-B4C8-DFB9C651B9A8

#### Evania

Fabricius, 1775

249378D5-0475-5AFC-9CE6-AAFA827DC0F8

#### Evania appendigaster

(Linnaeus, 1758)

8BB3039A-73D5-5F9F-A255-D1665F4DDC0E

##### Notes

Pun and Batalha (1997)

#### Formicidae

2C89A0E1-0D63-56AA-975F-A11332FF243C

#### Acropyga

Roger, 1862

B5482184-D4CD-5A9B-A3DD-30E9B19B6619

#### Acropyga acutiventris

Roger, 1862

DF51987D-7C98-53FC-BBF4-286E225FA900

##### Notes

MBD (2022)

#### Acropyga sauteri

Forel,1912

C187F040-15FA-51F6-9052-B5ADBD5EA00E

##### Notes

ACC (2023)

#### Anochetus

Mayr, 1861

F39D9D88-E855-51B0-8303-5A0A7922740C

#### Anochetus risii

Forel, 1900

6110F5F9-E001-5DB8-9DC2-04D53E2E4C9F

##### Notes

MBD (2022)

#### Anoplolepis

Santschi, 1914

0E05FB82-9C08-5D89-9C38-0E6CE4632EE8

#### Anoplolepis gracilipes

(Jerdon, 1851)

9DE05861-F3F4-5001-AC46-501C11E6F227

##### Notes

MBD (2022)

#### Brachyponera

Emery, 1900

AB4DAF02-C429-5743-9A6B-360643DF4138

#### Brachyponera obscurans

(Walker, 1859)

B1CC82DF-2B07-5530-80B0-CE8888906B8F

##### Notes

MBD (2022)

#### Brachyponera luteipes

(Mayr, 1862)

96CBB0E6-71D0-5D3E-9763-1E826F43FA5C

##### Notes

ACC (2023)

#### Brachyponera patagonicus

Mayr, 1868

291A39AF-D270-5C50-A16C-9E0E516B405D

##### Notes

Brassard et al. (2021)

#### Buniapone

Schmidt & Shattuck, 2014

C08E74BC-46C7-5152-9955-4401D4ED666F

#### Buniapone amblyops

(Emery, 1887)

1F115A32-35B0-5812-A88A-B21C186D906B

##### Notes

Brassard et al. (2020)

#### Bothroponera

Mayr, 1862

C05E8EA1-CDAC-595F-8568-975A7DC463EF

#### Bothroponera rubiginosa

(Emery, 1889)

21F5C7F4-DC54-5DF5-B6A9-A4FA906B7109

##### Notes

ACC (2023)

#### Camponotus

Mayr, 1899

72E8C563-DEBF-56C8-8C26-D1AA780EC50A

#### Camponotus albosparsus

Bingham, 1903

D0284DE2-69BA-5ACB-A62F-D130D1923E37

##### Notes

Leong et al. (2017)

#### Camponotus carin

Emery, 1889

8A1333D7-4F24-5C5F-9851-42B769120D50

##### Notes

Brassard et al. (2021)

#### Camponotus irritans

(Smith, 1857)

3DC2CAC9-DB53-5E2D-A45D-5DBC59BC3F27

##### Notes

Brassard et al. (2021)

#### Camponotus lighti

Wheeler, 1927

D5807F2D-8A0C-50DF-9B11-53078B04A5A6

##### Notes

MBD (2022)

#### Camponotus mimus

Wang & Wu, 1994

004B44EF-93F1-55AD-B719-0762DC3C34F5

##### Notes

DSPA (2022)

#### Camponotus mitis

(Smith, 1858)

F03698D0-2FC1-545F-B464-118718F2BE09

##### Notes

MBD (2022)

#### Camponotus nicobarensis

(Mayr, 1865)

180F67CC-03C4-5B32-97D8-633E3EDD217E

##### Notes

MBD (2022)

#### Camponotus parius

(Emery, 1889)

D46098AE-44B3-545C-8105-299B2CA18DCB

##### Notes

MBD (2022)

#### Camponotus tokioensis

Ito, 1912

1E37DE76-DEE6-5291-8731-92BB89FDEE02

##### Notes

DSPA (2022)

#### Camponotus variegatus

(Smith, 1858)

0A0DCB7D-19B1-5AF6-A756-08F2084EE9D8

#### Camponotus variegatus dulcis

Dalla Torre, 1893

5D736AAF-E131-571A-AC1D-FCF6FB437467

##### Notes

MBD (2022)

#### Camponotus variegatus proles

Emery, 1925

659C0BAF-353D-5647-AB7C-ACB28D68C1DB

##### Notes

MBD (2022)

#### Camponotus vitiosus

(Smith, 1874)

EE79E45E-5623-5808-8104-F237BC919EDA

##### Notes

MBD (2022)

#### Carebara

Westwood, 1840

22AABE83-A96D-582C-8AFB-6E109F214371

#### Carebara affinis

(Jerdon, 1851)

B5EB8B7D-3B9D-56E6-B79A-2BCE1CABD378

##### Notes

Brassard et al. (2021)

#### Carebara capreola

(Wheeler, 1851)

8784AB47-BF98-50F4-AD88-9A002963F84B

##### Notes

ACC (2023)

#### Carebara diversa

(Jerdon, 1920)

DEC6AA77-1458-52EB-BAE1-30496A5E1204

##### Notes

ACC (2023)

#### Carebara laotina

(Santschi, 1920)

645DDE7B-C397-5BA2-803B-C9AC52BE18EE

##### Notes

MBD (2022)

#### Carebara melasolena

Zhou & Zheng, 1997

1FFFAB09-1811-5F9B-8069-9E22A6E08C51

##### Notes

Leong et al. (2017)

#### Carebara sangi

(Eguchi & Bui, 2007)

DF4CFCAA-668D-5B4A-BDCF-2EEEDA183220

##### Notes

Brassard et al. (2021)

#### Carebara zengchengensis

(Zhou, Zhao & Jia, 2006)

A759215E-0AE7-5D36-ACF4-1FF9B1452FB5

##### Notes

MBD (2022)

#### Cardiocondyla

Emery, 1869

AF30C50B-B501-5621-A137-7EC5C8CFCF0A

#### Cardiocondyla minutior

Forel, 1899

C6CBDFD1-676B-55CC-B711-681C7E4102E1

##### Notes

MBD (2022)

#### Chronoxenus

Santschi, 1919

917EDCC5-23C4-5D4A-9B65-7BB9DB455643

#### Chronoxenus dalyi

(Forel, 1895)

66BA9A10-3898-58A4-B458-A21C123B63AD

##### Notes

ACC (2023)

#### Chronoxenus walshi

(Forel, 1895)

1387B5AB-6B9D-5E27-BA30-ED9AAB515997

##### Notes

ACC (2023)

#### Chronoxenus wroughtonil

(Forel, 1895)

9CC34583-FDB3-521A-B6CA-41DF28F2B3C1

#### Chronoxenus wroughtonil formosensis

(Forel, 1913)

F696CA62-F861-57E4-BD92-164B4579A610

##### Notes

ACC (2023)

#### Crematogaster

Lund, 1831

5F065726-736B-5039-816E-CB10DF7DB288

#### Crematogaster binghamii

Forel, 1904

A86CF257-0586-5AA9-947E-931EF802E61D

##### Notes

Leong (2017)

#### Crematogaster biroi

Mayr, 1897

4D2E76C8-8EE4-5B72-9B16-497A1D649AB1

##### Notes

ACC (2023)

#### Crematogaster dohrni

Mayr, 1879

33104171-7112-5853-A44F-D0226217637E

##### Notes

Leong et al. (2017)

#### Crematogaster ferrarii

Emery, 1888

ACD456D3-BF18-53A8-A788-146B936EDD70

##### Notes

MBD (2022)

#### Crematogaster macaoensis

Wheeler, 1928

4412A9FE-8A65-5950-8DCF-A333DF7E367E

##### Notes

Leong et al. (2017)

#### Crematogaster matsumurai

Forel, 1901

F718DD65-C380-531E-B196-4B0BE6F55405

##### Notes

Leong et al. (2017)

#### Crematogaster artifex

Mayr, 1879

734ADDBE-D3B0-5BA4-B939-9B751C4495FC

##### Notes

Huang and Zhou (2006)

#### Crematogaster quadriruga

Forel, 1911

19D6D186-CBC4-5A9F-8CA3-DC7DC3C00FD4

##### Notes

MBD (2022)

#### Crematogaster rogenhoferi

Mayr, 1879

82DE3CB4-2DF2-5F4E-A577-3DE499D27BB0

##### Notes

MBD (2022)

#### Diacamma

Mayr, 1862

3912E781-6D21-534F-B0CF-383154B914D7

#### Diacamma rugosum

(Le Guillou, 1841)

8F9A3470-DBAA-57D1-9434-6E290C6AA0A1

#### Diacamma rugosum anceps

Emery, 1896

4199DD88-FB79-5FB9-AD85-ACF6636A58E2

##### Notes

ACC (2023)

#### Dilobocondyla

Santschi, 1910

D43BD6CD-DEB5-557E-B703-DCDF9969ECF9

#### Dilobocondyla propotriangulata

Bharti & Kumar, 2013

F3646348-8579-59F9-AB0C-90000F3D7A6B

##### Notes

Brassard et al. (2021)

#### Dolichoderus

Mayr, 1855

3B5EEC42-5CF7-5037-AF71-AC9DD1827F21

#### Dolichoderus sibiricus

Emery, 1889

A2CACBA1-143B-5736-ACF5-293D820E13F2

##### Notes

Brassard et al. (2021)

#### Dolichoderus taprobanae

(Smith, 1858)

6D0C5AF0-D38B-5664-910A-02BBAC7FE9A4

##### Notes

ACC (2023)

#### Ectomomyrmex

Mayr, 1867

A89DADBF-93B5-5111-95EF-185C6CF108CE

#### Ectomomyrmex annamitus

(André, 1892)

6D7E950A-C812-524D-96AC-750765734076

##### Notes

Brassard et al. (2021)

#### Ectomomyrmex astutus

(Forel, 1886)

E5D27582-BFE9-5A7F-B12B-E93108573F6A

##### Notes

ACC (2023)

#### Ectomomyrmex leeuwenhoeki

(Forel, 1886)

419132E4-0222-5E03-B0A7-178FD73EB4A8

##### Notes

MBD (2022)

#### Euponera

Forel, 1891

64192202-E262-5C4E-AC80-769D8B1F2B28

#### Euponera pilosior

Wheeler, 1928

DAF06B3F-C0D5-5F90-9AB7-CF249167CCBF

##### Notes

MBD (2022)

#### Euponera sharpi

(Forel, 1901)

F2CADCF1-DE61-5175-A07A-9477557B4C27

##### Notes

ACC (2023)

#### Formica

Linnaeus, 1758

D57B674B-167A-5075-888B-C79F62418FB6

#### Formica exsecta

Nylander, 1846

E3843529-3D93-543D-A067-8416BAD75134

##### Notes

ACC (2023)

#### Formica fusca

Linnaeus, 1758

D2A273C1-043F-5949-AC2B-3D4F0E5EB52E

##### Notes

Wheeler (1930)

#### Gesomyrmex

Mayr, 1868

D6E3D460-D6F4-5141-A808-266BBFE04EA1

#### Gesomyrmex howardi

Wheeler, 1921

EE17F407-D9E9-5F15-9546-ECCEDED37720

##### Notes

Brassard et al. (2021)

#### Harpegnathos

Jerdon, 1851

939A5239-6B85-58B1-ACC7-AA1CDDFDA013

#### Harpegnathos venator

(Smith, 1858)

6FC720BB-BC48-5B80-A2AA-B3675AD251DD

#### Harpegnathos venator rugosus

(Mayr, 1862)

5A808562-00FE-55B6-A25A-4703AE71D1F8

##### Notes

MBD (2022)

#### Hypoponera

Santschi, 1938

48F0CCC6-BA09-5F8D-8C4C-541B798544F9

#### Hypoponera exoecata

(Wheeler, 1928)

4F05EA61-758B-5FBB-A979-2C360938221A

##### Notes

MBD (2022)

#### Leptanilla

Emery, 1870

CFC628CB-8EC8-5641-B033-C38116DB73A6

#### Leptanilla macauensis

Leong, Yamane & Guénard, 2018

2DCDF8C3-FF29-555B-A8AB-C8C1CCF6064A

##### Notes

MBD (2022)

#### Leptogenys

Roger, (1861)

F185BF78-E57C-5D5C-88C4-85BDBB37554F

#### Leptogenys chinensis

(Mayr, 1870)

F11553DB-E2B0-5DAE-97ED-42959B55DE93

##### Notes

MBD (2022)

#### Leptogenys peuqueti

(André, 1887)

0A07D869-3D47-5204-BD9E-72C3B4350C6B

##### Notes

MBD (2022)

#### Lepisiota

(Mayr, 1861)

74D1FCB3-BC2F-5465-9E94-E84281E182B0

#### Lepisiota rothneyi

(Forel, 1894)

AB0A497C-8D7D-5F6A-A844-79030E68519B

#### Lepisiota rothneyi watsonii

(Forel, 1894)

8AB340EB-EC61-554E-B51E-CEF52D49F47B

##### Notes

MBD (2022)

#### Mayriella

Forel, 1902

CBEDFFBB-7F17-511B-9F30-5CD7E91CF4A1

#### Mayriella granulata

Dlussky & Radchenko, 1990

7275FF05-DAD2-5706-A501-96A7868D9ED9

##### Notes

Brassard et al. (2021)

#### Meranoplus

Smith, 1853

D8818A6F-8904-5F1B-8EF7-11ABE69CC7DF

#### Meranoplus bicolor

Guérin-Méneville, 1844

60FBFE41-CA80-571C-A4D0-F1A7A9C35827

##### Notes

Leong (2017)

#### Monomorium

Mayr, 1855

FE490909-6935-526F-A4A6-9B77719FA474

#### Monomorium chinense

Santschi, 1925

186E89EC-6105-5AB6-BA20-A09680E71899

##### Notes

DSPA (2022)

#### Monomorium intrudens

Smith, 1874

F23360AB-1569-550C-95CC-031D697B9E2A

##### Notes

Brassard et al. (2021)

#### Monomorium floricola

(Jerdon, 1851)

886693F9-4325-5935-BCDC-79A553191AD8

##### Notes

MBD (2022)

#### Monomorium pharaonis

(Linnaeus, 1758)

797E81CB-E934-5D90-826C-88316493AC6C

##### Notes

MBD (2022)

#### Myrmecina

Curtis, 1829

BDA34541-67D3-5F34-8BC6-ECEFCD209FA2

#### Myrmecina nomurai

Okido, Ogata & Hosoishsi, 2020

7CECB1BE-4043-536B-B28E-88B89192A032

##### Notes

Brassard et al. (2021)

#### Myrmecina sinensis

Wheeler, 1921

C0AF1F06-0B23-5D1B-BD78-002730791309

##### Notes

MBD (2022)

#### Nylanderia

Emery, 1906

1D0E6F84-8538-56BD-BD55-54F32574B182

#### Nylanderia amia

(Forel, 1913)

0D67531E-46AF-55A4-856C-85E89E961790

##### Notes

MBD (2022)

#### Nylanderia bourbonica

(Forel, 1886)

8CD1A4F9-E0A0-53C8-8EB2-C69CAF4F4D2E

##### Notes

Leong et al. (2017)

#### Nylanderia indica

(Forel, 1894)

55E07BE6-CC4D-5AB7-B51B-62C2F72F0876

##### Notes

Leong et al. (2017)

#### Nylanderia sharpii

(Forel, 1899)

E7FDB6FA-4C9A-5717-8CD6-BC96AAC451D7

##### Notes

Brassard et al. (2021)

#### Nylanderia taylori

(Forel, 1894)

0F71D18A-0298-5DAD-8C91-FDF338E3ABFA

##### Notes

Brassard et al. (2021)

#### Nylanderia vividula

(Nylander, 1846)

95FF236E-65C0-57CA-ACF2-C17B495FB109

##### Notes

ACC (2023)

#### Nylanderia yerburyi

(Forel, 1894)

5434F223-3C75-5C69-A440-CC55219ADC1A

##### Notes

MBD (2022)

#### Ochetellus

Shattuck, 1992

8388868C-1BAC-5D51-9062-006391C3321A

#### Ochetellus glaber

(Mayr, 1862)

E174A622-4D79-51FB-BC48-84A19C3DDC4D

##### Notes

MBD (2022)

#### Odontoponera

Mayr, 1862

5B0608F5-14D1-54D5-92B6-6756E71671FD

#### Odontoponera denticulate

(Smith, 1858)

5C88CAC8-6D60-5210-B697-6E9FE0F5C99D

##### Notes

MBD (2022)

#### Ooceraea

Roger, 1862

625273CA-615D-5351-AE2B-21F3AE07942F

#### Ooceraea biroi

(Forel, 1907)

81BF13EE-669E-5419-A3CE-3A43D4A975AE

##### Notes

MBD (2022)

#### Pachycondyla

Smith, 1858

EADA5C4E-81A6-5ACB-890A-A170E8223B83

#### Pachycondyla luteipes

(Mayr, 1862)

29ACB4F0-290C-59C0-BAEF-35B918BAE9BD

##### Notes

Yang et al. (2004)

#### Paratrechina

Motschulsky, 1863

B909F76B-C0C1-5D1F-8BD1-A4AD6183E34E

#### Paratrechina bourbonica

(Forel, 1886)

DD6B5D64-89A4-5B3C-8164-A5B1846099FD

##### Notes

DSPA (2022)

#### Paratrechina flavipes

(Smith, 1874)

7C8176E2-4A18-5303-A75E-466762E45F88

##### Notes

DSPA (2022)

#### Paratrechina longicornis

(Latreiille, 1802)

BCC08790-EA14-5522-9E12-4991B563AAC2

##### Notes

MBD (2022)

#### Paratrechina sharpi

(Forel, 1899)

48E0CC37-3E09-5FB1-A333-9B8DC448650E

##### Notes

DSPA (2022)

#### Paraparatrechina

Donisthorpe, 1947

E52C16F0-379A-53B9-BED1-6E70D2BBCA67

#### Paraparatrechina sauteri

(Forel, 1913)

96B8EC8F-643E-5DA0-800C-805DF00D8C78

##### Notes

Leong et al. (2017)

#### Pheidole

Westwood, 1841

95CA17BD-8299-569C-9504-CD6D6997C84D

#### Pheidole elongicephala

Eguchi, 2008

9D7839DF-CE61-5522-91AB-4F09092CA9FB

##### Notes

Brassard et al. (2021)

#### Pheidole fervens

Smith,1858

C33A325C-A793-5DAC-9334-84E7D9D50723

##### Notes

MBD (2022)

#### Pheidole hongkongensis

Wheeler, 1928

B41EB23C-976E-5C51-A948-8B2D3EF3AF03

##### Notes

MBD (2022)

#### Pheidole megacephala

(Fabricius, 1793)

2A29CF5D-B92B-566F-B65E-954540A20B7B

##### Notes

MBD (2022)

#### Pheidole indica

Mayr, 1879

75D28758-82C3-5282-8080-7829E301201E

##### Notes

Leong et al. (2017)

#### Pheidole nodus

Smith, 1874

6896C246-3023-5FA3-A56E-D1690512B0AB

##### Notes

MBD (2022)

#### Pheidole ochracea

Eguchi, 2008

53782AD0-ED91-5281-B452-A9413ACFFA04

##### Notes

MBD (2022)

#### Pheidole parva

Mayr, 1865

965C0C6D-CB6D-5A3A-B5E9-BBA4A7C54770

##### Notes

MBD (2022)

#### Pheidole pieli

Santschi, 1925

5FD3884F-8689-59FF-BFDB-3FCCBD2E8F3C

##### Notes

Brassard et al. (2021)

#### Pheidole taipoana

Wheeler, 1928

EE80FDE6-D43E-50E1-AACE-87C3DCF7F5F5

##### Notes

Leong et al. (2017)

#### Pheidole tumida

Eguchi, 2008

E07CCF28-5F17-5953-88AA-A2A8E96987B7

##### Notes

MBD (2022)

#### Pheidole vulgaris

Eguchi, 2006

8A8724A9-7B0D-50E5-B5D4-ADC510BF3925

##### Notes

Brassard et al. (2021)

#### Pheidole zoceana

Santschi, 1925

CA34000C-25C9-5CEF-B3B9-033AA8880A1A

##### Notes

Brassard et al. (2021)

#### Plagiolepis

Mayr, 1861

57C6D306-7AA9-5661-A582-DB27DB49787B

#### Plagiolepis alluaudi

Emery, 1894

31D337BD-8DC1-5A8F-9D67-10113F756EED

##### Notes

Brassard et al. (2021)

#### Polyrhachis

Smith, 1857

7E778FD4-618E-5637-86B3-F31C9D1660A4

#### Polyrhachis confusa

Wong & Guénard, 2020

162EFD4E-3D7B-54D3-8FD0-E3FDE406FBD8

##### Notes

Wong and Guénard (2020)

#### Polyrhachis demangei

Santschi, 1910

996E5B17-1089-5A8F-96F3-6EE247D92A48

##### Notes

MBD (2022)

#### Polyrhachis dives

Smith, 1857

1312B5A5-80D1-589B-98F6-3013B671E25C

##### Notes

MBD (2022)

#### Polyrhachis illaudata

Walker, 1859

9E247FE5-09B4-572A-A20F-901C349EA978

##### Notes

MBD (2022)

#### Polyrhachis latona

Wheeler, 1909

6A8275EA-2323-5719-B3C8-0DC1B3BD2475

##### Notes

Wong and Guénard (2020)

#### Polyrhachis tyrannica

Smith, 1858

8392E353-E61D-5B04-BAAF-A70E8DBC5D4C

##### Notes

Wong and Guénard (2020)

#### Probolomyrmex

Mayr, 1901

F584236F-0A3B-5D83-B0C5-E9ED0D1E873E

#### Probolomyrmex dabermanii

Wheeler, 1928

2A76119A-DDEC-541D-9A4D-FA43C2587DA9

##### Notes

Brassard et al. (2021)

#### Pseudolasius

Emery, 1887

53675130-D4EF-52A4-BECA-3C393BA638A0

#### Pseudolasius risii

Forel, 1894

97E1207D-AEB8-53EC-89F4-B89062DD5BB1

##### Notes

Brassard et al. (2021)

#### Pseudoneoponera

Donisthorpe, 1943

01A169B0-25CA-5ADB-A7AC-0078CF221985

#### Pseudoneoponera rufipes

(Jerdon, 1851)

1B700925-18DB-5BB1-BD54-F98D07AAE74A

##### Notes

MBD (2022)

#### Recurvidris

Bolton, 1992

1F68F68A-C64A-5B1A-9761-B4A985E32437

#### Recurvidris recurvispinosa

(Forel, 1890)

4863EA79-53F3-5CB1-B6AE-AA1AF9C3C6CC

##### Notes

MBD (2022)

#### Rotastruma

Bolton, 1991

231E4D74-C4C7-58CD-B1D4-1D429A69A530

#### Rotastruma stenoceps

Bolton, 1991

5196FDD3-1077-51C0-969F-207823FCE191

##### Notes

Brassard et al. (2021)

#### Solenopsis

Westwood, 1841

AC536429-FEA1-5C41-9E2A-0F284A242FE4

#### Solenopsis geminata

(Fabricius, 1804)

BEF855AE-26FC-5333-A2AB-66F1945F1B61

##### Notes

MBD (2022)

#### Solenopsis invicta

Buren, 1972

9C9BC185-A2BC-5EF7-A915-8175DEF636B4

##### Notes

MBD (2022)

#### Solenopsis jacoti

Wheeler, 1923

616921E7-3949-5356-83D5-9BC1FB537B86

##### Notes

MBD (2022)

#### Strumigenys

Smith, 1860

C5771923-9698-57A5-88C3-5191C4522FCD

#### Strumigenys elegantula

Terayama & Kubota, 1989

97AEF7E5-D9E8-5587-88F7-6E866DD0D67A

##### Notes

Brassard et al. (2020)

#### Strumigenys emmae

(Emery, 1890)

1FD8AE11-A600-578F-8D9C-DA1EB8B1F098

##### Notes

MBD (2022)

#### Strumigenys exilirhina

Bolton, 2000

2E5E8AB0-8B3F-5936-99A8-04D9658A9BE4

##### Notes

MBD (2022)

#### Strumigenys feae

Emery, 1895

6BFEFEF0-806D-5176-8945-D3BC4E7131A5

##### Notes

Brassard et al. (2020)

#### Strumigenys membranifera

Emery, 1869

88BE9730-2298-51B4-9495-EBE41195CB89

##### Notes

Brassard et al. (2020)

#### Strumigenys minutula

Terayama & Kubota, 1989

9C0CCC7F-B144-538F-A368-8736822180D1

##### Notes

MBD (2022)

#### Strumigenys nepalensis

Baroni Urbani & De Andrade, 1994

8E4E8105-BB27-5279-AC2C-BB81D26273F7

##### Notes

MBD (2022)

#### Strumigenys sauteri

Forel, 1912

A8AA1D41-2EE6-5244-8F7B-A9906E752C27

##### Notes

Brassard et al. (2020)

#### Strumigenys subterranea

Brassard, Leong & Guénard, 2020

8BBF878A-09BB-5349-A715-84D3C32BC6AA

##### Notes

Brassard et al. (2020)

#### Strumigenys silvestrii

Emery, 1906

6AFE735C-57CA-5C97-A732-FCA6A28D262E

##### Notes

Wetterer et al. (2019)

#### Stigmatomma

Roger, 1859

CCC38606-BCF6-5ECA-B17F-F65352A1F7C2

#### Stigmatomma rothneyi

(Forel, 1900)

4A79C95B-D2AC-517C-AA68-19A82D6B89D9

##### Notes

Leong et al. (2017)

#### Syllophopsis

Santschi, 1915

0EE55797-88E3-5AF3-92AF-06D5271D677D

#### Syllophopsis sechellensis

(Emery, 1894)

A82B5FDE-4DD0-5B8E-B81F-70241F9949C5

##### Notes

Leong et al. (2017)

#### Tapinoma

Foerster, 1850

866F8E12-5454-5B4F-B430-B041FBDEB04B

#### Tapinoma indicum

Forel, 1895

54672C40-3F5C-554A-B7D4-F456A6DC99EB

##### Notes

MBD (2022)

#### Tapinoma melanocephalum

(Fabricius, 1793)

7BE89D86-7EFE-5FC8-8BC7-968EB2E7B1CE

##### Notes

MBD (2022)

#### Technomyrmex

Mayr, 1872

7DF7969F-1175-582B-B5D6-7411C1001EDF

#### Technomyrmex albipes

(Smith, 1861)

BE1AC317-8C3C-5488-A5AD-CA4CD1013DC5

##### Notes

Leong et al. (2017)

#### Technomyrmex antennus

Zhou, 2001

9164D126-3A3E-55F5-BBE8-EECD071942C1

##### Notes

DSPA (2022)

#### Technomyrmex brunneus

Forel, 1895

FA59664E-FFFD-5D84-961B-AA9637C9CBB5

##### Notes

MBD (2022)

#### Technomyrmex horni

Forel, 1912

565E413E-3801-5B90-A1A5-517E12AEBEBB

##### Notes

Brassard et al. (2021)

#### Tetraponera

Smith, 1852

82853867-6B31-5317-B5A0-E621EB1C1B54

#### Tetraponera allaborans

(Walker, 1859)

75412FF3-895D-51E5-8541-13D54478BF59

##### Notes

MBD (2022)

#### Tetraponera binghami

(Forel, 1902)

B83D0D2B-2C85-5828-9650-736CBB2EB1E5

##### Notes

Brassard et al. (2021)

#### Tetraponera nitida

(Smith, 1860)

3FA13E7D-9893-55FB-BE04-FC1E71D3C318

##### Notes

Brassard et al. (2021)

#### Tetramorium

Mayr, 1855

A293D050-2DD4-54B1-80EA-9259554D186D

#### Tetramorium bicarinatum

(Nylander, 1846)

9944E266-5E0C-5F68-B41B-7298A2BF624B

##### Notes

MBD (2022)

#### Tetramorium indicum

Forel, 1913

1A6ABD3E-E417-551A-A837-B69F38C4CD5F

##### Notes

MBD (2022)

#### Tetramorium insolens

(Smith, 1861)

F4A36864-3B53-51C9-854F-AC888996CBF6

##### Notes

Brassard et al. (2021)

#### Tetramorium kraepelini

Forel, 1905

78679265-BA18-5A87-9529-0E8E92821DC7

##### Notes

MBD (2022)

#### Tetramorium lanuginosum

Mayr, 1870

00987DD0-CE60-5B02-84B3-09A6D1ABDE1B

##### Notes

MBD (2022)

#### Tetramorium nipponense

Wheeler, 1928

2550F3EE-86A1-5F0F-AC8E-C8A91A7DC5A6

##### Notes

MBD (2022)

#### Tetramorium parvispinum

(Emery, 1893)

B7B23B74-FE35-52CC-856E-572F4F690DF9

##### Notes

MBD (2022)

#### Tetramorium simillimum

(Smith, 1851)

A67C50DD-E4CB-52B1-80FE-A876E66E0C16

##### Notes

MBD (2022)

#### Tetramorium tonganum

Mayr, 1870

FF6612CB-2DCA-5CAB-8D0C-19B22AC6A33D

##### Notes

Brassard et al. (2021)

#### Tetramorium tsushimae

Emery, 1925

CB732E05-E055-555D-A804-2AAF14CA49D8

##### Notes

Guenard (2011)

#### Tetramorium wroughtonii

(Forel, 1902)

4A5F04FE-5F77-53EB-88E2-C9E0B7A42D25

##### Notes

Leong et al. (2017)

#### Ichneumonidae

5951FD32-C35C-5F81-8AF6-5E286D5DD722

#### Dicamptus

Szépligeti, 1905

E6EBE560-D156-5477-9A76-AC494D7819DF

#### Dicamptus reticulatus

(Cameron, 1899)

B69DED2D-1B33-570B-854D-F3ADC513CA3B

##### Notes

DSPA (2022)

#### Gotra

Cameron, 1902

A0F80FED-5E4F-5BE7-92B3-E2591B2B463B

#### Gotra octocinctus

(Ashmead, 1906)

C1294910-AB21-55AF-8612-CE37F7FC8226

##### Notes

Pun and Batalha (1997)

#### Leptobatopsis

Ashmead, 1900

C88E6C3F-5791-5CE8-B601-7478732FFA69

#### Leptobatopsis indica

(Cameron, 1897)

D5E6FB47-7BD5-522C-8DB2-1729F7B5A865

##### Notes

Pun and Batalha (1997)

#### Xanthopimpla

Saussure, 1892

D2E825DC-3692-59AF-B67F-D186A9033147

#### Xanthopimpla pedator

(Fabricius, 1775)

7CBF6655-7998-56FF-8843-194D37A6BF61

##### Notes

Pun and Batalha (1997)

#### Pompilidae

BC2BB701-9CCE-56CB-96EA-C5450011C131

#### Leptodialepis

Haupt, 1929

4DCE1686-4E11-5F37-AB08-A8CE308F8347

#### Leptodialepis bipartilus

(Lepeletier, 1845)

C07E5227-1236-5BB2-BA78-FF262CF3F706

##### Notes

Pun and Batalha (1997)

#### Scoliidae

1A8FAE36-D8EE-510D-9495-4CB053A2A5E9

#### Campsomeriella

Betrem, 1941

2DB62321-3E5C-590A-960F-D5BF3A9325FC

#### Campsomeriella annulata

(Fabricius, 1793)

9DBEDF2F-3B76-5417-9168-816D4859209A

##### Notes

Pun and Batalha (1997)

#### Campsomeriella formosensis

(Betrem, 1928)

ED5138C7-1765-5B8B-B9D9-F3FC2B11075A

##### Notes

Pun and Batalha (1997)

#### Campsomeriella prismatica

(Smith, 1855)

4BE813DA-3E30-513A-8B6D-19E6CECF3B8E

##### Notes

Pun and Batalha (1997)

#### Megascolia

Betrem, 1964

8DFE348B-E89C-5079-BA12-DE63D2EC7DD5

#### Megascolia azurea

(Christ, 1791)

FC0AEB5E-B98C-56CD-9BAD-D51A5C4F26EB

##### Notes

Pun and Batalha (1997)

#### Scolia

Fabricius, 1775

B6C6501B-5B7F-517B-859F-2698E26B9FA8

#### Scolia quadripustulata

Fabricius, 1782

D4659EE1-1302-59B8-A78B-C6DDFD8F7CAC

##### Notes

DSPA (2022)

#### Scolia superciliaris

Saussure, 1864

90020C6B-8296-5B89-BE5F-E182CD49FFDA

##### Notes

Pun and Batalha (1997)

#### Sphecidae

03FEA460-F886-59A0-B15E-05F76949AE02

#### Ammophila

Kirby, 1798

2A77CAD3-2461-5D67-91D7-F023BC52ADE9

#### Ammophila atripes

Smith, 1852

E2362FB4-CE2E-59A6-9C1F-93979C87FB0A

#### Ammophila atripes atripes

Smith, 1852

DAC44929-EF00-51E3-B63B-7C9819B9AF93

##### Notes

Pun and Batalha (1997)

#### Chalybion

Dahlbom, 1843

55E9227A-EA90-5634-973C-17DDDAA1DFB1

#### Chalybion japonicum

(Gribodo, 1883)

099FB446-BE90-59B5-9A19-A6B33C4472AF

##### Notes

Pun and Batalha (1997)

#### Vespidae

27DAE5AB-8C36-515A-A7BB-4028C446D1BE

#### Delta

Saussure, 1855

DAA8C34B-69F4-5673-BCAA-CB55350BF7F3

#### Delta campaniforme

(Fabricius, 1775)

0BAB41D0-D6A0-520C-A03C-D620E3E9A682

#### Delta campaniforme esuriens

(Saussure, 1852)

871580D8-52B8-57AE-8D6E-91497FF3398B

##### Notes

Pun and Batalha (1997)

#### Delta petiolata

(Fabricius, 1781)

8612B270-D98E-56E0-B432-3623416ED1AE

##### Notes

Pun and Batalha (1997)

#### Labus

Saussure, 1867

4F8D5B56-ACAB-53E1-BE00-CC2F18C0C857

#### Labus lofuensis

Giordani Soika, 1973

FEA80453-DBB5-5536-9177-321A8401AFBF

##### Notes

Nguyen and Carpenter (2020)

#### Parapolybia

Saussure, 1854

FF1028AA-EA99-5B5B-9A42-179E3B4B451A

#### Parapolybia varia

(Fabricius, 1787)

D0E5A485-DE14-5E06-958A-DE18C3FC165A

##### Notes

Pun and Batalha (1997)

#### Polistes

Latreille, 1802

64BEF7B2-475B-5E69-9B05-22235E556072

#### Polistes gigas

(Kirby, 1826)

91CF567B-871D-512E-91CD-06285B32BC4F

##### Notes

Pun and Batalha (1997)

#### Polistes macaensis

(Fabricius, 1793)

D9AB5B40-5C69-5365-865B-0109172D4FE6

##### Notes

Barthélémy et al. (2014)

#### Polistes olivaceus

(Geer, 1773)

F035F901-8379-5B7C-ABDE-8BC5A2EF00BC

##### Notes

Pun and Batalha (1997)

#### Polistes sagittarius

Saussure, 1854

669D0E2C-69E6-5CBB-BE31-4C4F54550E4B

##### Notes

Pun and Batalha (1997)

#### Polistes stigma

(Fabricius, 1793)

70D6A397-B7DD-5CE2-935A-D3D862790667

##### Notes

Pun and Batalha (1997)

#### Rhynchium

Spinola, 1806

BA67A980-1213-5779-BB6D-9AB117D78C25

#### Rhynchium brunneum

(Fabricius, 1793)

3F640A8B-4964-5CE4-B62F-690513C86F27

##### Notes

Pun and Batalha (1997)

#### Rhynchium quinquecinctum

(Fabricius, 1787)

E5F81C86-C57F-52F0-B4DB-610DC01D4175

##### Notes

Pun and Batalha (1997)

#### Vespa

Linnaeus, 1758

AC8486E3-F159-5F3D-8B3A-36F38B0AF7D0

#### Vespa affinis

(Linnaeus, 1764)

51647659-CA20-5430-A893-CD9C0B06BD75

##### Notes

Pun and Batalha (1997)

#### Vespa bicolor

Fabricius, 1787

59DB8E1D-D34E-590B-8041-299226D65773

#### Vespa bicolor bicolor

Fabricius, 1787

DDF1F353-B30C-5913-B1D3-1FB8438884D8

##### Notes

Pun and Batalha (1997)

#### Vespa binghami

Buysson, 1905

7467416E-49A5-58D2-AD9B-1B2092FC56BF

#### Vespa binghami binghami

Buysson, 1905

0173F37F-8CB2-5C10-88DD-3B00AA4CA153

##### Notes

DSPA (2022)

#### Vespa tropica

Linnaeus, 1758

32E154E8-B657-55DF-8876-D01F67DE540C

##### Notes

Pun and Batalha (1997)

#### Xylocopidae

68DEADC7-BACC-5914-B951-6D14CCE08C29

#### Xylocopa

Latreille, 1802

3E7E9DA7-2785-5F9B-9B4A-1481923219F1

#### Xylocopa iridipennis

Lepeletier, 1841

6FD6BE48-13F7-5785-97AD-F448DBCC6B0B

##### Notes

Pun and Batalha (1997)

#### Xylocopa tranquabarorum

(Swederus, 1787)

F419F2B0-BF0A-5CAE-B5AB-C9D8D758EFAD

##### Notes

DSPA (2022)

#### Hemiptera

FF1C456A-47E1-5D72-B5DD-714E2636C497

#### Acanthosomatidae

FBE377FE-91B5-53F4-B8C0-FB3762F50119

#### Elasmostethus

Fieber, 1860

6BF0DF69-777F-52AA-A63C-F7BB515F5999

#### Elasmostethus nubilus

(Dallas, 1851)

E997F679-EE8E-56B2-B8C5-13D4ADA48E01

##### Notes

Pun and Batalha (1997)

#### Aleyrodidae

11D1F657-D382-5D31-BFF9-E129773F2C9C

#### Aleurocanthus

Quaintance & Baker, 1914

C3541860-B202-59A9-B8A2-C4F938ED0AB7

#### Aleurocanthus spiniferus

(Quaintance, 1903)

05EC007E-3799-57F9-A6CF-951F814FFA12

##### Notes

Pun and Batalha (1997)

#### Bemisia

Quaintance & Baker, 1914

ACB7E411-8314-5DC5-B9B4-887AE22093CE

#### Bemisia tabaci

(Gennadius, 1889)

69C5B216-D615-5797-B2A5-DAE8F9FBF4E0

##### Notes

Li et al. (2011)

#### Dialeurodes

Cockerell, 1902

4665D13C-FE57-548A-B70F-475A12D4BD8F

#### Dialeurodes citri

(Ashmead, 1885)

1F9BEAD2-9CA4-5A5D-BFE9-DED41019B5AA

##### Notes

Shahbazvar et al. (2010)

#### Trialeurodes

Cockerell, 1902

CA2AA939-F962-5020-AF8D-0E590DB6E628

#### Trialeurodes vaporariorum

(Westwood, 1856)

D3286257-CEA2-54A8-BAA4-16EB0C9B1B9A

##### Notes

Pun and Batalha (1997)

#### Alydidae

905FD5CF-06D6-541E-B489-E5ED32C81512

#### Leptocorisa

Latreille, 1829

4C208C67-C8E9-56CE-A58A-2B4C4A0137B7

#### Leptocorisa acuta

(Thunberg, 1783)

A70506E2-DFED-5454-9ABD-1175AF1F2936

##### Notes

Pun and Batalha (1997)

#### Riptortus

Stål, 1860

E301E2FF-F354-5080-B1A7-6939A1BE4AE0

#### Riptortus linearis

(Fabricius, 1775)

D894452C-3B99-5B7B-A067-3015474B3F94

##### Notes

Pun and Batalha (1997)

#### Riptortus pedestris

(Fabricius, 1775)

18CFC774-2D24-5C1A-B347-F7E92937B9EF

##### Notes

DSPA (2022)

#### Aphididae

09D037DD-3EE5-5C64-9A2F-B15D8DE17048

#### Aphis

Linnaeus, 1758

E5BBF1D2-8DAB-5865-A7BF-AF356C2C6860

#### Aphis gossypii

Glover, 1877

A9A37DC9-3A53-5972-9C34-C6CAAE3A25E0

##### Notes

Pun and Batalha (1997)

#### Aphis nerii

Fonscolombe, 1841

75630665-D25A-5000-BD5F-A826176EE765

##### Notes

Pun and Batalha (1997)

#### Formosaphis

Takahashi, 1925

E5043BC6-6EDC-5EBC-AE4A-4AC14E7710A7

#### Formosaphis micheliae

Takahashi, 1925

CA59A987-8901-5988-BE47-2E8BAFCDA6BF

##### Notes

Pun and Batalha (1997)

#### Myzus

Passerini, 1860

85603679-3D85-594E-AC96-AC3957E048C8

#### Myzus persicae

(Sulzer, 1776)

ABFEBD2F-04D7-5FDF-958A-34DB87829C2B

##### Notes

Pun and Batalha (1997)

#### Neophyllaphis

Takahashi, 1920

47D2EAFF-A4C7-556F-944C-D406BEE78742

#### Neophyllaphis podocarpi

Takahashi, 1920

7C103996-8354-5674-B32C-303F8E2B832F

##### Notes

Pun and Batalha (1997)

#### Tinocallis

Matsumura, 1919

D0CEAC6F-D1B0-5323-9ED8-8F8FA511380D

#### Tinocallis kahawaluokalai

(Kirkaldy, 1907)

D3549E20-CC8B-56D1-A20E-766C22E51E67

##### Notes

Pun and Batalha (1997)

#### Shivaphis

Das, 1918

8E1E2655-5CF1-50AB-9B00-E917B484831E

#### Shivaphis celti

Das, 1918

1099AAE3-AE46-5813-9DC7-2A3BF262CF80

##### Notes

Pun and Batalha (1997)

#### Aphrophoridae

9E8F06D7-8787-51DB-933D-53161B635FAD

#### Clovia

Stål, 1866

1113411B-525B-5F8A-A0B1-D5D534005331

#### Clovia quadrangularis

Metcalf & Horton, 1934

E0C026B1-D908-5528-8BEF-FB7F0ADC9228

##### Notes

DSPA (2022)

#### Belostomatidae

A835C8DB-C86E-5069-8C1C-EA8363D97628

#### Diplonychus

Laporte, 1833

EB9DFBD0-6F9F-5822-AD34-775267AF5179

#### Diplonychus rusticus

(Fabricius, 1781)

24E6D24E-60EC-567A-A7A2-C67B7034E487

##### Notes

Easton and Pun (1997a)

#### Lethocerus

Mayr, 1853

F47255EE-1FB9-534C-8007-14C27543F52D

#### Lethocerus indicus

(Lepeletier & Serville, 1825)

E31CABA2-979F-591F-9F02-76D0D2772887

##### Notes

Easton and Pun (1997a)

#### Carsidaridae

10E4F838-B04A-5158-A09F-DB2086A9C32B

#### Carsidara

Walker, 1869

B630B3A1-F063-5674-9F3F-B3D48AB31FE1

#### Carsidara limbata

(Enderlein, 1926)

82C6C71B-5A40-5AD7-BE14-A18D7647898C

##### Notes

Mo et al. (2006)

#### Cicadellidae

BC1C55E6-B0CC-5E50-960E-725A26C491B4

#### Cicadella

Latreille, 1817

725CEDD8-ADAF-512B-9B89-5BB623913D7B

#### Cicadella viridis

(Linnaeus, 1758)

26AADCDD-18BC-5D0A-AD83-38EC0B423ECF

##### Notes

Mo et al. (2006)

#### Cofana

Melichar, 1926

848F3440-F89E-5A81-A263-8BF6A4CFB0FC

#### Cofana spectra

(Distant, 1908)

F7823E56-0716-5889-B344-8DDD73F4A56A

##### Notes

Pun and Batalha (1997)

#### Lodiana

Nielson, 1982

A144E965-D4EB-5E8B-818B-812CE16159E3

#### Lodiana brevis

(Walker, 1851)

79FEE7C5-E4EF-510F-A4CB-E638519CAE1A

##### Notes

Pun and Batalha (1997)

#### Nephotettix

Matsumura, 1902

F4C37079-8184-5948-A6E5-CA535A34C995

#### Nephotettix virescens

(Distant, 1908)

C168312D-2F56-5BB1-87B8-39886D586578

##### Notes

Pun and Batalha (1997)

#### Petalocephala

Stål, 1854

81BE2B78-B223-577D-9DF4-16E8A77FA29F

#### Petalocephala chlorocephala

(Walker, 1851)

B46BE378-81B1-58CE-8293-9D9FBCC82604

##### Notes

Pun and Batalha (1997)

#### Typhlocyba

Germar, 1833

9D9C3E9D-D506-53F1-A41C-7F1E9F6F9814

#### Typhlocyba rosae

(Linnaeus, 1758)

7A9360C5-256B-5277-BC28-FEFB5149C5E0

##### Notes

Mo et al. (2006)

#### Cicadidae

247933B9-9748-5F68-BCBF-B0734175E91F

#### Balinta

Distant, 1905

3B997177-7E6B-5BE0-BB1C-63E811B28D39

#### Balinta kershawi

Kirkaldy, 1909

5F61BE1D-44A8-5397-B3A9-E94AC45B80DD

##### Notes

Kirkaldy (1909)

#### Chremistica

Stål, 1870

13DADBAB-710C-5975-AC42-5A6FDA0A6868

#### Chremistica ochracea

(Walker, 1850)

20228DBE-416F-57C9-8455-18503BFFC021

##### Notes

DSPA (2022)

#### Cryptotympana

Stål, 1861

2F6AB58D-94CC-5885-AFDC-ACB2691361FB

#### Cryptotympana atrata

(Fabricius, 1775)

D6BEA82B-4FC4-5FCF-8EA4-790E9AD3799A

##### Notes

Pun and Batalha (1997)

#### Cryptotympana mandarina

Distant, 1891

3CA795F3-A565-5917-8E62-382D5AAD1653

##### Notes

Easton (1992)

#### Cryptotympana pustulata

(Fabricius, 1775)

67ED82C9-CDCC-595C-A3AE-9D30D76B51AD

##### Notes

Easton (1991)

#### Gaeana

Amyot & Audinet-Serville, 1843

621830B6-D904-5135-B514-E3D49F2BDC28

#### Gaeana maculate

(Drury, 1773)

2F2F07DB-14EB-518E-89EC-8AD46513C8A1

##### Notes

Pun and Batalha (1997)

#### Huechys

Amyot & Serville, 1843

E51BFA50-3389-5C50-AFE0-CAC068B45BE9

#### Huechys sanguinea

(Geer, 1773)

DDB1FD93-1489-55DB-8F68-2638C3190909

##### Notes

Pun and Batalha (1997)

#### Platypleura

Amyot & Audinet-Serville, 1843

980436E8-5701-57BD-9731-CE1A26184142

#### Platypleura hilpa

Walker, 1850

4E6EB571-68E3-596E-A148-6294E2B9A8AB

##### Notes

Pun and Batalha (1997)

#### Coccidae

D98F53A3-99FF-5D71-88FD-F08E8C309940

#### Ceroplastes

Gray, 1828

1DAA4AC3-EB88-52A1-964F-8DCFAE941698

#### Ceroplastes ceriferus

(Fabricius, 1798)

A46C967D-8729-5592-92D6-52D8D7B7E01D

##### Notes

Pun and Batalha (1997)

#### Saissetia

Déplanche, 1859

1D344119-2552-5597-90CA-83596E7A348F

#### Saissetia hemisphaerica

(Targioni-Tozzetti, 1867)

B049F0BB-14DD-5A00-A418-EEC68456665C

##### Notes

Pun and Batalha (1997)

#### Coreidae

C8C1C65C-0F34-5975-96C7-0EA1E4AD927C

#### Acanthocoris

Amyot & Serville, 1843

2141C505-63FD-51E8-BC28-B0E98C183542

#### Acanthocoris scaber

(Linnaeus, 1763)

581E7ED8-F82A-5EB9-80CE-5261C2F687A2

##### Notes

Pun and Batalha (1997)

#### Cletus

Stål, 1860

062C27AB-2A01-59E8-94C6-C622C5676B56

#### Cletus punctiger

(Dallas, 1852)

96EB3430-E874-591D-A2C5-21F893A6BC06

##### Notes

DSPA (2022)

#### Cletus trigonus

(Thunberg, 1783)

27065B3F-4F19-5A97-B934-1E42054D0092

##### Notes

Pun and Batalha (1997)

#### Gralliclava

Dolling, 1978

4348A444-F0E2-5636-8047-DAE0C6434E85

#### Gralliclava horrens

(Dohrn, 1860)

2260BBB1-3A31-5047-AF6A-1A526872349D

##### Notes

Pun and Batalha (1997)

#### Homoeocerus

Burmeister, 1835

72A5E5A3-444F-53D4-A60E-F51A2BFB0528

#### Homoeocerus unipunctatus

(Thunberg, 1783)

48006D7B-1D28-5A7F-A744-37F0AC534C75

##### Notes

Pun and Batalha (1997)

#### Mictis

Leach, 1814

1568F8AA-C2B3-58CD-BB0D-98B8F92CC0FA

#### Mictis tenebrosa

(Fabricius, 1787)

B2C3FC5E-5C54-52AB-9A91-65FF8A5CC049

##### Notes

Pun and Batalha (1997)

#### Notobitus

Stål, 1860

3269BB2C-1898-5C5A-A62C-E0746F69B1D6

#### Notobitus meleagris

(Fabricius, 1787)

CABAB619-F5E2-5B24-92E4-A758D2143660

##### Notes

Pun and Batalha (1997)

#### Paradasynus

China, 1934

09B1F5C9-F2B5-51B3-A4F3-295213CA2810

#### Paradasynus spinosus

Hsiao, 1963

A5BDA5B7-F016-534C-9ECA-40385BD5D02C

##### Notes

Pun and Batalha (1997)

#### Cydnidae

DCD2415D-72A4-5C03-B535-5FF4A041AAE4

#### Adrisa

Amyot & Serville, 1843

4D142E6E-044F-5928-84DE-1A08EFFB8EB1

#### Adrisa magna

(Uhler, 1860)

FA9C2DC3-C050-5DF3-B7A6-C30E0D804243

##### Notes

Pun and Batalha (1997)

#### Fromundus

Distant, 1901

B8F9BD56-D4DA-5031-B8E0-B0E82FDDE812

#### Fromundus pygmaeus

(Dallas, 1851)

E369DFA1-C870-5F05-8863-D472EF64A4FE

##### Notes

DSPA (2022)

#### Delphacidae

A568C0EA-5B21-5909-8002-DD6F6F32B7CB

#### Belocera

Hyatt, 1884

BE56D3CF-28D3-5AD6-BF10-F6A0F032E8B8

#### Belocera sinensis

Muir, 1913

FE1CDE40-D9B4-55F0-AA2D-C30CF31E5C0D

##### Notes

Brassard et al. (2020)

#### Cemus

Fennah, 1964

809122F6-4344-5EFF-8BB6-25FD4F1CC1FA

#### Cemus macaoensis

(Muir, 1913)

90A6BD71-4585-5ECB-82E8-AE7695F4F3D9

##### Notes

Brassard et al. (2020)

#### Opiconsiva

Distant, 1917

15DB33D1-1F4D-56B6-86F4-3A66EE9FD224

#### Opiconsiva sameshimai

(Matsumura & Ishihara, 1945)

8AA36101-D659-5CF3-95B2-1EF9FA484B19

##### Notes

DSPA (2022)

#### Pseudaraeopus

Kirkaldy, 1904

82F57D4D-E20B-558D-B56F-6FCE0DD3B6C5

#### Pseudaraeopus sacchari

(Muir, 1913)

36B7E05A-A23B-558B-BD2B-3D653ED62830

##### Notes

Brassard et al. (2020)

#### Diaspididae

BEBC4A8D-9744-5775-BA1F-6B3516229FED

#### Aulacaspis

Cockerell 1893

8DC3A3C2-FD7B-59DB-B8BC-8E93B69AE871

#### Aulacaspis thoracia

(Robinson, 1917)

CB082A0C-AC2F-58F1-BCF2-BB97CB1AE4E2

##### Notes

Mo et al. (2006)

#### Aulacaspis rosarum

Borchsenius, 1958

25455881-B841-5648-8B0E-3C5FBF7DA286

##### Notes

Pun and Batalha (1997)

#### Aulacaspis yabunikkei

(Kuwana, 1926)

CD66E928-752F-583D-BC17-0DFB2C64D8E8

##### Notes

Pun and Batalha (1997)

#### Hemiberlesia

Cockerell, 1897

CB62B4A6-086D-55C2-BF2E-06FF8B2C548A

#### Hemiberlesia pitysophila

Takagi, 1969

82C3DED4-D4A5-5F1B-8671-723BF7C32FD5

##### Notes

Pun and Batalha (1997)

#### Lepidosaphes

Shimer, 1868

D2604EA3-EF87-542B-B4C7-AF5AD62C6532

#### Lepidosaphes laterochitinosa

Green, 1925

BD94914C-EBEB-514B-8198-A5E24BC9DA77

##### Notes

Pun and Batalha (1997)

#### Parlatoria

Targioni-Tozzetti, 1868

F60BB6E6-1582-5BBA-A4AD-A4B313CD5EB2

#### Parlatoria pergandii

Comstock, 1881

525EDEF5-9BA6-50E1-9F3E-92347B9888F5

##### Notes

Pun and Batalha (1997)

#### Parlatoria proteus

(Curtis, 1843)

10BE9E5D-AADF-5002-AEF9-3842B3C19D2E

##### Notes

Mo et al. (2006)

#### Pseudaulacaspis

Mac Gillivray, 1921

9155A916-C682-53C2-98C5-B9F6A3DEA3D6

#### Pseudaulacaspis cockerelli

(Cooley, 1897)

5F673940-7A14-5253-9191-EFD574D571A5

##### Notes

Pun and Batalha (1997)

#### Pseudaulacaspis pentagona

(Targioni-Tozzetti, 1886)

335A2986-A48F-56FE-8485-A1C7F044FBBA

##### Notes

Mo et al. (2006)

#### Dictyopharidae

08BF30BA-D9FA-5791-8300-8C2EC6F91859

#### Dictyophara

Germar, 1833

172A6443-2A52-571F-8A28-C25B00569E29

#### Dictyophara patruelis

(Stål, 1859)

C8C8530C-384C-5C99-8C58-5B509B1D1291

##### Notes

Mo et al. (2006)

#### Dinidoridae

CAB2EBAE-9D6A-54F2-96EF-BF85D4F9C5EF

#### Cyclopelta

Amyot & Serville, 1843

BE606E16-7AF9-505A-B9F8-66D230B33D74

#### Cyclopelta obscura

(Lepeletier & Serville, 1828)

0CE80F25-A575-503A-99FC-8861BC790EAB

##### Notes

Pun and Batalha (1997)

#### Megymenum

Guérin-Méneville, 1831

9BB3719B-F45E-5E69-87BC-F38144B05B69

#### Megymenum brevicorne

(Fabricius, 1787)

453AE164-780E-5376-BAF9-A48EC623E6EF

##### Notes

Pun and Batalha (1997)

#### Megymenum inerme

(Herrich-Schäffer, 1840)

C413DCDD-6AAE-513F-BA38-BA63153D6B04

##### Notes

Easton and Pun (1997a)

#### Flatidae

2A711FC2-B462-5CB3-ACDC-15FB4ADE1BFC

#### Lawana

Distant, 1906

F697358E-BCF7-562C-92C1-94EC6B632D32

#### Lawana imitata

(Melichar, 1902)

90514166-7698-56A5-898E-0F7999B5D10F

##### Notes

Pun and Batalha (1997)

#### Salurnis

Stål, 1870

34B608E8-422B-5B7C-BF3E-0D3666E63723

#### Salurnis marginella

(Guérin-Méneville, 1829)

8A3E2BCD-8170-5725-A10B-1E89E036C8E7

##### Notes

DSPA (2022)

#### Fulgoridae

1433D314-F64F-552A-AAAF-C7566C82B6A7

#### Fulgora

Linnaeus, 1767

14D83A02-E50F-5536-9D02-510940B2F32A

#### Fulgora candelaria

(Linnaeus, 1758)

40ADD77D-2BB3-5547-B91F-A4E26DA2CE1D

##### Notes

Pun and Batalha (1997)

#### Gerridae

541C8A3B-23CE-5F9D-A6B1-A1554091EC4A

#### Aquarius

Schellenberg, 1800

9E0455C2-0402-5C27-951D-3A4DEE180149

#### Aquarius paludum

(Fabricius, 1794)

86CE143F-13B3-5134-8930-4408BAC3D6DA

##### Notes

Easton and Pun (1997a)

#### Largidae

3272E24B-1C52-589B-88F1-0461F67B2067

#### Physopelta

Amyot & Serville, 1843

19A1D78A-9FBA-5666-940A-444E449CDAA1

#### Physopelta gutta

(Burmeister, 1834)

7F608EC6-BF10-5623-8635-0A65D7E2A7B3

##### Notes

Pun and Batalha (1997)

#### Lygaeidae

2833E1BE-CC51-50AE-8BA9-778E3D9FF9B7

#### Geocoris

Fallen, 1814

1ED2F5C0-8181-5878-9AA2-185DA51AD6E6

#### Geocoris ochropterus

(Fieber, 1844)

B7CBCF3A-B194-54FF-A2E2-05BB34CDF7B1

##### Notes

DSPA (2022)

#### Graptostethus

Stål, 1868

EDAD17FC-77DE-5FD3-AAA9-D3D63E08B350

#### Graptostethus servus

Fabricius, 1787

A0E7EC40-509F-5097-92A9-067CA7D83E85

##### Notes

DSPA (2022)

#### Nysius

Dallas, 1852

D4CCBB0F-C3A1-55D0-9875-5B32966E4E51

#### Nysius inconspicus

Distant, 1904

E2951095-AEA6-561E-8CE8-9A1FD0DFC198

##### Notes

DSPA (2022)

#### Margarodidae

30BCA258-DD68-57B5-BB8D-FD1B194DE4FC

#### Lcerya

Signoret, 1875

9A2707BA-A3D9-5AEA-BEBC-7FBC4A541779

#### Lcerya aegyptiaca

(Douglas, 1890)

5EA667DB-E273-5431-9EB7-2750345E1EB1

##### Notes

DSPA (2022)

#### Lcerya purchasi

(Maskell, 1878)

55D2B339-4E1A-5B8E-BE42-30B3A9FE3160

##### Notes

Pun and Batalha (1997)

#### Membracidae

0CE4C95E-02F8-5F16-A287-D70123EA5760

#### Tricentus

Stål, 1866

FF9C15BF-5636-5672-AF1C-0DBFA7DD224B

#### Tricentus albomaculatus

Distant, 1908

5A5F1AD7-C1F6-5190-8341-2E4555BCED35

##### Notes

DSPA (2022)

#### Miridae

315E0625-ABC5-5D1D-B6D4-167A0474759D

#### Lygocoris

Reuter, 1875

16370E82-258C-56DC-8A39-E43971BFAAA3

#### Lygocoris lucorum

Hoberlandt, 1956

78FB8B52-9868-5193-B1BB-FC70275AF2DA

##### Notes

DSPA (2022)

#### Stenodema

Laporte, 1832

04DD0856-3D89-5C42-86D6-B90650C14080

#### Stenodema elegans

Reuter, 1904

2221E302-DB2A-5BA4-83B9-DE58F3B2885C

##### Notes

DSPA (2022)

#### Nabidae

D94C5E18-D9CF-5A82-BFAA-8AD9ADF5DB50

#### Nabis

Latreille, 1802

8312908B-AD9C-5CB1-A25E-66AA0F565A39

#### Nabis stenoferus

Hsiao, 1964

EB8EE0CE-58AB-518A-A3FD-50AF6A157C38

##### Notes

Pun and Batalha (1997)

#### Notonectidae

17FE538E-B6B9-59EE-89BC-EFDF5A4FC347

#### Enithares

Spinola, 1837

F6A31641-FCBB-5429-8356-46395AA65FDA

#### Enithares biimpressa

(Uhler, 1860)

809C6DEE-6A94-50D6-8DF0-F60A5CF6612B

##### Notes

Easton and Pun (1997a)

#### Pentatomidae

38B71F06-8004-5E50-98D0-801918513971

#### Dalpada

Amyot & Serville, 1843

751E72E1-97CD-5833-9FA4-EC66B012AFB5

#### Dalpada nodifera

Walker, 1867

D657092D-0CF9-5AC3-9200-C5A1F4857D47

##### Notes

Mo et al. (2006)

#### Dalpada oculata

(Fabricius, 1775)

45261146-987F-54AE-A1C1-52DB2458DFDD

##### Notes

Pun and Batalha (1997)

#### Eocanthecona

Bergroth, 1915

9DACBAEC-024D-526F-A5CF-1FB3D0FF089F

#### Eocanthecona concinna

(Walker, 1867)

ECE5823F-F113-5E19-A7E9-2A7BD0BB1220

##### Notes

Pun and Batalha (1997)

#### Eocanthecona furcellata

(Wolff, 1801)

ADB13C79-32D5-5C39-BC5C-2E9708366A9C

##### Notes

Easton and Pun (1997a)

#### Erthesina

Spinola, 1837

0D1FD807-B11E-51A9-9EE5-209B4BB97390

#### Erthesina fullo

(Thunberg, 1783)

3890CA0B-AD3A-5B30-88FE-3980C8F6DB9C

##### Notes

Pun and Batalha (1997)

#### Eysarcoris

Hahn, 1834

AA296295-97B4-53DE-82EF-E03F5B1F4C49

#### Eysarcoris guttigerus

(Thunberg, 1783)

9602C613-A273-51E3-9BAA-F4475D37F004

##### Notes

Easton and Pun (1997a)

#### Halyomorpha

Mayr, 1864

CC367CB1-11D1-5F87-A964-E52B00F4B755

#### Halyomorpha halys

(Stål, 1855)

06B9E3A4-4819-583B-8BEA-0ABE4708B1BC

##### Notes

Easton and Pun (1997a)

#### Melanophara

Stål, 1867

F9F5132C-7991-5BD6-9001-79D4BDAD8842

#### Melanophara dentata

Haglund, 1868

E9CE9E59-A23E-5A33-8F0E-CF0FC6D1408F

##### Notes

Easton and Pun (1997a)

#### Megarrhamphus

Bergroth, 1891

4FD87EBE-CD0A-544A-8007-21026978E340

#### Megarrhamphus hastatus

(Fabricius, 1803)

C934EA8E-EF04-5CE8-8526-D9EB19FCDFF0

##### Notes

Easton and Pun (1997a)

#### Nezara

Amyot & Serville, 1843

E3909102-925D-5ED6-AD59-C8CF0388FB54

#### Nezara viridula

(Linnaeus, 1758)

B6CDDEEC-F8C0-59B1-B629-C5D7E84E0133

##### Notes

Pun and Batalha (1997)

#### Piezodorus

Fieber, 1860

19624A6C-5353-57D2-B8BB-72E6876BAF6F

#### Piezodorus hybneri

(Gmelin, 1790)

A82D5A9E-BAF2-51DC-9BE0-5BCBCF7001F0

##### Notes

Easton and Pun (1997a)

#### Plautia

Stål, 1865

33B7EAA3-CAE0-51BD-AAE5-37C75BFAB0C6

#### Plautia crossota

(Dallas, 1851)

3B2FD9C8-D882-54CB-88D0-202B31BE2AED

##### Notes

Easton and Pun (1997a)

#### Rhynchocoris

Westwood, 1837

64912A64-EC0E-5AFE-9C2D-319D1D499F7C

#### Rhynchocoris humeralis

(Thunberg, 1783)

F455CB6A-F51F-5793-BCA7-A23E9327E7F3

##### Notes

Pun and Batalha (1997)

#### Scotinophara

Stål, 1867

1066379F-FC47-5C13-BECF-CF3941C6565E

#### Scotinophara bispinosa

Fabricius, 1798

6C20B901-3EF5-559E-9FFE-EF7019626474

##### Notes

DSPA (2022)

#### Scotinophara scotti

Horváth, 1879

78EB0224-5122-5F96-BCAF-578D0EF0E441

##### Notes

DSPA (2022)

#### Tetroda

Amyot & Serville, 1843

BC896C5E-C4B5-53C3-95B4-485A8646A87D

#### Tetroda denticulifera

Bergr, 1915

664FA5CA-60BA-5F83-9E53-8A1A51FD7F56

##### Notes

Easton and Pun (1997a)

#### Tetroda histeroides

(Fabricius, 1798)

EC39615E-63C0-5DBC-8CEC-06A5540C5FC6

##### Notes

Pun and Batalha (1997)

#### Tolumnia

Stål, 1868

F5831A7E-F6BD-521F-9796-5FBA9589C920

#### Tolumnia latipes

(Dallas, 1851)

CBD336D3-4743-5911-9A74-00607219B54B

##### Notes

Pun and Batalha (1997)

#### Eysarcoris

Hahn, 1834

056A5E23-962B-52E7-9952-47EC14383FFF

#### Eysarcoris guttigerus

(Thunberg, 1783)

42716DF3-FAE1-59E3-9381-5F0D706F90A6

##### Notes

Pun and Batalha (1997)

#### Udonga

Distant, 1921

715F7A45-0F25-5D4F-9A1C-7C1E5843EC19

#### Udonga spinidens

Distant, 1921

4446E7AD-A8A5-5BE5-9D12-9EA617C1777D

##### Notes

Easton and Pun (1997a)

#### Zicrona

Amyot & Aerville, 1843

E797E185-0C64-513E-AF6B-6CA034ACEBF2

#### Zicrona caerulea

(Linnaeus, 1758)

E1CA73C2-C1C9-5E1C-9A29-A07FC26BEF7F

##### Notes

Easton and Pun (1997a)

#### Plataspidae

8A1C4EC0-CD1D-5C1D-A9F5-8E34F4B909D5

#### Brachyplatys

Boisduval, 1835

6ED26B5D-1F7D-5A7B-809D-88F6D35DD872

#### Brachyplatys subaeneus

(Westwood, 1837)

67D9BCAD-98F2-5643-B94B-259A771C7F79

##### Notes

Pun and Batalha (1997)

#### Coptosoma

Laporte, 1833

5C03B82F-2999-5046-889F-FE55CFF5D1B4

#### Coptosoma variegatum

(Herrich-Schaeffer, 1838)

F0E9D2A0-F105-5EE3-9644-E7895E6E2416

##### Notes

Pun and Batalha (1997)

#### Megacopta

Hsiao & Ren, 1977

20CFE8CD-84BA-5716-85F4-2F33052416DC

#### Megacopta cribraria

(Fabricius, 1798)

ED5DFEA4-007F-5FF2-AA66-4D581F687627

##### Notes

Pun and Batalha (1997)

#### Pseudococcidae

7708FF17-E5F2-5D39-AE14-41C432D7620D

#### Dysmicoccus

Ferris, 1950

92189C27-835D-5AC9-A7BF-845B9F163476

#### Dysmicoccus neobrevipes

(Beardsley, 1959)

D4789215-71B3-5319-956C-918A92383BD6

##### Notes

Fu et al. (2012)

#### Eumnyrmococcus

Silvestri, 1926

98A13A11-6DFB-5CE9-8AC3-75DF06F131D8

#### Eumnyrmococcus smithii

Silvestri, 1926

2482CE8E-E53B-5F1D-BAA6-5A02FE05BD83

##### Notes

Fang et al. (2001)

#### Maconellicoccus

Ezzat, 1958

B663BCA5-4FDC-59C8-BBE8-5D893CF7927E

#### Maconellicoccus hirsutus

(Green, 1908)

12FB52B9-E12B-5DDB-9F3C-4472A49828E9

##### Notes

Pun and Batalha (1997)

#### Psyllidae

BC861E12-B522-5D9A-AA65-F83833151265

#### Diaphorina

Low, 1879

19FF9026-B86E-55BF-A866-36724479D7FA

#### Diaphorina citri

Kuwayama, 1908

80FE14E9-C75C-5E8B-99FC-932E435BDA85

##### Notes

Kondo et al. (2015)

#### Macrohomotoma

Kuwayama, 1907

F2A34721-A046-5157-AAA7-FC499E62DF8F

#### Macrohomotoma striatum

Crawford, 1925

E1572F47-6AC5-5A0E-B8E5-5B733A5E4D8A

##### Notes

Pun and Batalha (1997)

#### Pyrrhocoridae

C9876F98-38DC-514C-A238-8B4E6461F317

#### Dindymus

Stål, 1861

3D2AC05F-6EB8-563F-BF68-EB9ED22D23E7

#### Dindymus sanguineus

(Fabricius, 1787)

35C7D43B-98B3-5185-A496-2EC3681D4673

##### Notes

MBD (2022)

#### Dindymus rubiginosus

(Fabricius, 1787)

B98BA070-6F25-5442-BF6E-D423E05A42C2

##### Notes

Easton and Pun (1997a)

#### Dysdercus

Amyot & Setville, 1831

B247A90B-3501-5F63-8116-3C090D39E281

#### Dysdercus cingulatus

(Fabricius, 1775)

7719D934-7C2C-51E1-83BC-1DF06F4DBD8F

##### Notes

Mo et al. (2006)

#### Dysdercus poecilus

(Herrich-Schaefer, 1843)

D38960EF-4FB5-52C7-AF61-374D8C8A0FF3

##### Notes

DSPA (2022)

#### Leptocoris

Hahn, 1833

BAC5E09C-0CCF-57DD-834F-8F5C0726625D

#### Leptocoris augur

(Fabricius, 1781)

53A83E2A-4CF5-58A2-AA41-DB3386E13BCE

##### Notes

DSPA (2022)

#### Reduviidae

5688FE14-BC29-53A2-B2FA-CE75B60B779A

#### Ectomocoris

Mayr, 1865

C6727BDF-F59D-50CB-90DA-406FBDA4C744

#### Ectomocoris apicimaculatus

Distant, 1919

3E7C6D4C-2B24-58A7-AB9C-8DED91DA5415

##### Notes

Easton and Pun (1997a)

#### Ectomocoris atrox

(Stål, 1855)

CD51B6EB-4C14-599D-86B0-1323FB96369A

##### Notes

Pun and Batalha (1997)

#### Ectrychotes

Burmeister, 1835

CBC8C76F-8AED-57F4-AD2A-57769D8C6B09

#### Ectrychotes andreae

(Thunberg, 1784)

DABAA508-855D-5342-A85A-AD40DCA6514F

##### Notes

Pun and Batalha (1997)

#### Oncocephalus

Klug, 1830

563A67D2-3C73-5BB2-BF0F-8C4E4581EEEA

#### Oncocephalus impudicus

Reuter, 1882

1B4D2B0C-D0A0-5877-9048-193E156A199B

##### Notes

Easton and Pun (1997a)

#### Opistolatys

Westwood, 1834

E6A3F917-ECB3-529F-840D-2D574DF2FFAE

#### Opistolatys perakensis

Miller, 1940

CB3ADA9E-8681-511C-9DDE-35CAB74E9879

##### Notes

DSPA (2022)

#### Polididus

Stål, 1858

D909CB39-6842-5152-86C1-3853482E3A61

#### Polididus armatissimus

Stål, 1860

A5C49BA6-6B9B-5DFF-B540-824BB5F976E4

##### Notes

Easton and Pun (1997a)

#### Sastrapada

Amyot & Serville, 1843

F4B16656-3B79-5689-B590-3FAC56E9ECFF

#### Sastrapada hsiaoi

Maldonado, 1987

7203D3D7-C0C8-5441-99D9-738C29F4965F

##### Notes

DSPA (2022)

#### Scadra

Stål, 1859

98E91567-8090-5F78-B097-0801CF754890

#### Scadra costalis

(Lethierry, 1888)

83822282-7D24-50A9-BC10-0781E8506E79

##### Notes

Easton and Pun (1997a)

#### Scadra wuchengfui

China, 1940

B9FDBE0D-4708-54F4-BB35-BEFDB7FA8641

##### Notes

DSPA (2022)

#### Sycanus

Amyot & Serville, 1843

BBEDDA0F-2B88-5390-A980-4A5C763D2C15

#### Sycanus crocevittatus

Dohrn, 1859

0A972F9A-4C46-5726-8A3B-8E064111A2A5

##### Notes

Easton and Pun (1997a)

#### Triatoma

Laporte, 1833

E7F35D5D-BC55-530C-9E19-8A25EF872646

#### Triatoma rubrofasciata

(DeGeer, 1773)

493AFE39-D9AB-561F-A51A-FDA63E8FAAA4

##### Notes

Easton and Pun (1997a)

#### Tribelocephala

Stål, 1854

C79CA010-D9E5-5060-8706-BDB92C57ACF3

#### Tribelocephala walkeri

China, 1940

5ED77BDF-D843-5E50-BD34-270DE1397B38

##### Notes

Pun and Batalha (1997)

#### Rhyparochromidae

BBB1D632-63CF-5769-BA04-BADA553693B5

#### Horridipamera

Malipatil, 1978

A109E7C7-03E1-5C17-9BF7-0DF92AD6C338

#### Horridipamera nietneri

(Dohrn, 1860)

01DCCB5A-7DB3-5B7D-9087-EDF0B4948DE4

##### Notes

Easton and Pun (1997a)

#### Metochus

Scott, 1874

22FD6869-0EBE-59E0-BA08-732D8C7AB43B

#### Metochus abbreviatus

Scott, 1874

6BF2F84D-2D5E-51C7-B9C8-7E7A15D96160

##### Notes

Pun and Batalha (1997)

#### Metochus hainanensis

Zheng & Zou, 1981

118A2668-C125-5062-BF43-E901F815E521

##### Notes

Easton and Pun (1997a)

#### Metochus uniguttatus

(Thunberg, 1822)

A1285770-CF4C-5A5F-8F41-95EE6E36C998

##### Notes

Easton and Pun (1997a)

#### Pamerana

Distant, 1909

D0CAC31A-565D-59BD-9DF0-C3AB82F314F2

#### Pamerana scotti

(Distant, 1901)

8797422C-2B62-5EB3-ACA5-377AECEC2B3B

##### Notes

DSPA (2022)

#### Paromius

Fieber, 1860

0A624A72-EB24-58E9-A018-1AAE812FA766

#### Paromius exiguus

(Distant, 1883)

3409ECEF-F715-5F7D-9B6F-2FCD7A6ECA16

##### Notes

Easton and Pun (1997a)

#### Paromius piratodes

(Costa, 1864)

B38AAFDA-9996-5269-9277-9999A1023020

##### Notes

DSPA (2022)

#### Ricaniidae

F1C14A17-9E02-5500-8867-D6E39B68CE53

#### Ricania

Germar, 1818

D543ADE7-1944-5699-879C-3B8E6D546449

#### Ricania guttata

(Walker, 1851)

FA8EF363-BFAD-5676-8F0E-0FD53F75B84D

##### Notes

MBD (2022)

#### Scutelleridae

6599C0F3-B653-59B1-967F-5EB237359BED

#### Calliphara

Germar, 1839

DC820978-A83C-5136-AC35-E58EA12B63BA

#### Calliphara nobilus

(Linnaeus, 1763)

14132E85-A5C7-5F1E-8712-49F85A51E468

##### Notes

Easton and Pun (1997a)

#### Cantao

Amyot & Serville, 1843

89F00721-DC3A-593D-AD9F-2A12D5C34C1E

#### Cantao ocellatus

(Thunberg, 1784)

747CFFFC-D305-5EEF-86E1-36EE22C89EAB

##### Notes

Pun and Batalha (1997)

#### Chrysocoris

Hahn, 1834

1E1288BC-18A1-5C46-8B84-DE6078A8166B

#### Chrysocoris fascialis

(White, 1842)

882F2EC3-DE17-5C47-8830-0B87225F648E

##### Notes

Tsai and Redei (2009)

#### Chrysocoris stollii

(Wolff, 1801)

187EBE70-1C2C-5F3C-BAAB-E98412CDF42D

##### Notes

Pun and Batalha (1997)

#### Tessaratomidae

70DB3C01-C899-5695-8EBB-60EEF78F53D3

#### Tessaratoma

Berthold, 1827

3F89FFDC-7EE9-5D9A-AF45-2342ED405E3A

#### Tessaratoma papillosa

(Drury, 1773)

C606295F-64AA-5AE9-B0A7-AE20FCBA3DE9

##### Notes

Pun and Batalha (1997)

#### Tingidae

1C5B368F-A1D4-5FF1-9587-1DA286F991DB

#### Stephanitis

Stål, 1873

ECA5A7D1-39B3-586C-9ED0-CAAFB116829A

#### Stephanitis pyrioides

(Scott, 1874)

1AF9253D-B3CB-57E4-8D16-B1E319DE9F6D

##### Notes

Pun and Batalha (1997)

#### Tropiduchidae

61B8B0E4-371C-5109-AB5C-7FE8A5AAE742

#### Kallitaxila

Kirkaldy, 1901

84640334-7D15-59F7-BBC7-5344AB19B2AF

#### Kallitaxila macaoana

(Muir, 1913)

5172C9AE-EC40-5A05-A8C7-05E2CA8A17FF

##### Notes

Brassard et al. (2020)

#### Trypetimorpha

Costa, 1862

887D9D5E-9FF6-53C7-B1CC-A1F6C9B650ED

#### Trypetimorpha biermani

(Dammerman, 1910)

0DC8DCC0-E818-5C2E-AAFC-4971A3EF401D

##### Notes

Brassard et al. (2020)

#### Diptera

31BA8F6C-32B7-506D-92C7-61E30A9CFDFD

#### Agromyzidae

1D6E29B9-599D-5A01-84AF-41C6439FFEFF

#### Liriomyza

Mik, 1894

5D22FF05-4DE6-5F11-8A6A-75966097C062

#### Liriomyza sativae

Blanchard, 1938

783CA351-57A3-596D-A7A9-ED44F25E1E0E

##### Notes

Mo et al. (2006)

#### Anthomyiidae

7455810A-2C4B-56BC-AA0F-41924F99D42B

#### Anthomyia

Meigen, 1803

D1627F1D-9F0D-54A9-BD9D-ABBBF867A681

#### Anthomyia illocata

Walker, 1856

D70FDD9B-0763-5B39-A7F1-7BABD0142852

##### Notes

DSPA (2022)

#### Bombyliidae

5B9C8FC1-56C5-5F7A-82C4-66A235BDDC7E

#### Ligyra

Newman, 1841

B40006BB-0B15-5735-8B6C-D6010FD09EB4

#### Ligyra tantalus

(Fabricius, 1794)

D55E8BB2-3D44-5CE9-A3EA-3C541E83EFD0

##### Notes

Pun and Batalha (1997)

#### Pterobates

Bezzi, 1921

CD8D1CB3-5A3C-545A-BA1A-AAE1B9AFEAC1

#### Pterobates pennipes

(Wiedemann, 1821)

7134B41B-4BDA-5EC3-8241-A9B52ADE9B7F

##### Notes

Yang et al. (2018)

#### Calliphoridae

6BD60D80-B845-5F4E-B897-715F16474D51

#### Chrysomya

Robineau-Desvoidy, 1830

B853154C-0DBB-5ED2-9FBB-D6176619B882

#### Chrysomya megacephala

(Fabricius, 1794)

CCCD61B1-1D0D-5281-8A25-CC04B19E0326

##### Notes

Easton (1992)

#### Hemipyrellia

Townsend, 1918

E3409533-EE7E-59BF-A900-AA7CC3A8E601

#### Hemipyrellia ligurriens

(Wiedemann, 1830)

D10131F9-2962-56D3-916C-D7C0377DB492

##### Notes

DSPA (2022)

#### Stomorhina

Rondani, 1861

82E31917-B840-52D8-8418-45B0F8BFB348

#### Stomorhina obsoleta

Wiedemann, 1830

A888AFED-CA31-5A40-AFA4-2CF2C14BAD08

##### Notes

DSPA (2022)

#### Tainanina

Villeneuve, 1926

9BCC423C-DB84-5A74-AF9E-DA948EE925E7

#### Tainanina pilisquama

(Senior-White, 1925)

F5E4379F-4DB7-51C1-B0EC-616CC31EEDC9

##### Notes

Kurahashi and Chowanadisai (2001)

#### Ceratopogonidae

4F11AEB3-1BF1-5188-9D16-6B2F5A94DAB3

#### Dasyhelea

Kieffer, 1911

AB2B0338-7C49-52D4-A33A-DB28A73F6A28

#### Dasyhelea dixa

Yu, Wu & Gong, 2010

B6CE2DDC-EC59-5C52-8275-4EC7E21C0A95

##### Notes

Sun et al. (2010)

#### Dasyhelea fornicatus

Yu & Liu, 2005

A25F33CA-8012-5EB3-94D5-BD6C4FDE78FE

##### Notes

Yu and Huang (2006)

#### Dasyhelea gongylophoda

Yu, 2006

4963FBBA-A5A2-5284-AE64-306E3F8ED03E

##### Notes

Yu and Huang (2006)

#### Dasyhelea hei

Yu, Sun & Gong, 2010

60DC9D29-29BD-5FBC-B2AF-176337666139

##### Notes

Sun et al. (2010)

#### Dasyhelea ludingensis

Zhang & Yu, 1996

59A6D70B-D5B6-5D6A-8B29-2A4A18A357D3

##### Notes

Yu and Huang (2006)

#### Dasyhelea linlinigae

Yu, 2006

6C208D9C-26BA-5D24-B270-7A3E19E9DA3E

##### Notes

Yu and Huang (2006)

#### Dasyhelea occasus

Zhang & Yu, 1996

E13D97BA-1977-5A41-96C9-713C1937A177

##### Notes

Sun et al. (2010)

#### Forcipomyia

Meigen, 1818

23A0324E-7C50-5945-A1F0-4B6FAF2EFE15

#### Forcipomyia hydatus

Liu, Yan & Liu, 1996

3D184F32-02AE-5190-AB3F-58427A985512

##### Notes

Yu and Huang (2006)

#### Forcipomyia psilonota

(Kieffer, 1911)

A78CB7AF-8B71-5454-8CE0-58F110AB3ACB

##### Notes

Yu and Huang (2006)

#### Lasiohelea

Keiffer, 1921

18F9227E-15BC-598A-A89A-9F1D3D161D77

#### Lasiohelea taiwana

Shiraki, 1923

F7BBC82C-D34B-5A74-9D76-786CAA090545

##### Notes

Yu and Huang (2006)

#### Chironomidae

46F6D228-E540-5F9B-8F72-D62519F57357

#### Ablabesmyia

Johannsen, 1905

4339246B-17EF-5A9F-8538-0761F1B85A71

#### Ablabesmyia alba

Chaudhuri, Debnath & Nandi, 1983

B2BE9EB2-2493-5E1E-822A-5CDF25080BF1

##### Notes

Niitsuma and Tang (2019)

#### Ablabesmyia maculitibialis

Chaudhuri, Debnath & Nandi, 1983

EBEB12FD-F975-57A7-8283-7E7302D34827

##### Notes

Niitsuma and Tang (2019)

#### Ablabesmyia pectinata

Niitsuma & Tang, 2019

40673468-1598-514A-8EC5-E8A6CBFF6A6B

##### Notes

Niitsuma and Tang (2019)

#### Nilodosis

Kieffer, 1921

611B3CF5-63DF-5FC2-B6BD-E407C6C0A32C

#### Nilodosis austrosinensis

Tang & Cranston, 2017

8FF10431-A65D-5585-BEA7-C5BDAF75B7B7

##### Notes

Tang and Cranston (2017)

#### Paratanytarsus

Thienemann & Bause, 1913

66ECAB65-719D-5B1E-9F76-FAC269BA1C0B

#### Paratanytarsus nanyuensis

Li & Tang, 2021

C23A2CDD-5E98-5229-8389-D72EC7B81884

##### Notes

Li and Tang (2021)

#### Polypedilum

Kieffer, 1921

52B49538-F587-572B-947E-60363D723BA6

#### Polypedilum masudai

(Tokunaga, 1938)

548783DA-0EE5-5BE6-945A-3243D9969BC2

##### Notes

Tang et al. (2014)

#### Polypedilum nodosum

(Johannsen, 1932)

226E9487-EEC0-5FEC-B0FA-6E30BA938775

##### Notes

Tang et al. (2014)

#### Culicidae

1EA6C3B4-3EA8-5B98-B2AD-69030783695F

#### Aedes

Meigen, 1818

AC74D5D3-B3D8-5F60-A5E3-0ECE4FD639F6

#### Aedes aegypti

(Linnaeus, 1762)

1E4BA062-A235-5B90-9B63-476F02EAC8BA

##### Notes

Qu (2001)

#### Aedes albopictus

(Skuse, 1895)

3001BC8C-91EB-59C4-A674-15EB465D56AD

##### Notes

Qu (2001)

#### Aedes togoi

(Theobald, 1907)

15A4DBB7-D7BF-5A45-82B3-7888A167D467

##### Notes

Qu (2001)

#### Aedes taeniorhynchus

(Wiedemann, 1821)

F510CFB1-A5DD-5E78-9F54-75DD34DFAE00

##### Notes

Lázaro et al. (2017)

#### Aedes w-albus

(Theobald, 1905)

19DF6B0F-8166-5C76-BB4F-D43A23089753

##### Notes

Ribeiro (1997)

#### Anopheles

Meigen, 1818

3D59FD32-2D69-5D81-AE42-00F5500CC61F

#### Anopheles jeyporiensis

James, 1902

E7222054-A7FF-5E35-A2BA-AB84F5439943

##### Notes

Qu (2001)

#### Anopheles karwari

(James, 1903)

F9CE54D6-726A-5192-8352-22D09E668CD8

##### Notes

Qu (2001)

#### Anopheles maculatus

Theobald, 1901

90AE8567-87AC-5ABC-BA61-7E927BED957E

##### Notes

Qu (2001)

#### Anopheles minimus

Theobald, 1901

B2E5A21D-972A-59F3-9AAB-045DC04C27DA

##### Notes

Qu (2001)

#### Anopheles sinensis

Wiedemann, 1828

96E68647-760E-5EB7-9203-9E3E0FF0B5E1

##### Notes

Qu (2001)

#### Anopheles tessellatus

Theobald, 1901

6CF3D94C-97D0-55AE-92D2-AE72A32B9E83

##### Notes

Qu (2001)

#### Armigeres

Theobald, 1901

C1924BA7-79D7-5281-B1A4-7378A296B406

#### Armigeres magnus

(Theobald, 1908)

3F78369B-D992-5582-A5B3-9F884D1F04EF

##### Notes

Qu (2001)

#### Armigeres subalbatus

(Coquillett, 1898)

EDE7E897-9282-562A-9807-C2BE24542F70

##### Notes

Qu (2001)

#### Culex

Linnaeus, 1758

9A591874-94BD-53CA-BFA4-BC4319540F8C

#### Culex bitaeniorhynchus

Giles, 1901

4283BEF2-240F-51E6-815D-0D67697B5A76

##### Notes

Qu (2001)

#### Culex fuscocephala

Theobald, 1907

6D2322F0-E7F2-5819-AFC1-843730A3E6EC

##### Notes

Qu (2001)

#### Culex jacksoni

Edwards, 1934

62C1A670-E8BF-5DF9-B86D-623EF89CA6E7

##### Notes

Qu (2001)

#### Culex pseudovishnui

Colless, 1957

A8F35C63-46F2-5648-A75A-92E359A3BF9F

##### Notes

Qu (2001)

#### Culex quinquefasciatus

Say, 1823

14AEF216-DAA2-57F4-A043-31FEAC0FCBBE

##### Notes

Qu (2001)

#### Culex sitiens

Wiedemann, 1828

22879905-3315-5A29-A813-80301673F608

##### Notes

Qu (2001)

#### Culex tritaeniorhynchus

Giles, 1901

6FCBD544-58E6-5A01-B3F8-198543415C21

##### Notes

Qu (2001)

#### Culex vagans

Wiedemann, 1828

C81E4C56-0D77-55BE-B476-39B22510505D

##### Notes

Qu (2001)

#### Culex vishnui

Theobald, 1901

7046EB15-E6FC-5AEB-8189-AF6B648C2EB6

##### Notes

Qu (2001)

#### Culex pallidothorax

Theobald, 1905

EA98BE70-B248-566A-8441-4BA3F7CB5CB8

##### Notes

Qu (2001)

#### Culex foliatus

Brug, 1932

2612B9AA-C64E-5C0D-8DDA-8899FE72B979

##### Notes

Qu (2001)

#### Culex malayi

(Leicester, 1908)

0644AC1B-DDFB-598D-8EED-EBF3DA5BDD5D

##### Notes

Qu (2001)

#### Culex infantulus

Edwards, 1922

DD6679EB-C799-5BC5-BA50-3DAD031ED746

##### Notes

Qu (2001)

#### Culex rubithoracis

(Leicester, 1908)

EF649C71-A826-5CC6-B230-65F470BE560D

##### Notes

Qu (2001)

#### Culex sumatranus

Brug, 1931

C25812F3-469E-5D2C-A0DE-6E0182CA39C4

##### Notes

Qu (2001)

#### Culex fascanus

Wiedemann, 1820

4E315B5F-6219-58AF-BEDB-452A07DABF6D

##### Notes

Qu (2001)

#### Lutzia

Theobald, 1903

3B30F512-A84C-507D-9356-44061A27B019

#### Lutzia halifaxii

(Theobald, 1903)

048C6E51-1282-5DB6-A798-B21841072C32

##### Notes

Qu (2001)

#### Mansonia

Blanchard, 1901

A98F842F-376D-5940-B912-36E4DE4D77E3

#### Mansonia uniformis

(Theobald, 1901)

598B611E-99B1-5ABF-AAFC-81093DC7A2B0

##### Notes

Qu (2001)

#### Mimomyia

Theobald, 1903

ABB96B96-1D6F-59BD-9589-B95DE13B4CC1

#### Mimomyia chamberlaini

Ludlow, 1904

0CD374B5-CC82-5080-9EB1-47348E7319A1

##### Notes

Qu (2001)

#### Toxorhynchites

Theobald, 1901

422C7855-2EBF-5C06-8704-1363C58D4752

#### Toxorhynchites macaensis

Ribeiro, 1997

C3B4F194-1BB1-51FE-9E27-A4B03B227445

##### Notes

Qu (2001)

#### Tripteroides

Giles, 1904

7AF8DF34-8027-5F22-8446-7B054E30AC30

#### Tripteroides aranoides

(Theobald, 1901)

61F92091-287F-5327-AF67-1BF52F5086C7

##### Notes

Qu (2001)

#### Uranotaenia

Lynch Arribálzaga, 1891

BCEED54D-7B60-55E2-BEAA-63B55BA09807

#### Uranotaenia annandalei

Berraud, 1926

E87D054F-26B7-519A-B1DA-5BE8E7691E58

##### Notes

Qu (2001)

#### Uranotaenia macfarlanei

Edwards, 1914

5656694F-4BED-5CC0-9195-D412FBF11B06

##### Notes

Qu (2001)

#### Dolichopodidae

7FB6F40C-E36D-56F0-9424-8C79F1934BCF

#### Chrysosoma

Guérin-Menéville, 1831

FEC8F45A-3DB6-5DA5-81FC-38FF9614B56E

#### Chrysosoma globiferum

(Wiedemann, 1830)

8B8424E6-3C5A-56FD-8D6F-8E3F6D447FB3

##### Notes

DSPA (2022)

#### Muscidae

868BC466-B745-5046-8344-0CA1347C5C89

#### Lispe

Latreille, 1796

F95EE8D9-DBF0-506D-9ECF-7D398E0B203D

#### Lispe assimilis

Wiedemann, 1824

B6724EBD-9395-5844-9AC4-4279F4D73E4D

##### Notes

DSPA (2022)

#### Musca

Linnaeus, 1758

38808607-D087-5354-BAB4-D0865BCBF37A

#### Musca domestica

Linnaeus, 1758

9FC33919-E5B4-5779-80BF-3FFBD329ACBC

##### Notes

DSPA (2022)

#### Musca sorbens

Wiedemann, 1830

6A3F84E1-DAC7-5D63-B6CA-A46957ABC429

##### Notes

DSPA (2022)

#### Muscina

Robineau-Desvoidy, 1830

9DCF8D94-E87A-5309-B9AE-BD679A1C5943

#### Muscina stabulans

(Fallén, 1817)

FDF9C378-C2A2-55FA-A798-9FE97F826A3C

##### Notes

DSPA (2022)

#### Ophyra

Robineau-Desvoidy, 1830

B31BD5BC-E9D3-5B77-ACE1-D35881CD8468

#### Ophyra chalcogaster

(Wiedemann, 1824)

3642735C-34FF-56AA-AE5A-28B6BAC72654

##### Notes

DSPA (2022)

#### Pygophora

Schiner, 1868

4E0AE33F-256B-5C56-B0E7-8F08186EC98F

#### Pygophora immaculipennis

Frey, 1917

4C435C6F-690B-5966-B4D9-6E5F86699B78

##### Notes

DSPA (2022)

#### Synthesiomyia

Brauer & Bergenstamm, 1893

E22F2F20-2BF7-5CFD-AC4F-7A3C107C3ACC

#### Synthesiomyia nudiseta

Wulp, 1883

9F09F917-3BA8-5BCF-B567-6D68030CB1FD

##### Notes

DSPA (2022)

#### Psychodidae

B86CCE9A-2D79-56C3-814E-A4A35C6EA4B6

#### Sergentomyia

Franca & Parrot, 1920

A75467A3-41E2-54C5-BCB9-932F5BB57BAD

#### Sergentomyia barraudi

(Sinton, 1929)

B6C9AB11-C141-5603-81B6-079773F960BA

##### Notes

Qu (2001)

#### Sarcophagidae

A430D966-5AFA-5131-ABC4-9B3358C14847

#### Boettcherisca

Rohdendorf, 1937

AAF063F2-414B-58B3-B5E6-FDF0F1799029

#### Boettcherisca peregrine

Robineau-Desvoidy, 1830

0E7916F8-09F2-5287-AB27-0654B4D7C0F4

##### Notes

DSPA (2022)

#### Sarcophaga

Meigen, 1826

27B45C92-2714-57C9-81FC-64F4633C5764

#### Sarcophaga kempi

(Senior-White, 1924)

FEC8E97E-1C74-5048-9230-38DDB81B9F94

##### Notes

DSPA (2022)

#### Sarcophaga albiceps

Meigen, 1826

7CD6BCBB-D417-5A89-B306-D13E1BC815E9

##### Notes

DSPA (2022)

#### Sarcophaga princeps

(Wiedemann, 1830)

F7F9BCD0-515F-5ABC-9665-394C8F47C45C

##### Notes

DSPA (2022)

#### Simuliidae

0D495EF0-ED6B-527E-9257-3DDA7287D6DE

#### Simulium

Latreille, 1802

9FCF472A-1DA1-56CD-B98D-6284F969BC27

#### Simulium aureohirtum

Brunetti, 1911

4414D487-E075-55BF-9866-68C7DD42E981

##### Notes

Qu (2001)

#### Stratiomyiidae

2F0352EA-8AA2-52FB-879C-A7CB0308AA8D

#### Hermetia

Latreille, 1804

C5B35C97-5BC3-5C74-B7A1-146F39674169

#### Hermetia illucens

(Linnaeus, 1758)

5011D172-D5CB-5F47-99B5-E69FFB32386A

##### Notes

DSPA (2022)

#### Streblidae

6D15239C-C094-5DEF-B329-ED91D0298783

#### Raymondia

Frauenfeld, 1856

F1015CF1-824D-5FF8-BFD3-A2039FBBAEF0

#### Raymondia pagodarum

Speiser, 1900

8CEE293E-6835-561A-8F05-7BD2E96BEC64

##### Notes

Easton (1992)

#### Syrphidae

E214FCFB-70E3-5B84-838C-9D1944326FE8

#### Allobaccha

Curran, 1928

8DD969EF-5787-5376-B107-1F0B7F623278

#### Allobaccha apicalis

(Loew, 1858)

16A71B72-B48A-5F42-ADAF-5FFD7613E21B

##### Notes

DSPA (2022)

#### Asarkina

Macquart, 1842

B4071E50-A7C9-5FCA-B4CF-C7B78D95FDBA

#### Asarkina porcina

(Coquillett, 1898)

A62A4721-737B-5B27-86D0-532CDB9176BD

##### Notes

DSPA (2022)

#### Episyrphus

Matsumura & Adachi, 1917

EF162294-4ADF-5BBD-9277-2A39470F7053

#### Episyrphus balteatus

(Geer, 1776)

9C945CFA-DCA6-5EA4-B2FD-C17EB9DD3665

##### Notes

DSPA (2022)

#### Eristalis

Latreille, 1804

2E00BA09-6FFC-5C19-AC20-7B2309CDD060

#### Eristalis tenax

(Linnaeus, 1758)

8E3734A1-9AC7-5DBE-A89F-D43CFDD92ADE

##### Notes

Easton (1993)

#### Eristalinus

Rondani, 1845

311CF9B2-63A9-5D29-990A-37BD8C050B4C

#### Eristalinus megacephalus

(Rossi, 1794)

694C4A02-C685-5778-8F9B-D8B2DFC86677

##### Notes

Easton (1993)

#### Helophilus

Fabricius, 1805

84D59854-5A6A-50D5-B6E0-8D86A8C5BEF3

#### Helophilus pendulus

(Linnaeus, 1758)

561DE332-BFE9-504A-B2B8-C1C542A68340

##### Notes

DSPA (2022)

#### Ischiodon

Sack, 1913

3BF698A3-90CB-5674-8E9E-0FB7A8E4CEEC

#### Ischiodon scutellaris

(Fabricius, 1805)

A922C03D-6273-5268-A8F6-A314925B3595

##### Notes

DSPA (2022)

#### Phytomia

Guérin-Ménéville, 1833

49513708-2DAF-5D82-A8E4-AD594DEBED75

#### Phytomia errans

(Fabricius, 1787)

5F69295C-9ADF-579D-8AE8-A9FA76C3FD34

##### Notes

DSPA (2022)

#### Scaeva

Fabricius, 1805

8C85EC25-6808-51C0-A144-83225BE24E49

#### Scaeva selenitica

(Meigen, 1822)

8E09AD5F-A8F4-5277-A2E5-D91DDCE8270A

##### Notes

Pun and Batalha (1997)

#### Syritta

Lepeletier & Serville, 1828

1C40957B-AD73-5F5E-AFF5-C2033AD8249B

#### Syritta orientalis

Macquart, 1842

F49F5693-0D28-53CD-9598-BFDCC6E05C6E

##### Notes

Pun and Batalha (1997)

#### Tabanidae

DFDAD374-43D0-5F65-94B2-EA0349B48123

#### Chrysops

Meigen, 1803

E82626C5-26A4-53C0-94F2-0C0302C30029

#### Chrysops vanderwulpi

Kröber, 1929

E5EBD1B7-FF65-5361-B463-9F21F9F32B1B

##### Notes

Yang et al. (2018)

#### Tachinidae

7CB0D6E1-4258-5430-B021-98514C578FAB

#### Cuphocera

Macquart, 1845

659D7CD4-4C88-5D4E-B0B3-BDF9318E6A74

#### Cuphocera varia

(Fabricius, 1794)

3C25DA49-2B9A-511D-9E5F-5E71701D0F81

##### Notes

DSPA (2022)

#### Tephritidae

FC945499-BF0F-55CD-B290-118138AD6756

#### Bactrocera

Macquart, 1835

64AE6D07-01DB-5C51-A7FC-9577E32BC5DA

#### Bactrocera cucurbitae

(Coquillett, 1899)

2DB0CE9D-CEFA-5FEB-9BDE-414D3A75B5F6

##### Notes

Pun and Batalha (1997)

#### Bactrocera dorsalis

(Hendel, 1912)

19665A95-DF45-5096-8B47-91138AF0D23F

##### Notes

Pun and Batalha (1997)

#### Bactrocera latifrons

(Hendel, 1912)

B98C2FBE-07EB-5F26-9C99-96110EF4A3A0

##### Notes

DSPA (2022)

#### Bactrocera tau

(Walker, 1849)

685E68F0-2377-5360-9101-7F30DCCFA8AF

##### Notes

Pun and Batalha (1997)

#### Diarrhegma

Bezzi, 1913

5003AA77-77EB-55A4-81DA-305C2FF02A81

#### Diarrhegma modestum

(Fabricius, 1805)

EA8B91CE-B2A9-5E35-88AD-E0DE6370E786

##### Notes

Pun and Batalha (1997)

#### Odonata

09016932-91DE-5B6B-804B-8FAA34EDE26C

#### Aeshnidae

78743D46-2586-5C23-9096-1B1AF76030BE

#### Anaciaeschna

Selyss, 1878

75A8C090-7048-5618-B8E9-D3EF394032BC

#### Anaciaeschna jaspidea

(Burmeister, 1839)

2B66C545-5714-5E62-BE8A-9AE8FE43BB17

##### Notes

Easton and Liang (2000)

#### Anax

Leach, 1815

553ADFDB-06AD-5783-8C92-00D336F30F83

#### Anax guttatus

(Burmeister, 1839)

CF6DD582-22C1-5AAF-BF6A-85FECD79D389

##### Notes

Li et al. (2015)

#### Anax immaculifrons

Rambbur, 1842

88852EFD-B937-5BE8-AAE1-A0E8A9D0C996

##### Notes

Easton and Liang (2000)

#### Anax parthenope

(Selys, 1839）

13B7FED1-0100-5DA1-97D4-2296DE0632DA

#### Anax parthenope julius

(Brauer, 1865)

07FA51B3-2E85-595A-BEA5-A0F406ED43EA

##### Notes

Easton and Liang (2000)

#### Gynacantha

Rambur, 1842

C9922CDA-3CA3-579D-96AA-8BF9161BECB5

#### Gynacantha subinterrupta

Rambur, 1842

61A2BAFE-A6D1-5854-9734-B6A16B94B4BB

##### Notes

Li et al. (2015)

#### Coenagrionidae

DD044712-26BB-5F6A-8A78-F39747F5CBBF

#### Agriocnemis

Selys, 1869

BC99345F-3E13-5258-9668-A813B9E5C962

#### Agriocnemis femina

(Brauer, 1868)

8EE2E18B-793E-50D9-B567-B6F4A2615306

#### Agriocnemis femina oryzae

(Lieftinck, 1962)

45A91402-CE84-59DD-A079-2810F18B9B2D

##### Notes

Li et al. (2015)

#### Agriocnemis pygmaea

(Rambur, 1842)

41E3DF8A-9704-5625-8CA6-3F184AB30CF3

##### Notes

Li et al. (2015)

#### Ceriagrion

Selys, 1876

C1EADF41-A82A-5E8C-82C2-036F9933CC8C

#### Ceriagrion aeruginosum

(Brauer, 1869)

2CF92C9E-386A-561B-B681-088F4751E6F4

##### Notes

Li et al. (2015)

#### Ceriagrion auranticum

Fraser, 1922

F44239E9-1426-566E-B951-3CC8131A4E40

#### Ceriagrion auranticum ryukyuanum

Asahina, 1967

53878E1F-F307-5249-A7F0-96CF256416F7

##### Notes

MBD (2022)

#### Cercion

Navas, 1907

AC4CF5E9-24E7-5F10-9010-75235BCA5CBF

#### Cercion sexlineatum

(Selxy, 1833)

89F5864C-3D34-5593-B7FD-F57548AF9C4F

##### Notes

Easton and Liang (2000)

#### Cercion melanotum

(Selys, 1876)

009ED8AA-DDB7-5E67-B9F4-3377B2A70DFB

##### Notes

Li et al. (2015)

#### Ischnura

Charpentier, 1840

FC976313-A202-589B-BC74-D201F8EE1E80

#### Ischnura aurora

Brauer, 1865

B79AE6EC-7060-5B35-9E73-CBA288E2DE3F

##### Notes

Wilson and Xu (2007)

#### Ischnura senegalensis

(Rambur, 1842)

236CE67A-262B-5812-8059-A2ADECB5624E

##### Notes

MBD (2022)

#### Mortonagrion

Fraser, 1920

A5C6ECC3-9BED-58EC-91F3-FE3927FFC5B7

##### Notes

MBD (2022)

#### Mortonagrion hirosei

Asahina, 1972

8D733AF3-603F-518E-BD4B-79463F5AB9CD

##### Notes

Pun and Batalha (1997)

#### Paracercion

Weekers & Dumont, 2004

8CB2A583-615E-53CF-8DD4-C09B78B6FB9E

#### Paracercion calamorum

(Ris, 1916)

8A2F4D22-8D79-5763-AF2E-156545C38172

#### Paracercion calamorum dyeri

(Fraser, 1920)

2DB216CC-B91A-54B8-AEA3-0A49BCF7BB0E

##### Notes

Wilson and Xu (2007)

#### Gomphidae

B6ABD183-4035-5E83-B422-97397469F19E

#### Ictinogomphus

Cowley, 1934

ED6035C3-EE8C-5186-924D-5D4CF05B210C

#### Ictinogomphus pertinax

(Selys, 1854)

27CCCFAA-BD3B-5061-AD2A-DA60476601B8

##### Notes

Li et al. (2015)

#### Sinictinogomphus

Frasers, 1939

524A2F6C-9C04-5474-AB3B-B423825795A1

#### Sinictinogomphus clavatus

(Fabricius, 1775)

2D9400FA-5151-565E-90BE-29A7BAC529E3

##### Notes

Li et al. (2015)

#### Libellulidae

9C4B8302-AC74-59DE-808E-B165C36146D9

#### Acisoma

Rambur, 1842

F52BFF7E-D6E7-5059-AA68-F30B2F72FCA4

#### Acisoma panorpoides

(Rambur, 1842)

BC034DC1-5EF8-5D13-9FD8-4592E2AFFEDE

##### Notes

Li et al. (2015)

#### Brachydiplax

Brauer, 1868

99154C81-DDE8-5D0A-BD3A-07954C130F82

#### Brachydiplax chalybea

Brauer, 1868

8F79B9BB-29E9-59BB-96D0-65488FDEA999

#### Brachydiplax chalybea flavovittata

Ris, 1911

F94B75B0-CAB4-536F-A107-B73C0D2F7381

##### Notes

Li et al. (2015)

#### Brachythemis

Brauer, 1868

2A785F17-8817-5DE8-982B-579719988B76

#### Brachythemis contaminate

(Fabricius, 1793)

67CCC576-4C1A-5637-9B54-8ACC86E2FE93

##### Notes

Li et al. (2015)

#### Crocothemis

Brauer, 1868

2372B786-5D15-5E7C-8663-1C366E274161

#### Crocothemis servilia

(Drury, 1773)

25470182-D45C-531D-87BA-4D761A93D090

##### Notes

Li et al. (2015)

#### Diplacodes

Kirby, 1889

2101194B-7146-5FC1-A505-EA2051EDDE1E

#### Diplacodes trivialis

(Rambur, 1842)

81F3754A-BEB8-5B41-B56B-61081B4DC753

##### Notes

Li et al. (2015)

#### Hydrobasileus

Kirby, 1889

B58FB42D-5909-5316-8E0D-E9C0341B8930

#### Hydrobasileus croceus

(Fraser, 1927)

AEAC7154-B5F0-58A2-871D-48DDF982122C

##### Notes

Li et al. (2015)

#### Lyriothemis

Brauer, 1868

2D8EDAC3-862D-5D78-9E0F-34ECA6D73F8D

#### Lyriothemis elegantissima

Sely, 1883

77439D10-D6C5-5FFF-8757-EB2FA675EFF7

##### Notes

Li et al. (2015)

#### Macrodiplax

Brauer, 1868

DA66477B-5CDB-5B8F-85CB-AAE3535C3760

#### Macrodiplax cora

(Brauer, 1867)

96B57A97-C580-5DC2-9283-BFA3935AB5C4

##### Notes

Li et al. (2015)

#### Neurothemis

Brauer, 1867

A1FAA144-2739-5FA5-8D2A-6BCFE052F0CE

#### Neurothemis tullia

(Drury, 1773)

ACE86827-1FB7-5F8C-B5F7-BACC65CEC18E

#### Neurothemis tullia tullia

(Drury, 1773)

1BDC3038-F913-548C-B63A-82E6E70DF509

##### Notes

Li et al. (2015)

#### Orthetrum

Hagen, 1861

8F8106EB-4C33-5543-95CF-04B2DDDD8059

#### Orthetrum chrysis

(Selys, 1891)

7DB39295-A0B6-5F91-97A9-6CE1B571156F

##### Notes

MBD (2022)

#### Orthetrum glaucum

(Brauer, 1865)

F378C481-9E3C-51EC-92DB-572FF1EDD3D7

##### Notes

Li et al. (2015)

#### Orthetrum luzonicum

(Brauer, 1868)

0352CE57-E09A-5A28-87C1-A7E88B52FA9A

##### Notes

Li et al. (2015)

#### Orthetrum melania

(Selys, 1883)

C0AD606B-FFD8-5889-A1FB-1B47899F27B2

##### Notes

Easton and Liang (2000)

#### Orthetrum pruinosum

(Burmeister, 1839)

8F96BD5B-AF2C-5432-885F-D30B7DCAC844

#### Orthetrum pruinosum neglectum

(Rambur, 1842)

C66BF765-19C4-5CA8-9D12-CE0CF36224FC

##### Notes

Li et al. (2015)

#### Orthetrum sabium

(Drury, 1770)

681D7EBD-954D-55CB-B404-4D1F6AD3E7BA

##### Notes

Li et al. (2015)

#### Pantala

Hagen, 1861

AB9C1E93-493A-5954-889F-CD4AC9985AA7

#### Pantala flavescens

(Fabricius, 1798)

89837929-D222-533C-8715-DC9F6F55E38C

##### Notes

MBD (2022)

#### Pseudothemis

Kirby, 1889

5BD1AE75-B451-5E7B-B1A9-8BE018104372

#### Pseudothemis zonata

(Burmeister, 1839)

BC05D742-A905-5E50-A60E-8ED7EFE7AC69

##### Notes

Li et al. (2015)

#### Rhyothemis

Hagen, 1867

5582459E-3515-5456-85D6-D4EB2EFCEEEC

#### Rhyothemis variegate

(Linnaeus & Johansson, 1763)

737E2A17-D498-5DAF-97CA-FA721A51ED94

##### Notes

Li et al. (2015)

#### Rhyothemis fuliginosa

Selys, 1883

1BC78E04-DF16-5446-AE49-680BCA6D76A3

##### Notes

Li et al. (2015)

#### Rhyothemis triangularis

(Kirby, 1889)

A128062F-1DD6-5E34-83AA-07674A30EB1B

##### Notes

Li et al. (2015)

#### Tholymis

Hagen, 1867

828D639F-B605-5E77-B11C-10FE0B0C520D

#### Tholymis tillarga

Fabricius, 1798

4899D822-F73A-51A9-83F4-8EBFDA1F41C8

##### Notes

DSPA (2022)

#### Tramea

Hagen, 1886

DCB9A547-D77E-5401-8CB8-2FFD6F0E3C9E

#### Tramea virginia

(Rambur, 1842)

01F10E60-927A-5ABA-9CAD-B71AAFF5E35E

##### Notes

Li et al. (2015)

#### Trithemis

Brauer, 1868

B051C6A3-2F39-571B-B701-08AC264D8458

#### Trithemis aurora

(Burmeister, 1839)

F2F75365-D56E-5AAA-984D-967FFE7F6ED3

##### Notes

Li et al. (2015)

#### Trithemis festiva

(Rambur, 1842)

418A0C2D-1876-59B7-92F9-236825AA8962

##### Notes

Li et al. (2015)

#### Urothemis

Brauer, 1868

AAB46686-7343-5F5C-81F6-0F0EB1FF4CE1

#### Urothemis signata

(Rambur, 1842)

7EF22125-F5A6-51EA-8F83-11955B80B81C

##### Notes

Li et al. (2015)

#### Zyxomma

Rambur, 1842

C2748EFC-4313-506E-BBE5-741AF4FB6602

#### Zyxomma petiolatum

Rambur, 1842

9BFFD4D1-11F3-53AC-B2A0-221CD7E831B8

##### Notes

Li et al. (2015)

#### Zygonyx

Hagen, 1867

0F6A27CE-4393-57B8-ABC2-D1DEFACC2AB8

#### Zygonyx iris

Selys, 1869

198E7D05-8C72-5D42-97EC-6578D65C6809

#### Zygonyx iris insignis

(Kirby, 1900)

31604E5A-9C65-54BD-A785-92999570FCAD

##### Notes

Li et al. (2015)

#### Platycnemididae

BEFA7B47-E166-5544-B91F-4BE86F2BC440

#### Copera

Kirby, 1890

FA39718C-CB54-5C51-B625-B22F311D4887

#### Copera ciliate

(Selys, 1863)

09659A0E-B55D-501B-81A6-988F149E327E

##### Notes

Li et al. (2015)

#### Copera marginipes

(Rambur, 1842)

F9A800DB-8AB1-5F2D-9442-B68F760C2BBE

##### Notes

MBD (2022)

#### Protoneuridae

CAEB2353-6904-50A2-985E-7024D003626E

#### Prodasineura

Cowley, 1934

BF6572FF-392D-5075-A7B3-BE489A30168C

#### Prodasineura autumnalis

(Fraser, 1922)

BE31B454-FEBF-54DB-9514-DF675A1CFCA5

##### Notes

Easton and Liang (2000)

#### Prodasineura nigra

(Fraser, 1922)

E85D2B4E-DBEA-5B09-A81E-A44ADD99A116

##### Notes

Li et al. (2015)

#### Prodasineura verticalis

(Selys, 1860)

0D8E2E83-733B-5186-98DE-4CCBB3B9409B

##### Notes

Easton and Liang (2000)

#### Orthoptera

022DFF09-DADD-5E2F-B689-257840205222

#### Acrididae

C9273C27-0876-5B81-8D4A-57831017D5AE

#### Acrida

Linnaeus, 1758

EF8B04BE-F130-566B-9F11-48B384BFF52D

#### Acrida cinerea

(Thunberg, 1815)

DD990CC4-F0A7-5BE1-9F34-57DA9C3A4893

##### Notes

Pun and Batalha (1997)

#### Gelastorhinus

Brunner-Wattenbwyl, 1893

A4E65FA2-9EA9-598E-9097-C41DF4B5BBBA

#### Gelastorhinus chinensis

Willenmse, 1932

B40827FC-DB25-5CF0-8E9A-C76645D4EC64

##### Notes

Liang and Easton (1995)

#### Gonista

Bolivar, 1898

A247FF68-A3B3-51AC-9503-342F23D83E9B

#### Gonista bicolor

(Haan, 1842)

ED292095-2B36-56A6-8C20-1AB8FC697A2F

##### Notes

Liang and Easton (1995)

#### Phlaeoba

Stål, 1860

FC2C9504-837F-5CAE-BBC7-4594BC1AFA74

#### Phlaeoba antennata

Brunner-Wattenbwyl, 1893

F49190AB-EF36-5128-BA02-8F9E93633845

##### Notes

Liang and Easton (1995)

#### Arcypteridae

64740649-05DC-522F-8742-39D6D529A0AB

#### Ceracris

Walker, 1870

9F45D811-8F47-568C-9EBF-6F304E41528F

#### Ceracris fasciata

(Brunner-Wattenwyl, 1893)

18D1FD94-2550-5C50-A30C-51BEE6BABC87

##### Notes

Pun and Batalha (1997)

#### Catantopidae

A1D03409-889F-5ED1-A61A-8440BE3EBB84

#### Apalacris

Walker, 1870

0EDE4CE6-0C5A-5C67-A18E-1ADF8436C033

#### Apalacris nigrogeniculata

Bi, 1984

0906FF0C-41A7-5853-B3F4-9B6684F21D1E

##### Notes

Liang and Easton (1995)

#### Chondracris

Uvarov, 1923

8276CFE9-45BD-56D9-94D3-256327DE3394

#### Chondracris rosea

(Geer, 1773)

9676FF3A-F3E8-5829-BC55-A4E75B51BA71

##### Notes

Liang and Easton (1995)

#### Epistaurus

Bolivar, 1889

4919EB6D-2284-570D-AE1C-A4554AACD4A6

#### Epistaurus aberrans

Brunner-Wattenwyl, 1893

23D12F3F-5259-51AE-A902-8130047DEF1A

##### Notes

Liang and Easton (1995)

#### Eucoptacra

Bolivar, 1902

9D76C8C9-C5EB-52BE-B8ED-208CE866D98B

#### Eucoptacra praemora

(Stål, 1860)

B393682B-37E1-53C0-9EB8-04B83F28A75E

##### Notes

Liang and Easton (1995)

#### Eyprepocnemis

Fieber, 1853

8788B0E3-4E58-59EC-81F3-8A921B8E4761

#### Eyprepocnemis hokutensis

Shiraki, 1910

B5C47F22-3019-55D3-A52F-E0FA1FF71A25

##### Notes

Liang and Easton (1995)

#### Gesonula

Uvarov, 1940

C846738F-DF0F-58A2-97EC-1060BF15AA75

#### Gesonula punctifrons

(Stål, 1861)

78B79298-264E-582C-888F-258149C58B34

##### Notes

Liang and Easton (1995)

#### Hieroglyphus

Krauss, 1877

F70226A6-C355-5146-A611-D84ED3CEC297

#### Hieroglyphus annulicornis

(Shiraki, 1910)

E25FC59F-F40D-51AE-A257-33A0E463E8D0

##### Notes

Pun and Batalha (1997)

#### Oxya

Audinet-Serville, 1831

24CF49D0-0F21-53D6-88A2-845B7EE6585A

#### Oxya chinensis

(Thunberg, 1815)

D391D770-052A-5B67-B75E-C543F9EC8F21

##### Notes

Liang and Easton (1995)

#### Oxya intricate

(Stål, 1861)

C8ED6BE0-23BA-5764-91E1-C4B8B4040C2B

##### Notes

DSPA (2022)

#### Patanga

Uvarov, 1923

86E9246B-29DC-5EF9-891C-468F16D5F76A

#### Patanga succincta

(Johansson, 1763)

5CB59CC4-0715-5F07-B53A-BE14FF0C0BD1

##### Notes

Pun and Batalha (1997)

#### Pseudoxya

Yin & Liu, 1975

5254C3E8-CBD6-585C-A68E-E8FA25885422

#### Pseudoxya dimnuta

(Walker, 1871)

E843C839-AA51-59A5-ADC4-60EA18EA8297

##### Notes

Pun and Batalha (1997)

#### Stenocatantops

Dirsh, 1953

AFCCD0BE-A038-5C99-9B1B-92122F432D3C

#### Stenocatantops splendens

(Thunberg, 1815)

8AFB79B9-ACB3-5ED0-9899-621DC96C792D

##### Notes

Liang and Easton (1995)

#### Traulia

Stål, 1873

6FD1C55E-4EB3-580D-9857-BCC2020052F4

#### Traulia lofaoshana

Tinkham, 1940

83B9E867-7F79-5D42-B43F-0EE90A81B64E

##### Notes

Liang and Easton (1995)

#### Tristria

Stål, 1873

F8650A4D-87AC-5DE8-B8E6-FD32F492D68E

#### Tristria pisciforme

(Audinet-Serville, 1839)

154F11CD-2D2A-5821-9674-4B0476F86EB6

##### Notes

Liang and Easton (1995)

#### Gryllacridae

16137AA1-5F49-5F78-9212-B9639F3C3B39

#### Phryganogryllacris

Karny, 1937

0F246EBC-0F11-5AE4-961A-D54506D3C52D

#### Phryganogryllacris mellii

(Karny, 1926)

03EA3E72-9B7F-5B50-8A62-55565F1FA2A8

##### Notes

Pun and Batalha (1997)

#### Gryllidae

EE7A95D5-2DC7-5461-8114-4084869FD541

#### Gryllodes

Saussure, 1874

C5DA0008-BBD7-5D61-968C-AF94813F929C

#### Gryllodes sigillatus

(Walker, 1869)

B90B8F42-671D-510C-A662-A0FAD9C20112

##### Notes

DSPA (2022)

#### Tarbinskiellus

Gorochov, 1983

678997D6-D248-50A1-92FC-D8370A00D6EF

#### Tarbinskiellus portentosus

(Lichtenstein, 1796)

EA2F2FED-1D26-5CF7-AC1F-436E225C3527

##### Notes

Pun and Batalha (1997)

#### Teleogryllus

Chopard, 1961

8621D674-1293-57F4-ADAD-3CA891CECCC0

#### Teleogryllus mitratus

(Burmeister, 1838)

A5253644-08CA-5902-B073-2571C88CD9B8

##### Notes

Pun and Batalha (1997)

#### Gryllotalpidae

68ED4A96-B101-58DD-8B8E-EB13D38FF153

#### Gryllotalpa

Latrille, 1802

FEBF0F99-8402-582F-96A9-DD9A27428E22

#### Gryllotalpa africana

(Palisot de Beauvois, 1805)

553DA9AB-6BB8-5860-AB06-8DCBDC644B76

##### Notes

Easton (1991)

#### Gryllotalpa orientalis

Burmeister, 1839

B433815F-24EF-5156-8F02-E9F7F8CDCE92

##### Notes

Pun and Batalha (1997)

#### Oedipododae

E59C0A21-B46B-54E6-A17A-6EE5A58B7891

#### Aiolopus

Fieber, 1853

EA142D70-EC93-5F5B-848F-EA6DACB4C7A1

#### Aiolopus tamulus

(Fabricius, 1798)

A64819D6-B1DA-5F34-BDE0-6E78A352318F

##### Notes

Pun and Batalha (1997)

#### Gastrimargus

Saussure, 1884

544E96AE-7771-594B-BC54-37EA10817363

#### Gastrimargus marmoratus

(Thunberg, 1815)

E08E0DB4-8CF2-5599-9D52-E685718119D5

##### Notes

Pun and Batalha (1997)

#### Heteropternis

Stål, 1873

A027A7F0-0FDC-5541-A960-CE1A6A6BDB5E

#### Heteropternis respondens

(Walker, 1859)

60C985B9-C04F-5093-B549-19240E60EAD1

##### Notes

Pun and Batalha (1997)

#### Heteropternis rufipes

(Shiraki, 1910)

945607B2-3512-5B0F-835F-85997977E2F4

##### Notes

Liang and Easton (1995)

#### Locusta

Linnaeus, 1758

0606C5DB-B8E0-587C-9BEC-BE73D54D2F52

#### Locusta migratoria

(Linnaeus, 1758)

D7DA0252-E0F5-5521-9628-838C4B719C55

##### Notes

Mo et al. (2006)

#### Trilophidia

Stål, 1873

98D58110-461F-52A8-99E0-C05EDCE81735

#### Trilophidia annulata

(Thunberg, 1815)

B0CD29BF-F98C-5831-9DC2-B647917262D2

##### Notes

Pun and Batalha (1997)

#### Pyrgomorphidae

36BC61B1-5927-56C6-B668-9700BEE4565B

#### Atractomorpha

Saussure, 1862

C7306FEB-D56E-5802-B7A9-B808AC10CB1D

#### Atractomorpha sinensis

Bolivar, 1905

EAB1FFB4-DEC5-5254-B699-4C303ECEC774

##### Notes

Pun and Batalha (1997)

#### Scelimenidae

2F77408C-FEED-5AEB-BD96-29CD77FD659A

#### Criotettix

Bolivar, 1887

E4AEFCA9-C3B6-5431-A1A7-206718BFD06D

#### Criotettix japonicas

(Haan, 1842)

1DB6072E-48B7-559B-A561-79451BEF71DC

##### Notes

Teng et al. (2022)

#### Tetrigidae

167795CD-E142-52D2-995A-17BDC693152D

#### Euparatettix

Hancock, 1904

DF5E2F5E-B118-5FE2-88AE-4EA2A8FD5656

#### Euparatettix variabilis

(Bolívar, 1887)

205EA23C-A911-56E6-BBD1-E669E6928D2C

##### Notes

DSPA (2022)

#### Hedotettix

Bolivar, 1887

3429B451-D39F-54F1-A84F-BCDFF4DA4CAD

#### Hedotettix gracilis

(Haan, 1843)

2CB0D334-3760-5F5B-A45C-337104589F24

##### Notes

DSPA (2022)

#### Hyboella

Hancock, 1915

D58AA36E-7603-58AA-B132-D654EE474BE8

#### Hyboella longinota

Zheng & Jiang, 2002

44F06DAD-2324-5554-96D8-225C7ADEFAC8

##### Notes

DSPA (2022)

#### Tetrix

Latreille, 1802

E0B5DCB8-0F03-51CF-9A13-205DDB554D45

#### Tetrix japonica

(Bolívar, 1887)

FCCCAC71-3360-5E2F-BD08-2FA04A2BCA3B

##### Notes

DSPA (2022)

#### Thoradonta

Hancock, 1909

99573681-E970-5FF9-AD16-7DD763808FA8

#### Thoradonta spiculoba

Hancock, 1912

1F5D6272-4BFC-5B88-9DDC-1B4E8E3D4B80

##### Notes

DSPA (2022)

#### Tettigoniidae

790A78D5-0F16-59B5-9BB3-9C473614FEFB

#### Conocephalus

Thunberg, 1815

0F7A3C59-CC5F-5669-99CB-7BEBCEB56003

#### Conocephalus maculates

(Le Guillou, 1841)

F8C50CF7-F23C-5BD9-AA07-C005E283508A

##### Notes

DSPA (2022)

#### Elimaea

Stål, 1874

CFF5040C-5211-5168-85FD-625DC4F8AD30

#### Elimaea punctifera

(Walker, 1869)

E03244F9-CA25-560D-976A-9605073A5D56

##### Notes

Pun and Batalha (1997)

#### Euconocephalus

Karny, 1907

04DBAAF6-1232-5D73-95FB-C6515982D023

#### Euconocephalus varius

(Walker, 1869)

CFDC29FD-264E-552E-8CEB-4D8C79CEC775

##### Notes

DSPA (2022)

#### Hexacentrus

Serville, 1831

84D8475F-B29F-5579-9D89-6CAEE4A14A67

#### Hexacentrus unicolor

Serville, 1831

48ECBB6C-7BDC-56C5-85FB-42AF4CD29F83

##### Notes

Pun and Batalha (1997)

#### Holochlora

Stål, 1873

3E3E027B-27E0-541F-95DB-7E1988BF5B83

#### Holochlora japonica

Brunner, 1878

6BCBD0F3-E8F5-5CA3-8AB4-41287125655D

##### Notes

Pun and Batalha (1997)

#### Mecopoda

Audinet-Serville, 1831

86D59FC6-E19D-5A72-A1DC-DF6FA7977EE1

#### Mecopoda elongate

(Linnaeus, 1758)

4A99E35F-03FA-5B1B-93F3-5A7ACFEAEDB1

##### Notes

MBD (2022)

#### Sinochlora

Tinkham, 1945

000B11DF-B3ED-5814-9C8F-CDEA060A6CAE

#### Sinochlora longifissa

(Matsumura & Shiraki, 1908)

B86B7618-ADD7-5CD1-B33F-C424E4D9D9EB

##### Notes

Mo et al. (2006)

#### Tridactylidae

3EB1AB08-1E25-5DD8-B593-02537920F43A

#### Xya

Latreille, 1809

634C68D0-449B-50CF-BDE7-A566977AA26D

#### Xya japonica

(Haan, 1844)

41236756-59BE-5342-9BF6-03D0C805B1C9

##### Notes

DSPA (2022)

#### Blattodea

676CFF79-60F0-52F6-9E18-4C0C216A0801

#### Blattidae

5E5F3031-CDB9-5E9B-87E2-34E6648D743D

#### Opisthoplatia

Brunner-Wattenwyl, 1865

51470DA7-9890-5FB6-AA61-93BC5D4A9BF7

#### Opisthoplatia orientalis

(Burmeister, 1838)

F9EBF202-E7FD-5EE1-A2A7-755316F62A10

##### Notes

Pun and Batalha (1997)

#### Periplaneta

Burmeister, 1838

4D539FEA-0547-58D1-90DB-5906174A03A2

#### Periplaneta americana

(Linnaeus, 1758)

BA9AF0AC-65FC-5CD0-B94A-BB9FBEE56C7F

##### Notes

Easton (1993)

#### Ectobiidae

AB2C6925-5341-5C41-B04A-A596B96FCAA3

#### Blattella

Caudell, 1903

0FF64098-C115-50C2-8F92-1FFF7EEC839B

#### Blattella bisignata

(Brunner von Wattenwyl, 1893)

47F5B921-9EFE-54C1-BB3D-A385927256FA

##### Notes

DSPA (2022)

#### Blattella germanica

(Linnaeus, 1767)

18C5B68A-20AD-5C60-9341-F2160D79EFC1

#### Kalotermitidae

044BBA77-C6CE-5DD8-860A-676425E224EB

#### Cryptotetmes

Banks, 1906

5A22902A-04A5-5267-8AEB-507D3D231728

#### Cryptotetmes declivis

Tsai & Chen, 1963

A5046EE8-EF4E-5844-85E4-00ED6FBCF223

##### Notes

MBD (2022)

#### Neotermes

Holmgren, 1911

8E76E0CD-0ABE-5AC0-9FCC-0319FDF93088

#### Neotermes tuberogulus

Xu & Han, 1985

72D3A735-1DED-573B-A3E0-9F1E9B9B0DC6

##### Notes

MBD (2022)

#### Rhinotermitidae

238436AF-A4F0-52CA-A959-6D49227623CA

#### Coptotermes

Wasmann, 1896

8DFE2806-53AF-5C7C-8D70-19D42B0AD2CF

#### Coptotermes formosanus

Shiraki, 1909

13E9F8C9-2BF4-5CF2-A3B4-43D53B1CB5C4

##### Notes

MBD (2022)

#### Termitidae

C007650F-5F28-5F36-8FA7-ED98B6165D0F

#### Macrotermes

Holmgren, 1912

81DD5C21-F166-57F1-A021-D23B0A1995D3

#### Macrotermes barneyi

Light, 1924

F3E7FFF5-0E28-5FAA-9419-549CCDCE9083

##### Notes

MBD (2022)

#### Odontotermes

Holmgren, 1912

286EFD2D-63B4-5E6F-95B0-330D987DCA37

#### Odontotermes formosanus

(Shiraki, 1909)

6B15F46D-2438-5022-BBBB-3697B77B570C

##### Notes

MBD (2022)

#### Odontotermes hainanensis

(Light, 1924)

0409FF8A-D12A-5137-ABE5-81A976D7EA38

##### Notes

MBD (2022)

#### Pericapritermes

Silvestri, 1914

44D9A1C5-27AB-561F-B475-CF490C2A640F

#### Pericapritermes jangtsekiangensis

(Kemner, 1925)

4E88A96D-51A1-5666-95C2-58CA49B77182

##### Notes

MBD (2022)

#### Pericapritermes nitobei

(Shiraki, 1909)

4BD53C01-4C81-502B-AAEB-859F51A9AD33

##### Notes

Li et al. (2018)

#### Pseudocapritermes

Kemner, 1934

4092A238-3F42-53F6-8F6D-9BB7E17705C4

#### Pseudocapritermes sowerbyi

(Light, 1924)

68B2E860-83F0-5069-AC17-417D5F144DAC

##### Notes

MBD (2022)

#### Sinocapritermes

Ping & Xu, 1986

3110F436-1F54-5EC0-A77A-FC4529B43ED1

#### Sinocapritermes mushae

(Oshima & Maki, 1919)

F1E00893-4BFA-5436-B6E7-58A8992E4DEF

##### Notes

MBD (2022)

#### Mantodea

B518F6E0-954B-5DD5-82B9-222866A8E60B

#### Chaeteessidae

EA4D828E-0DEB-5489-A14C-D77F53F6BDD7

#### Chaeteessa

Burmeister, 1838

0366D23F-56AE-59FB-B3DA-CEC74CE68AC9

#### Chaeteessa caudata

Saussure, 1871

6B1919F4-F14D-5665-92D4-FC8C3E308DDC

##### Notes

Heleodoro et al. (2016)

#### Mantidae

FFCD754F-614B-5AC9-9063-DCA54A39CE83

#### Statilia

Stål, 1877

3B60FE9E-662E-5CEC-9995-F3754894EE0B

#### Statilia maculata

(Thunberg, 1784)

FA122641-6BF8-5D76-87CC-184E7B824814

##### Notes

Pun and Batalha (1997)

#### Tenodera

Burmeister, 1838

D607772C-A1E2-5830-BC4D-D2B38CADA096

#### Tenodera sinensis

Saussure, 1871

77B1BB19-755F-5A83-BA71-31A3FDFB0CC3

##### Notes

Pun and Batalha (1997)

#### Photinaidae

94F79DF2-0F11-5877-BE7C-0188A21D4AA5

#### Photiomantis

Piza, 1968

D086AA50-68D0-5082-886F-BB44D234021C

#### Photiomantis planicephala

(Rehn, 1916)

490A81F9-E6BE-5046-8595-556F389ECFC0

##### Notes

Heleodoro et al. (2016)

#### Toxoderidae

F2F07F3C-C733-5690-8E17-522F57471463

#### Toxomantis

Giglio-Tos, 1914

E253E671-D43E-5391-9DAC-3BA18CC2B6B6

#### Toxomantis sinensis

Giglio-Tas, 1914

89C98F87-3CC5-564F-9879-DCF05DBE4775

##### Notes

Yang (1997)

#### Zygentoma

1A9E3A22-1B42-5779-9479-70EF2BA4DABF

#### Lepismatidae

C22DD614-E910-5979-9F88-6C9A8E4A3EDB

#### Lepisma

Linnaeus, 1758

8D911C03-B679-5E94-81B5-D7326DBB6EF7

#### Lepisma saccharina

Linnaeus, 1758

E4110250-35C3-59AC-85D7-72673317BAF1

##### Notes

Easton (1991)

#### Nicoletiidae

F2A7366C-1FEE-56E9-8C9F-56696C0DAC96

#### Gastrotheus

Casey, 1890

1BF9948D-A298-5044-9A1F-F8BC0ACB2F8B

#### Gastrotheus nanus

(Escherich, 1903)

47AC0935-72F5-515E-BF24-8C1CF04281D0

##### Notes

Mendes (2002)

#### Proatelura

Silvestri, 1916

9BE64DAC-1642-5505-B8E5-2DF7774D84E1

#### Proatelura jacobsoni

(Silvestri, 1911)

D6CE4438-0190-5A78-B91D-6BBCEF52FF54

##### Notes

Mendes (2002)

#### Neuroptera

3D5E46CA-84A1-52A1-B3CE-27E5DEFE9E58

#### Ascalaphidae

4DE936CA-3C84-5805-839E-0111F4EFFEF5

#### Ascalohybris

Sziraki G, 1998

78D0E38F-7C97-54CE-8B21-4B2C0CC6A5DF

#### Ascalohybris subjacens

(Walker, 1853)

39A05553-0601-5B7C-92F7-D4EE4950F370

##### Notes

Easton (1992)

#### Chrysopidae

89779C8E-A94A-5191-8A87-6DF2310CF94E

#### Chrysoperla

Steinmann, 1964

065E4ACE-9F03-51FB-9B4E-00773D03859B

#### Chrysoperla carnea

(Stephens, 1836)

9EBFB7A5-4D24-54CA-8F5B-E0E7368D8994

##### Notes

DSPA (2022)

#### Phasmatodea

E48D7217-3658-5AD2-B5DE-7FA8765ADFB4

#### Diapheromeridae

878FBF75-FE9D-5D0C-8A2B-0F47A4058F26

#### Macellina

Uvarov, 1940

4FA146F5-75B8-5731-B2B0-0E617F4C32B7

#### Macellina souchongia

(Westwood, 1859)

35CBAE92-02A8-5D38-9760-291F324EAF24

##### Notes

Chen and He (2008)

#### Dermaptera

2DBDEA9F-9109-5AC2-9CD3-01D2C80BBF9C

#### Labiduridae

572116BE-1C8C-57FC-A642-43C8B1A8D25A

#### Labidura

Leach, 1815

91F71A58-1BFB-5897-8772-E4D99F92935F

##### Notes

DSPA (2022)

#### Labidura riparia

(Pallas, 1773)

EFC152B1-5536-57FF-BF12-15E795687DE3

##### Notes

DSPA (2022)

#### Siphonaptera

B81D318A-1F40-55E9-8F4D-EFB99D23709C

#### Pulicidae

4704B3E6-37C6-532B-8A7D-CB708F21CEF3

#### Ctenocephalides

Stiles & Collins, 1930

AB2BCB61-AAF4-5636-94AB-30123680DFA9

#### Ctenocephalides felis

(Bouché, 1835)

47C1CBEF-9573-5EE9-90B4-0D1F87DFF32B

##### Notes

Easton (1991)

#### Thysanoptera

ED0235C7-31A2-58A3-9F34-FD6F0AF571E3

#### Phlaeothripidae

13EA2A68-1387-5B0F-B36F-9E6F8CA5047F

#### Gynaikothrips

Zimmermann, 1900

EEBF7EED-6FD7-5FF5-B984-A8D9973A6E85

#### Gynaikothrips ficorum

(Marchal, 1908)

17953F87-AE35-572E-9371-91295754AE29

##### Notes

DSPA (2022)

## Analysis

Twenty-six years after the initial catalogue compiled by Pun and Batalha (1997), here we provided an updated catalogue of insect species of Macao, which contains a sum of 15 orders, 166 families, 868 genera, 1,339 species and 118 subspecies (Table 2), including some endemic species, such as *Toxorhynchitesmacaensis* Ribeiro, 1997 and *Leptanillamacauensis* Leong, Yamane & Guénard, 2018. The exact number of endemic species is uncertain due to the lack of comprehensive distribution records for each species from Macao and surrounding areas.

### Conclusion

In summary, we collated all the insect species distributed in Macao and generated a list and carried out statistical analysis. (1) According to the new catalogue, Macao has 1,339 recorded insect species, which represents an increase of 888 species compared to those reported by Pun and Batalha in 1997; (2) The most conservative estimation of insect species in Macao, based on the list and the urban characteristics of Macao, is no fewer than 3,340, which is about 2.5 times of the currently known amount; (3) The current pattern is characterised by a relatively full investigation of ornamental insects and inadequate investigation of aquatic, parasitic and other cryptic insects.

## Discussion

**Figure 1.**
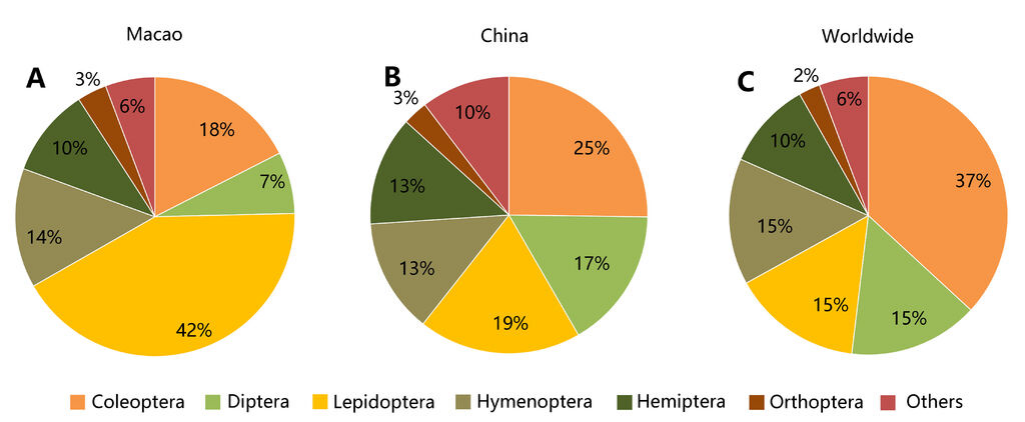
Number of species proportions by orders in different geographical scope types.

According to the new catalogue, the most abundant species distributed in Macao are Lepidoptera (565 species, 76 subspecies), followed successively by Coleoptera (233 species, 23 subspecies), Hymenoptera (185 species, 11 subspecies), Hemiptera (139 species), Diptera (94 species), Odonata (49 species) and Orthoptera (46 species) (Table 2; Fig. 1A). In comparison, the six orders with the largest species composition in the world or China are Coleoptera, Diptera, Lepidoptera, Hymenoptera, Hemiptera and Orthoptera (Fig. 1B, C).

**Figure 2.**
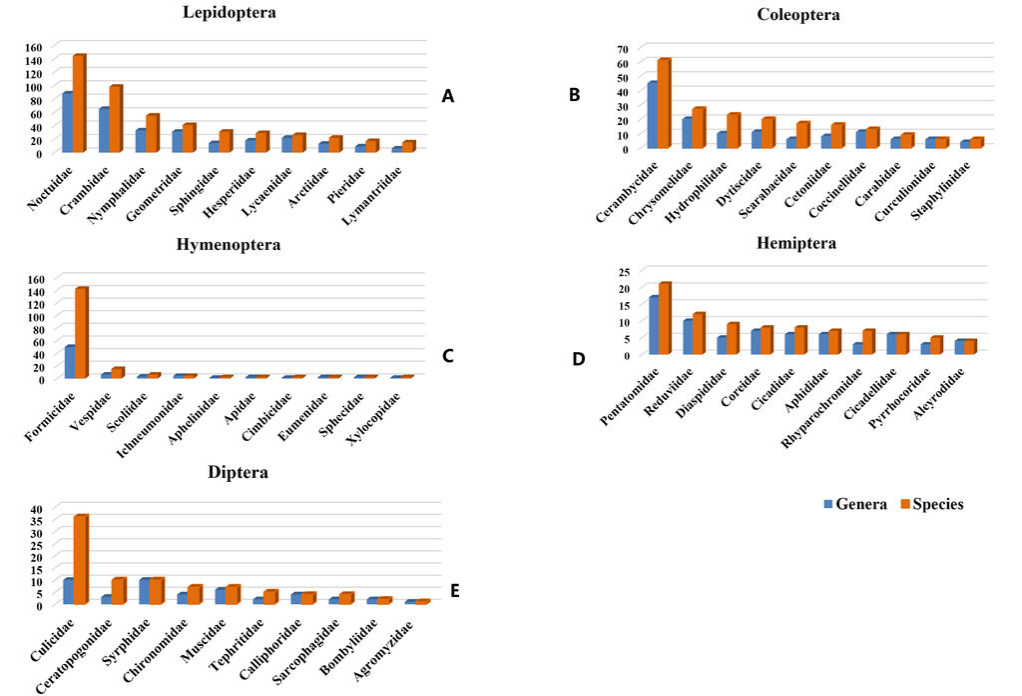
Dominant families with corresponding number of species and genera in Macao.

Lepidopterans include moths and butterflies, the third largest order after Coleoptera and Diptera worldwide. Within Lepidoptera, the family Noctuidae was the most speciose in the world or China, which is the same case in Macao (Fig. 2A). According to the current statistical results (Tables 2, 3), Lepidoptera is seemingly the most comprehensively investigated order in Macao (565 species).

As the largest insect order in the world and China, the currently recognised species number of beetles is the second in the fauna of Macao. The first four families with most recorded species in the world are Curculionidae, Carabidae, Cerambycidae, Tenebrionidae and are Curculionidae, Carabidae, Cerambycidae and Chrysomelidae in China (Shen 2015), whereas, the first four families with most recorded species in Macao are Cerambycidae, Chrysomelidae, Hydrophilidae and Dytiscidae (Table 4; Fig. 2B). The relatively abundant knowledge about the aquatic beetles is very likely due to the accumulated contribution made by Prof. Feng-long Jia, the fourth author of this work. Therefore, the species diversity of beetles in Macao is still inadequately understood. Considering the particularity of the local in Macao, the minimum species of Coleoptera in Macao should not be less than that of Lepidoptera, i.e. 565 species.

Hymenopterans include bees and ants and are one of the largest orders of insects in the world in terms of species diversity. Ichneumonidae and Braconidae have the most recorded species in the world or China (Shen 2015). In comparison, Formicidae has the most recorded species amongst all known insect families in Macao (Table 5; Fig. 2C) and accounts for 76% of the species number of Hymenoptera. The relatively abundant knowledge about the ants of Macao is very likely due to the accumulated contribution made by Dr. Chi Man Leong, the second author of this work. As for the parasitic wasps, only eight species have been recorded so far in Macao. Generally, considering that large quantity species of Coleoptera, Lepidoptera and Hemiptera can be parasitised by parasitic wasps, the species number of parasitic wasps in Macao is very likely underestimated. Using Lepidoptera as a reference, according to the ratio of Lepidoptera to Hymenoptera in China, it is estimated that there should be no less than 390 species of Hymenoptera in Macao.

As one of the most common insects, dipterans are the second largest order in the world, while it ranks as the fifth in Macao. The species number of Diptera is higher than that of Hymenoptera or Hemiptera in the world or China, but lower than that of Hymenoptera or Hemiptera in Macao. The known species of Diptera is approximately 1/6 of that of Lepidoptera in Macao. Within Diptera, Tachinidae and Tipulidae are the most recorded species in the world and Tachinidae and Culicidae are the most recorded species in China. The two families with most recorded species in Macao are Culicidae and Ceratopogonidae (Table 7; Fig. 2E). It seems that the investigation of dipteran in Macao is extremely inadequate. Additionally, the species number of dipterans in Macao is estimated to be no lower than 490 (the estimation method is the same as that of Hymenoptera).

Hemipterans are one of the largest insect orders in the world and China, the currently recognised species number of Hemiptera ranking as the fourth in the fauna of Macao. The largest family in the world or China are Cicadellidae and Miridae (Shen 2015). Amongst the current records in Macao, the most records for the family of Hemiptera is the family Pentatomidae with 20 species (Table 6; Fig. 2D). Besides, according to the collection of heteropterans in Guangdong and its surrounding areas, it is speculated that there are still some unrecorded families and many unrecorded species in Macao. Hence, the studies on this order in Macao is far from sufficient and there should be no less than 380 Hemiptera species in Macao (The estimation method is the same as that of Hymenoptera.).

Orthoptera, as the sixth largest order in the world, has approximately 26,107 species globally (Foottit and Adler 2017), with 2,716 species found in China (Shen 2015). However, Macao has only recorded a mere 46 species thus far (Table 8). Besides, Trichoptera (14,548) and Psocodea s. lat. (= Psocodea s. str. + Phthiraptera 10,879) also possess over 10,000 species worldwide and their species in China is also considerable, namely Trichoptera (927), Psocoptera (1, 648) and Phthiraptera (97), but none of these groups has been recorded in Macao. This further highlights the inadequacy of insect fauna surveys conducted in Macao. Reflected by the knowledge about aquatic beetles and dragonflies, the diversity of aquatic insects in Macao is relatively rich. Therefore, it is somewhat surprising that Trichoptera have not been reported in Macao. According to the ratio mentioned above, we estimate that there should be no fewer than 80 species of Orthoptera recorded in Macao, no fewer than 460 species of Trichoptera and no fewer than 340 species of Psocodea s. lat.

It is strange that there is no record of Ephemeroptera in Macao, while Ephemeroptera is also a widespread insect order, ranging from small streams to large lakes. Moreover, Megaloptera and Dermaptera are confronted with the same situation. Thus, Macao's biodiversity may be underestimated. At present, the fauna of Macao has the following characteristics: (1) only Lepidoptera is relatively well studied; (2) there are few known aquatic insects, except aquatic beetles and dragonflies; (3) there are few records of parasitic insects. According to the above conservative speculations, even considering the special characteristics of the local fauna of Macao, the insect species in Macao should not be less than 3,340 species.

## Figures and Tables

**Table 1.** Specific bibliography of Macao's insects in 1996–2023.

Author (year)	Order	Species
Easton and Pun (1996)	Lepidoptera	145
Easton and Pun (1997a)	Hemiptera	59
Easton and Pun (1997b)	Lepidoptera	1
Ribeiro (1997)	Diptera	1
Ramos et al. (1997)	Diptera	35
Easton and Liang (2000)	Odonata	27
Qu (2001)	Diptera	37
Ke and Mai (2003)	Diptera	1
Mo et al. (2006)	Orthoptera, Hemiptera, Coleoptera, Lepidoptera, Diptera	62
Yu and Huang (2006)	Diptera	7
Wilson and Xu (2007)	Odonata	8
Mendes and Sousa (2007)	Lepidoptera	75
Sun et al. (2010)	Diptera	2
Xiao (2012)	Blattodea	9
Li et al. (2015)	Odonata	35
Huang et al. (2016)	Lepidoptera	65
Leong et al. (2017)	Hymenoptera	113
Leong (2017)	Hymenoptera	1
Leong et al. (2018)	Hymenoptera	1
Wong and Guénard (2020)	Hymenoptera	6
Brassard et al. (2020)	Hymenoptera	9
Brassard et al. (2021)	Hymenoptera	126
Perissinotto and Clennell (2021)	Coleoptera	11
Lin et al. (2021)	Coleoptera	52
Perissinotto and Lu (2022)	Coleoptera	12
Perissinotto et al. (2023)	Lepidoptera	1
Li (2023)	Lepidoptera	368

**Table 2.** Statistics of insect species in Macao.

Order	Family	Genus	Species	Subspecies
Lepidoptera	31	364	565	76
Coleoptera	27	157	233	23
Hymenoptera	17	79	185	11
Hemiptera	38	116	139	0
Diptera	18	52	94	0
Odonata	6	32	49	8
Orthoptera	12	43	46	0
Blattodea	5	11	14	0
Mantodea	4	5	5	0
Zygentoma	2	3	3	0
Neuroptera	2	2	2	0
Phasmatodea	1	1	1	0
Dermaptera	1	1	1	0
Siphonaptera	1	1	1	0
Thysanoptera	1	1	1	0
Total：15	166	868	1,339	118

**Table 3.** Statistics of Lepidoptera in Macao.

Family	Genus	Species	Subspecies
Noctuidae	88	144	0
Crambidae	65	98	0
Nymphalidae	33	55	27
Geometridae	31	41	4
Sphingidae	14	31	6
Hesperiidae	18	29	13
Lycaenidae	22	26	10
Arctiidae	13	22	1
Pieridae	9	17	3
Lymantriidae	6	15	0
Others	65	87	12
Total：	364	565	76

**Table 4.** Statistics of Coleoptera in Macao.

Family	Genus	Species	Subspecies
Cerambycidae	45	61	13
Chrysomelidae	20	27	1
Hydrophilidae	10	23	0
Dytiscidae	11	20	3
Scarabaeidae	6	17	1
Cetoniidae	8	16	2
Coccinellidae	11	13	0
Carabidae	6	9	1
Curculionidae	6	6	0
Staphylinidae	4	6	0
Others	31	36	2
Total：	158	234	23

**Table 5.** Statistics of Hymenoptera in Macao.

Family	Genus	Species	Subspecies
Formicidae	50	142	6
Vespidae	6	15	3
Scoliidae	3	6	0
Ichneumonidae	4	4	0
Aphelinidae	1	2	0
Apidae	2	2	0
Cimbicidae	1	2	0
Eumenidae	2	2	0
Sphecidae	2	2	1
Xylocopidae	1	2	0
Others	7	6	1
Total：	79	158	11

**Table 6.** Statistics of Hemiptera in Macao.

Family	Genus	Species	Subspecies
Pentatomidae	17	21	0
Reduviidae	10	12	0
Diaspididae	5	9	0
Coreidae	7	8	0
Cicadidae	6	8	0
Aphididae	6	7	0
Rhyparochromidae	3	7	0
Cicadellidae	6	6	0
Pyrrhocoridae	3	5	0
Aleyrodidae	4	4	0
Others	49	52	0
Total：	116	139	0

**Table 7.** Statistics of Diptera in Macao.

Family	Genus	Species	Subspecies
Culicidae	10	36	0
Ceratopogonidae	3	10	0
Syrphidae	10	10	0
Chironomidae	4	7	0
Muscidae	6	7	0
Tephritidae	2	5	0
Calliphoridae	4	4	0
Sarcophagidae	2	4	0
Bombyliidae	2	2	0
Agromyzidae	1	1	0
Others	8	10	0
Total：	52	96	0

**Table 8.** Statistics of Orthoptera in Macao.

Family	Genus	Species	Subspecies
Catantopidae	13	14	0
Tettigoniidae	7	7	0
Oedipododae	5	6	0
Tetrigidae	5	5	0
Acrididae	4	4	0
Gryllidae	3	3	0
Gryllotalpidae	1	2	0
Arcypteridae	1	1	0
Gryllacridae	1	1	0
Pyrgomorphidae	1	1	0
Others	2	2	0
Total：	43	46	0

## References

[B10981829] ACC Ants-China.com. http://www.ants-china.com/index.html.

[B10959871] Achterberg C, Sharkey MJ, Chapman E G (2014). Revision of the genus *Euagathis* Szépligeti (Hymenoptera, Braconidae, Agathidinae) from Thailand, with description of three new species. Journal of Hymenoptera Research.

[B10960635] Ades G W Y, Kendrick R C (2004). Hong Kong fauna: A checklist of selected taxa. Fauna Conservation Department.

[B10982697] Barthélémy C, Lee J X, Kojima J (2014). Provisional distributional checklist of hong kong social wasps (Hymenoptera: Vespidae: Vespinae, Polistinae, Stenogastrinae). Hong Kong Entomological Society.

[B10959880] Barthélémy C, Olmi M (2019). Checklist of Dryinidae and Sclerogibbidae (Hymenoptera, Chrysidoidea) from Hong Kong. Zootaxa.

[B10631264] Brassard François, Leong Chi-Man, Chan Hoi-Hou, Guénard Benoit (2020). A new subterranean species and an updated checklist of *Strumigenys* (Hymenoptera, Formicidae) from Macao SAR, China, with a key to species of the Greater Bay Area. ZooKeys.

[B10631273] Brassard François, Leong Chi-Man, Chan Hoi-Hou, Guénard Benoit (2021). High diversity in urban areas: How comprehensive sampling reveals high ant species richness within one of the most urbanized regions of the world. Diversity.

[B10960667] Chen S C, He YH (2008). Phasmatodea of China.

[B11201039] DSEC Yearbook of Statistics 2022. https://www.dsec.gov.mo/getAttachment/6416a8ba-0221-433e-b86d-eac622c2105d/E_AE_PUB_2022_Y.aspx.

[B10981580] DSPA The Catalogue of Insect in the Cotai Ecological Zone. https://www.dspa.gov.mo/pdf/PB_20180718_CGIA_Place3_AL_009_INSECTA.pdf.

[B10959916] Easton E R (1991). Annotated list of insects of Macau observed during 1989. Entomological News.

[B10959925] Easton E R (1992). 1990 additions to the annotated list of the insects of Macau. Entomological News.

[B10960627] Easton E R (1993). The Insects of Macau.

[B10631291] Easton E. R., Pun W. W. (1996). New records of moths from Macau, Southeast China. Tropical Lepidoptera Research.

[B10631300] Easton E. R., Pun W. W. (1997). Observations on some Hemiptera: Heteroptera of Macau, Southeast Asia. Proceedings of the Entomological Society of Washington.

[B10631309] Easton E. R., Pun W. W. (1997). New records of butterflies from Macau, Southeast China (Lepidoptera: Papilionoidae). Tropical Lepidoptera.

[B10631282] Easton E. R., Liang G. Q. (2000). The Odonata of Macao, southern China. Notulae Odonatologicae.

[B10959966] Fang Z G, Wu S A, Xu H C (2001). A list of bamboos scale insects in China (Homoptera: Coccoidea). Journal of Zhejiang Forestry College.

[B10632034] Foottit R. G., Adler P. H. (2017). Insect biodiversity science and society.

[B10982685] Fu L, Huang G S, Li Z H, Wu X X, Kang F F, Lv WC, Fang Y (2012). The current and future potential geographic distribution of Dysmicoccusneobrevipes in China. Plant Quarantine.

[B10631318] Grimaldi D., Engel M. S. (2005). In Evolution of the Insects.

[B10982430] Guenard B S Ant diversity, from local to global scales: Effects of environmental conditions, community structure and biological invasions. https://www.proquest.com/dissertations-theses/ant-diversity-local-global-scales-effects/docview/1035140127/se-2.

[B10960021] Gumovsky A (2001). Taxonomic notes on the entedonine genera *Rhynchentedon* and *Pediobomyia* (Hymenoptera: Chalcidoidea: Eulophidae) with the description of a new species. Zoologische Mededelingen.

[B10959957] Heleodoro R A, Agudelo A A, Andreazze R (2016). The Mantodea (Dictyoptera: Insecta) of Rio Grande do Norte, Brazil: First List of Species and Geographical Records. EntomoBrasilis.

[B10981803] Heraty J, Woolley J, Polaszek A Catalog of the Encarsia of the World. http://www.faculty.ucr.edu/~heraty/Encarsia.cat.pdf.

[B10631337] Huang H. T., Li J. Q., Li Z. R., Jian H. B., Wu Z. Q., Huang J. Z. (2016). Study on species diversity and fauna of butterflies in Macao. Journal of Environmental Entomology.

[B10960689] Huang J (2019). Butterflies of Macao.

[B10960052] Huang J H, Zhou S Y (2006). A checklist of family Formicidae of China–Myrmicinae (Part I) (Insecta: Hymenoptera). Journal of Environmental Entomology.

[B10631348] Ishiwata K., Sasaki G., Ogawa J., Miyata T., Su Z. H. (2011). Phylogenetic relationships among insect orders based on three nuclear protein-coding gene sequences. Molecular Phylogenetics and Evolution.

[B10960370] Jäch M A, Easton E R, Jäch M A, Ji L (1998). Water beetles of China.

[B10960092] Jia F L (2014). A revisional study of the Chinese species of *Amphiops* Erichson (Coleoptera, Hydrophilidae, Chaetarthriini. Journal of Natural History.

[B10960072] Jia F L, Aston P, Fikáček M (2014). Review of the Chinese species of the genus *Coelostoma* Brullé, 1835 (Coleoptera: Hydrophilidae: Sphaeridiinae. Zootaxa.

[B10960063] Jia F L, Wang S S, Paul A (2018). Revision of *Chaetarthria* Stephens (Coleoptera: Hydrophilidae) in China, with a key to the species in the Oriental Region. Journal of Natural History.

[B10960475] Jiang Z Y, Zhao S, Yang Z Y, Jia F L, HÁJEK J (2022). A review of *Copelatus* Erichson, 1832 of Mainland China, with description of ten new species from the japonicus complex (Coleoptera: Dytiscidae: Copelatinae). Zootaxa.

[B10960454] Jiang Z Y, Zhao S, Jia F L, ŠŤASTNÝ J (2023). Two new species of *Platynectes* Régimbart, 1879 from China with notes on other Chinese members of the genus, including a key to species (Coleoptera: Dytiscidae: Agabinae). Zootaxa.

[B10631358] Johnson K. P., Dietrich C. H., Friedrich F., Beutel R. G., Wipfler B., Peters R. S., Allen J. M., Petersen M., Donath A., Walden K. K.O., Kozlov A. M., Podsiadlowski L., Mayer C., Meusemann K., Vasilikopoulos A., Waterhouse R. M., Cameron S. L., Weirauch C., Swanson D. R., Percy D. M., Hardy N. T., Terry I., Liu S. L., Zhou X., Misof B., Robertson H. M., Yoshizawa K. (2018). Phylogenomics and the evolution of hemipteroid insects.. Proceedings of the National Academy of Sciences.

[B10631390] Ke J. M., Mai X. Z. (2003). A new species of the *Toxorhynchites* (Diptera: Culicidae) from Macao, China. Port Health Control.

[B11246835] Kirkaldy G. W. (1909). A list of the Hemiptera of Oriental China Part l.. Annales de la Société entomologique de Belgique.

[B10982676] Kondo T, Palacino-Rodríguez F, Peña-Cuellar R D (2015). Report of *Erpetogomphussabaleticus* Williamson, 1918 (Odonata: Gomphidae) feeding on Diaphorinacitri Kuwayama (Hemiptera: Liviidae). Boletín del Museo de Entomología de la Universidad del Valle.

[B10959991] Kurahashi H, Chowanadisai L (2001). Blow flies (Insecta: Diptera: Calliphoridae) from Indochina. Species Diversity.

[B10960171] Lázaro S I C, Silva J H T, Freire R C Melo, Gama R A, Marcondes C B, Ximenes M F F Melo (2017). Checklist of mosquito species (Diptera: Culicidae) in the Rio Grande do Norte state, Brazil-contribution of entomological surveillance. Journal of medical entomology.

[B10631438] Leong C. M. (2017). Biological notes on the colony of *Brachyponeraobscurans* (Hymenoptera: Formicidae) in Macau. Taiwanese Journal of Entomological Studies.

[B10631420] Leong C. M., Shiao S. F., Guenard B. S. (2017). Ants in the city, a preliminary checklist of Formicidae (Hymenoptera) in Macau, one of the most heavily urbanized regions of the world. Asian Myrmecology.

[B10631429] Leong C. M., Yamane S., Guenard B. S. (2018). Lost in the city: discovery of the rare ant genus *Leptanilla* (Hymenoptera: Formicidae) in Macau with description of *Leptanillamacauensis* sp. nov. Asian Myrmecology.

[B10960153] Liang G Q, Easton E R (1995). A survey of Grasshopper from Macau. Supplement to the Journal of Sun Yatsen University.

[B10631447] Li J. Q., Huang H. T., Li Z. R., Jian H. B., Huang J. Z., Hu S. F., Zeng W. H. (2015). Study on diversity and fauna of Odonata in Macao. Guangdong Agricultural Science.

[B10631502] Lin M. Y., Perissinotto R., Clennell L. (2021). Census of the longhorn beetles (Coleoptera, Cerambycidae and Vesperidae) of the Macau SAR, China. ZooKeys.

[B10960131] Li S J, Xue X, Ahmed M Z, Ren S X, Du. Y Z, Wu J H, Cuthberston A G S, Qiu B L (2011). Host plants and natural enemies of *Bemisiatabaci* (Hemiptera: Aleyrodidae) in China. Insect Science.

[B10960101] Liu H C, Ma C H, Fikáček M, Wang L J (2021). Annotated catalogue of the water scavenger beetles from Orchid Island, Taiwan (Coleoptera: Hydrophilidae). Japanese Journal of Systematic Entomology.

[B10631491] Li Z. Q., Liu B. R., Zeng W. H., Zhang S. J., Wu J. F., Hu S. F. (2018). Assessing termite assemblages susceptibility to meteorological temperatures in a lower subtropical urban forest ecosystem in Macau. Journal of Environmental Entomology.

[B10960288] Li Z Q, Tang H Q (2021). Two new species of *Paratanytarsus* Thienemann & Bause (Diptera: Chironomidae) from Oriental China. Zootaxa.

[B10631483] Li Z. Q. (2023). Moths in Macao.

[B10960414] Mai Z Q, Hu J, Jia. F L (2022). Additional fauna of *Coelostoma* Brullé, 1835 from China, with re-establishment of *Coelostomasulcatum* Pu, 1963 as a valid species (Coleoptera, Hydrophilidae, Sphaeridiinae). ZooKeys.

[B10960432] Mai Z Q, Hu J, Minoshima Y N, Jia F L, Fikáček M (2022). Review of *Dactylosternum* Wollaston, 1854 from China and Japan (Coleoptera, Hydrophilidae, Sphaeridiinae). Zootaxa.

[B10975682] MBD Macao BioDatabase. https://nature.iam.gov.mo/BioDatabase/c/catlist?category=Insecta.

[B10631977] McKenna D. D., Farrell B. D. (2010). 9-genes reinforce the phylogeny of holometabola and yield alternate views on the phylogenetic placement of Strepsiptera. PLOS One.

[B10960224] Mendes L F (2002). New species and new data on Protrinemuridae and Nicoletiidae (Zygentoma) from Eastern Asia and Pacific islands. Annales de la Société Entomologique de France.

[B10631527] Mendes L. F., Sousa A. B. (2007). Note on some butterflies (Lepidoptera) of India and of Macau (China). Boletim da Sociedade Portuguesa de Entomologia.

[B10631559] Misof B., Liu S., Meusemann K., Peters R. H., Donath A., Mayer C., Frandsen P. B., Ware J., Flouri T., Beutel R. G., Niehuis O., Petersen M., Izquierdo-Carrasco F., Wappler T., Rust J., Aberer A. J., Aspöck U., Bartel D., Blanke A., Berger S., Böhm A., Buckley T. R., Calcott B., Chen J. Q., Friedrich F., Fukui M., Fujita M., Greve C., Grobe P., Gu S. C., Hunag Y., Jermiin L. S., Kawahara A. Y., Krogmann L., Kubiak M., Lanfear R., Letsch H., Li Y. Y., Li Z. Y., Li J. G., Lu H. R., Machida R., Mashimo Y., Kapli P., Mckenna D. D., Meng G. L., Nakagaki Y., Navarrete-Heredia J. L., Ott M., Ou Y. X., Pass G., Podsiaadlowski L., Pohl H., Reumont B. M., Schütte K., Sekiya K., Shimizu S., Slipinski A., Stamatakis A., Song W. H., Su X., Szucsich N. U., Tan M. H., Tan X. M., Tang M., Tang J. B., Timelthaler G., Tomizuka S., Trautwein M., Tong X. L., Uchifune T., Walzl M. G., Wiegmann B. M., Wilbrandt J., Wipfler B., Wong T. K. F., Wu Q., Wu G. X., Xie Y. L., Yang S. Z., Yang Q., Yeates D. K., Yoshizawa K., Zhang Q., Zhang R., Zhang W. W., Zhang Y. H., Zhao J., Zhou J., Zhou L. L., Ziesmann T., Zou S. J. (2014). Phylogenomics resolves the timing and pattern of insect evolution. Science.

[B10631703] Mo J. Y., Li K. H., Guo T. X. (2006). The preliminary survey report of gardens-virescence-plants pests and diseases in Macao District. Guihaia.

[B10960250] Nguyen L T P, Carpenter J M (2020). Potter wasps of the genus *Labus* (Hymenoptera, Vespidae, Eumeninae) from Vietnam, with description of two new species. Journal of Hymenoptera Research.

[B10960259] Niitsuma H, Tang H Q (2019). Taxonomic review of *Ablabesmyia* Johannsen (Diptera: Chironomidae: Tanypodinae) from Oriental China, with descriptions of six new species. Zootaxa.

[B10631712] Perissinotto P., Yik F. P.L., SFH Pun (2023). New records of the Fluffy Tit butterfly, *Hypolycaenaamasa* Hewitson, [1865] (Lepidoptera: Lycaenidae: Theclinae), from Macau and Hong Kong.. Oriental Insects.

[B10631721] Perissinotto R., Clennell L. (2021). Census of the fruit and flower chafers (Coleoptera, Scarabaeidae, Cetoniinae) of the Macau SAR, China. ZooKeys.

[B10631730] Perissinotto R., Lu Y. Y. (2022). Shining leaf chafers (Scarabaeidae: Rutelinae) and rhino beetles (Scarabaeidae: Dynastinae) of the Macau SAR, China. Oriental Insects.

[B10631739] Peters R. S., Meusemann K., Petersen M., Mayer C., Wilbrandt J., Ziesmann T., Donath A., Kjer K. M., Aspöck U (2014). The evolutionary history of holometa-bolous insects inferred from transcriptome-based phylogeny and comprehensive morphological data. BMC Evolutionary Biology.

[B10981786] Przewoźny M Catalogue of Palearctic Hydrophiloidea (Coleoptera). http://www.waterbeetles.eu/documents/PAL_CAT_Hydrophiloidea_2017.pdf.

[B10631956] Pun W W, Batalha C D C (1997). Manual de insect de Macau..

[B10631795] Qu F. Y. (2001). On the records mosquitoes, Sandeflies, Blackflies in Macau, China. Acta Parasitology et Medica Entomologica. Sinica.

[B10631965] Ramos H C, Ribeiro H, Novo M T, Easton E R (1997). Os mosquitoes de Macau (Diptera: Culicidae)..

[B10631813] Ribeiro H. (1997). New species of *Toxorhynchites* (Diptera: Culcidae) from Macau (China). Journal of the American Mosquito Control Association-Mosquito News.

[B10982665] Shahbazvar N, Sahragard A, Manzari S, Hosseini R, Hajizadeh H (2010). A faunal study of whiteflies (Hemiptera: Aleyrodidae) and their parasitoids in Guilan province, Iran. Entomofauna.

[B10631822] Shen X. C. (2015). Insect Geography of China.

[B10631831] Sun H., Gong B., Ke M. J., Wu Z. X., Guo Q. L., Yu Y. X. (2010). Two new species of the *Dasyhelea* (Diptera, Ceratopogonidae) from Macao, China. Acta Parasitology et Medica Entomologica.

[B10960277] Tang H G, Cranston P S, Zhao J G, Lok C W, Wong K C, Li Z Q (2014). The immature stages of Polypedilum (Pentapedilum) nodosum (Johannsen) and Polypedilum (Tripodura) masudai (Tokunaga) (Diptera, Chironomidae, Chironominae). Zootaxa.

[B10960268] Tang H Q, Cranston P S (2017). Review of *Nilodosis* Kieffer (Diptera: Chironomidae: Chironominae), with description of a new species from South China. Zootaxa.

[B10981588] Teng C L, Liu Q, Cheng Y M, Hu C H, Li J H, Zhang Y X, Huang X L, Zhu A H (2022). Preliminary Investigation on the Diversity and Fauna Analysis of Tetrigoidea in Xiangxi Region. International Journal of Ecology.

[B10982639] Tsai J F, Redei D (2009). The identity of jewel bugs described by Shonen Matsumura from Taiwan (Hemiptera: Heteroptera: Scutelleridae. Zootaxa.

[B10631842] Wang Y. H., Engel M. S., Rafael J. A., Wu H. Y., Rédei D., Xie Q., Wang G., Liu X. G., Bu W. J. (2016). Fossil record of stem groups employed in evaluating the chronogram of insects (Arthropoda: Hexapoda). Scientific Reports.

[B10631859] Wang Y. H., Luan Y. X., Lou J. Y., Men Y., Engel M. S., Damgaard J., Khila A., Chen P. P., Moreira F. F.F., Rafael J. A., Xie Q. (2023). 300 Million years of coral treaders (Insecta: Heteroptera: Hermatobatidae) back to the ocean in the phylogenetic context of Arthropoda. Proceedings of the Royal Society B.

[B10982412] Wetterer J K, Lubertazzi D, Wilson E O (2019). Ants of grenada (Hymenoptera, Formicidae. Bulletin of the Museum of Comparative Zoology.

[B10982050] Wheeler W M (1930). A list of the known Chinese ants. Peking Natural History Bulletin.

[B10631891] Wilson K. D.P., Xu Z. F. (2007). Odonata of Guangdong, Hong Kong and Macau, South China, part 1: Zygoptera. International Journal of Odonatology.

[B10631900] Wipfler B., Letsch H., Frandsen P. B., Kapli P., Mayer C., Bartel D., Buckley T. R., Donath A., Edgerly-Rooks J. S., Fujita M., Liu S. L., Machida R., Mashimo Y., Misof B., Niehuis O., Peters R. S., Petersen M., Podsiadlowski L., Schütte K., Shimizu S., Uchifune T., Wilbrandt J., Yan E., Zhou X., Simon S. (2019). Evolutionary history of Polyneoptera and its implications for our understanding of early winged insects. Proceedings of the National Academy of Sciences.

[B10631930] Wong T. L., Guénard B (2020). Review of ants from the genus *Polyrhachis* Smith (Hymenoptera: Formicidae: Formicinae) in Hong Kong and Macau, with notes on their natural history. Asian Myrmecology.

[B10631939] Xiao W. J. (2012). A Formiga Branca em Macau..

[B10960645] Yang D, Zang L L, Zhang K Y (2018). Species Catalogue of China. Animals, Insecta (VI), Diptera(2), Orthorrhaphous Brachycera.

[B10960334] Yang J K (1997). Four new and rare species of mantids from Yunnan China (Insecta: Mantodea. Journal of Yunnan Agricultural University.

[B10960323] Yang J W, Xu Z H, Mei X X, Zhang J L, Zhao Y X (2004). Taxonomy of ants on the eastern slope of Xishan Mountains in Kunming. Journal of Southwest Forestry College.

[B10960297] Yang L, Chen W H, Zhang J, Chen Y., Liu S Y, Chen Y J, Zheng D Y, Liang X, Wu H Z, Qing N, Lu W H, Lutman R (2011). Summer butterfly diversity in Dinghushan nuture reserve of Guangdong province. Journal of South China Normal University (Natural Science Edition.

[B10960314] Yang Z M, Jia F L, Tang Y D, Jiang L (2021). Two new species of *Helochares*, with additional faunistic records from China (Coleoptera, Hydrophilidae, Acidocerinae). ZooKeys.

[B10631947] Yu Y. X., Huang Y. Y. (2006). New species and records of biting midges from Macau (Diptera: Ceratopogonidae). Acta Parasitology et Medica Entomologica Sinica.

[B10960659] Zhang Y (2006). The analyses of the species diversity and faunal distribution of Coleoptera in Beijing-Tianjin-Hebei of China.

